# Adolopment of clinical practice guidelines and creation of referral pathways for psychiatric conditions in Pakistan

**DOI:** 10.1016/j.lansea.2024.100387

**Published:** 2024-03-10

**Authors:** Alina Pervez, Muhammad Murtaza Bukhari, Rijah Chhapra, Meryum Ishrat Baig, Russell Seth Martins, Sonia Pirzada, Nashia Ali Rizvi, Salima Saleem Aamdani, Bushra Ayub, Alina Abdul Rehman, Mohsin Ali Mustafa, Sarah Nadeem, Nargis Asad, Adil H. Haider, Tania Nadeem

**Affiliations:** a Center for Clinical Best Practices, Clinical and Translational Research Incubator, Aga Khan University, Karachi, Pakistan; b Medical College, Aga Khan University, Karachi, Pakistan; c University of Kentucky, Lexington, KY, USA; d Department of Medicine, Aga Khan University, Karachi, Pakistan; e Learning Research Centre, Patel Hospital, Karachi, Pakistan; f Section of Endocrinology, Department of Medicine, Aga Khan University, Karachi, Pakistan; g Department of Psychiatry, Aga Khan University, Karachi, Pakistan

**Keywords:** Psychiatry, Clinical practice guidelines, Pakistan, Referral

## Abstract

Psychiatric disorders are highly prevalent in Pakistan and burdens the scarce number of psychiatrists present in the country. The establishment of evidence-based clinical practice guidelines (EBCPGs) and primary-care referral pathways within the local context is imperative to make the process efficient. In this Health Policy, we aimed to develop EBCPGs and primary-care referral pathways that are specific to Pakistan's primary-care setting, with the aim of facilitating the management of psychiatric conditions. Ten EBCPGs were created through the GRADE-ADOLOPMENT process; two recommendations were adopted with minor changes, 43 were excluded, and all others were adopted without any changes. Ten primary-care referral pathways for managing ten psychiatric disorders were created and 23 recommendations were added which will help to bridge the gap in care provision. These psychiatric referral pathways and EBCPGs will bring Pakistan's healthcare system a step closer to achieving optimal health outcomes for patients.

## Introduction

By 2030, the WHO estimates that mental health issues will become the leading contributor to rising morbidities in the modern world.^1^ Currently, mental health disorders account for 10–13% of the global disease burden,^2^ with a worrying proportion of patients not receiving adequate treatment. This unmet need for psychiatric care can be as high as 90% in low-income and middle-income countries (LMICs) like Pakistan. Pakistan's mental health indicators are particularly concerning, as the country has a considerable burden of psychiatric disorders but spends a meagre 0.4% of its budget on mental health.^3^ While no official numbers exist, Pakistan has approximately 400 psychiatrists across a population of more than 220 million, with most psychiatrists being based in urban areas.^4^ This leaves many people from rural settings and slum areas around major urban centres without adequate access to psychiatric care. An estimated 50 million or more people in Pakistan may be afflicted with mental disorders.^5^ The meagre annual household income per capita of around 600 US$ ensures many of these patients either go untreated or are forced to turn to faith healers and homeopathic remedies.^6^ A dire lack of funding and poor handling of existing funds further exacerbates the situation.^5^

Evidence-based clinical practice guidelines (EBCPGs) provide a clear and consistent framework for patient care, basing recommendations off a comprehensive synthesis of high-quality evidence. Globally, several EBCPGs exist, mostly from high-income countries in the western part of the globe, which offer detailed management recommendations for most major psychiatric illnesses. Reliance on these guidelines may help to ensure that uniformity may be achieved in the management of certain psychiatric conditions within a country's healthcare system. However, when implementing these guidelines in different regions, it is important to keep in mind that the psychosocial aspects of psychiatric care can be expected to vary across contexts and settings. Cultural influences have a widespread impact on psychiatric care, particularly for non-pharmacological aspects of management. Additionally, socioeconomic status directly affects the quality of psychiatric care that a patient can afford or access.

LMICs struggle to develop local psychiatry EBCPGs, mainly due to limitations in financial resources, high-quality local evidence, resource infrastructure, and available expertise. This is worrisome, as psychiatric healthcare in LMICs differs in several respects, including insufficient budget allocations, stigmatisation of mental health-related issues, shortage of trained professionals, and inadequate mental health policy. The Mental Health Atlas survey conducted by WHO in 2014 demonstrated that only 24% of countries across the world had independent policies pertaining to mental health.^7^ This figure is particularly concerning for the African continent, where 46% of countries lacked a stand-alone mental health policy. This, combined with the alarming increase in population and treatment gaps for psychiatric conditions in LMICs in Africa, should serve as a persuasive impetus for national policymakers and researchers to implement standardised psychiatric clinical practice guidelines in their health system.^8^

Pakistan is one such LMIC that lacks local psychiatric EBCPGs, and the unpresented use of non-local EBCPGs (developed for the healthcare contexts of other countries) presents the issue of unaddressed region-specific concerns in clinical practice. The Pakistan Psychiatric Society has previously published informational resources regarding psychiatric conditions in Pakistan. However, these resources lack clarity regarding the methodology underlying their development, which may limit their credibility and applicability. In addition, they only developed guidelines for eight disorders.^9^ Therefore, the next most suitable approach for resource-constrained LMICs like Pakistan is to apply context-specific modifications to high-quality internationally developed EBCPGs. The optimal modification process should be based on a combination of adoption (assimilating existing recommendations as is), adaptation (modification of selected recommendations following critical evaluation), and exclusion (omitting recommendations deemed irrelevant to the local context) of existing EBCPGs.^10^ “Adolopment” is a recently introduced word that encompasses three key elements of adoption, adaptation, and development. The GRADE (Grading of Recommendations, Assessment, Development, and Evaluation)-ADOLOPMENT^10^ framework uses evidence to decision (EtD) tables to guide this process.^11^ EtD tables provide general and context-specific evidence across standard criteria (Supplementary Table S1), against which experts judge the appropriateness of existing recommendations and proposed modifications. These modifications may be in the form of a change to the specific population, intervention, or control compared to the original recommendation.

As the disease burden in Pakistan increases, the management of mental illnesses has increasingly become a responsibility of general practitioners (GPs), and the evidence suggests that patient outcomes may remain at par with specialist care for mild to moderate severity illnesses.^12^ Consequently, there is an immense need for local psychiatry EBCPGs to be developed by following a transparent, standardised process that uses existing and available best-evidence EBCPGs with appropriate context-specific modifications for use by GPs. In addition, a primary care management and referral pathway in the form of a quick and easy-to-follow algorithm will assist GPs in making rapid and informed decisions. Such referral pathways and EBCPGs would bring the healthcare system of Pakistan a step closer to achieving optimal health outcomes in psychiatry and would have greater credibility by virtue of their transparent development processes. Thus, we aimed to employ the GRADE-ADOLOPMENT process to develop local evidence-based EBCPGs and primary care algorithms for managing common adult psychiatric conditions by GPs in Pakistan.

## Methods

### Study setting

This initiative was conducted at the CITRIC (Clinical and Translational Research Incubator) Center for Clinical Best Practices (CCBP) at the Aga Khan University Hospital (AKUH) in Pakistan. AKUH is a private, non-profit institute, and is one of Pakistan's leading medical and biomedical research institutes. AKUH's CITRIC CCBP deals with the adaptation and development of EBCPGs and care channels to standardise and improve healthcare in Pakistan and other LMICs. The GRADE-ADOLOPMENT processes outlined in this study was employed by the CCBP, in collaboration with the Department of Psychiatry at AKUH, and the GRADE-USA networking group, in the development of EBCPGs for the management of common psychiatric disorders at the level of primary care in Pakistan. The team has used a similar process to create local guidelines for the management of chronic respiratory conditions and type-2 diabetes in Pakistan.^13^^,^^14^ The decision to develop these guidelines for GPs rather than psychiatrists is due to the growing global trend in mental health care being offered by GPs, as well as due to a dearth of specialists in Pakistan.^15^ Given the lack of involvement of patients or other human participants, a waiver of ethics approval and informed consent was obtained from the Ethics Review Committee of the Aga Khan University. All methods were conducted in accordance with the highest ethical standards outlined in the 1964 Declaration of Helsinki and its future amendments.

### Study team

The study team was comprised (i) CCBP staff, who have extensive experience in the development of management EBCPGs, and (ii) Department of Psychiatry at AKUH which included four senior attending psychiatrists (more than four years of practice) and the Head, Department of Psychiatry.

### Source guideline selection

Supplementary Figure SA depicts the process followed to create the guidelines and referral pathways. The source guideline is the single, original, “parent” EBCPG that underwent the GRADE-ADOLOPMENT process in the development of a local EBCPG. Three local expert psychiatrists evaluated available guidelines by performing an extensive literature review on Google Scholar and PUBMED from January 2000 to December 2021. The local expert psychiatrists considered local familiarity, scope, applicability, thoroughness, and integrity of creating bodies for each EBCPG. After careful consideration, the experts decided to use ten American Psychiatric Association (APA) guidelines as the source guidelines and to standardise the local guidelines because the APA guidelines are the most used and familiar guideline locally and were assessed to have good methodological quality. However, for the delirium guidelines, to incorporate the neurological perspective, the European Federation of the Neurological Societies guideline was also used which fulfilled the same criteria for selection. The following ten openly available EBCPGs developed by APA, and one developed by the European Federation of the Neurological Societies were chosen to undergo the GRADE-ADOLOPMENT process.

• *Practice Guideline for the Treatment of Patients with Bipolar Disorder (revision)—2002**^16^*
• *Practice Guideline for the Treatment of Patients with Obsessive-Compulsive Disorder*—*2007**^17^*
• *Practice Guideline for the Treatment of Patients with Schizophrenia (Third Edition)—2020**^18^*
• *Practice Guideline for the Treatment of Patients with Acute Stress Disorder and Posttraumatic Stress Disorder—2004**^19^*
• *Practice Guideline for the Treatment of Patients with Major Depressive Disorder—2010**^20^*
• *Practice Guideline for the Treatment of Patients with Panic Disorder—2009**^21^*
• *Practice Guideline for the Treatment of Patients with Delirium—2010**^22^*
• *Practice Guideline for the Treatment of Patients with Alzheimer's disease and Other Dementias—2007**^23^*
• *EFNS guidelines for the diagnosis and management of Alzheimer's Disease. European Journal of Neurology 2010**^24^*
• *Practice Guideline for the Treatment of Patients with Eating Disorders—2006**^25^*
• *Practice Guideline for the Treatment of Patients with Substance Use Disorders 2007**^26^*

These were selected as the source EBCPGs due to their comprehensive set of recommendations, integrated approach to management, and high-quality synthesis of available evidence.

### Guideline content review

A Table of Recommendations (ToR) was made by extracting and compiling all recommendations mentioned in the source EBCPGs. Two senior attending psychiatrists reviewed each ToR independently and marked each recommendation as either “Adopt”, “Adapt” or “Exclude.” Discrepancies were settled in consensus with the Head, Department of Psychiatry. Recommendations marked “Adopt” were incorporated into the local EBCPG with no further changes or with minor changes, while those marked “Exclude” were omitted from the local EBCPG. Exclusions were based on recommendations pertaining to pediatric and inpatient, healthcare services or medications unavailable in Pakistan, or if otherwise deemed irrelevant to the local context. Recommendations marked “Adapt” were planned to undergo review and revision via the GRADE-ADOLOPMENT process via GRADEPro (Supplementary Material) before incorporation into local EBCPGs.

Our adolopment process had some differences from the original process. In contrast to the original process^10^ where every recommendation was subjected to complete adaptation process via EtD tables and expert panel review, we adopted recommendations with minor changes that only required minor and straightforward changes, which did not affect the recommendation's meaning but rather provided supporting information or clarification. Based on the expert review, we concluded that no recommendation needed adaptation.

Following the creation of the guidelines, we sought to ensure the validity and comprehensiveness of the local guideline by engaging an external psychiatry expert. The expert was a Professor and Head of the Department of Psychiatry at one of the major private medical colleges in the country. The expert was invited to review the modifications made to the source guideline and provide their professional assessment. Changes, if any, were discussed in collaboration with the expert Psychiatrists at AKU and subsequently synthesised into the final version of the local guidelines.

### Referral care pathway creation

The Head, Department of Psychiatry worked with the CCBP faculty to develop management algorithms for primary care providers. A draft of the clinical referral pathway was made using the recommendations found in the EBCPGs. However, if any gaps were identified during the formation of the clinical management and referral pathways, additional recommendations were sought through a best-evidence systematic review process (Supplementary Material), including pre-existing EBCPGs. If suitable pre-existing EBCPGs were not found, we integrated recommendations using peer-reviewed evidence from reputable information sources. The evidence used to develop each recommendation was reviewed by the local experts and the final version of the primary care referral pathway was made.

### Final debriefing to identify challenges and explore solutions

Two focus group discussions (FGDs) were held to highlight problems encountered during the guideline creation process and to propose potential solutions. A CCBP team member served as the FGD leader. The moderator was chosen due to their qualifications and experience, including a medical degree, training in GRADE methodology, and a postgraduate public health degree. The CCBP staff, four senior attending psychiatrists from the Department of Psychiatry, a scribe and the moderator participated in the FGDs. Both FGDs were conducted in English language and were an hour long. The scribe recorded the audio of the session and took notes. Prior to the FGD, participants were given the chance to independently produce problems and potential solutions faced during the process which were then discussed during the meeting. After the session was complete, two researchers went through the scribes’ notes and the audio recording to identify and list the challenges and solutions and each challenge was classified as either a major or minor challenge according to the consensus. The final list of specific difficulties was then categorised by the CCBP team into broad themes, and the related negatory solutions were then provided alongside them.

## Results

**Fig. 1:**
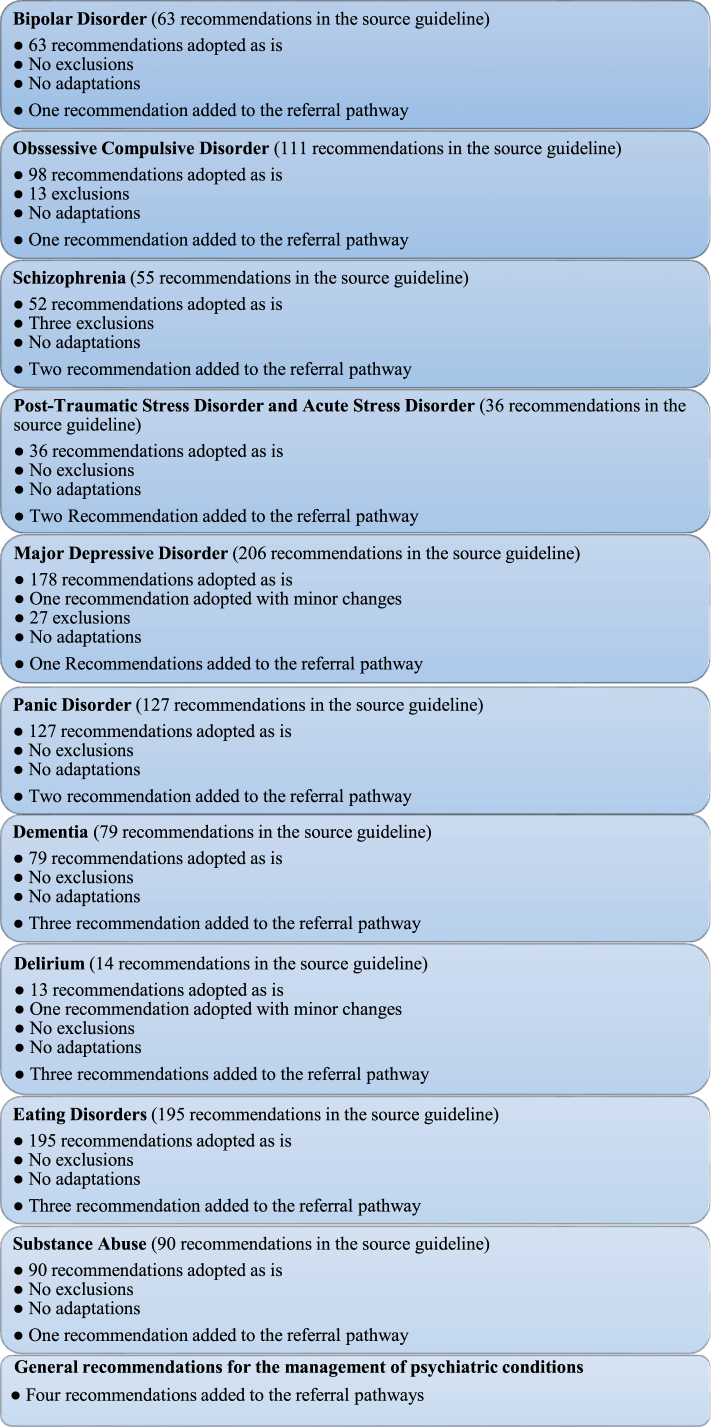
**Outcomes of Psychiatry evidence-based clinical practice guideline (EBCPG) and referral pathways**.

Ten EBCPGs were created through the GRADE-ADOLOPMENT process; two recommendations were adopted with minor changes, 43 were excluded, and all others were adopted as is into the local EBCPGs (Fig. 1 and Table 1). The complete EBCPG can be found in the Supplementary Material. Ten primary-care referral pathways for managing these psychiatric disorders were also created. The focus was on early detection and diagnosis, primary care management, and prompt referral to specialists. 23 recommendations were added across the 10 referral pathways (Fig. 1, Table 1, and Supplementary Figures S1–S10).

**Table 1:** Recommendations adopted with minor changes or excluded in the evidence-based clinical practice guideline (EBCPG) and recommendations added to the referral pathways for management of psychiatric conditions in Pakistan (text in bold indicates it has been added or modified).

Action	Recommendation	Source or reason for exclusion
General recommendations for the management of psychiatric conditions		
Added	Combination of 2 SSRIs or SSRI with SNRI does not augment treatment	Insufficient evidence exists to support augmentation APA^20^
Added	Before starting, ensure patients are aware that SSRIs take 2–4 weeks before the effects start to appear	National Institute for Health Care and Excellence,^27^ National Health Service^28^
Added	To evaluate the risk of suicide or self-harm, use Triage Tool. Patients can also be evaluated using ASQ tool and the Beck Scale for Suicide Ideation (BSI) to determine risk of suicidality. BSI may be used in Urdu when required^29^	National Institute for Occupational Safety and Health,^30^ National Institute of Mental Health^31^
Added	To assess patient's tendency to cause harm to others, use assault and homicidal danger assessment tool or indicator, such as Triage Tool. The Danger Assessment Tool can also be used to assess risk to healthcare personnel	National Institute for Occupational Safety and Health^30^
Bipolar Disorder		
Added	Counsel regarding medication use, side effects, and disease or treatment related misconceptions (if any)	National Institute for Health Care and Excellence^32^
Obsessive Compulsive Disorder		
Added	Counsel regarding medication use, side effects, and disease or treatment related misconceptions (if any)	National Institute for Health Care and Excellence,^33^ American Family Physician,^34^ National Institute of Mental Health^35^
Excluded	“When OCD is of disabling severity, the psychiatrist may need to write on the patient's behalf to government agencies that control access to disability income, publicly financed health care, or government-supported housing; or to tax authorities, courts, schools, or employers [I]”	Reason for exclusion: Unavailable in Pakistan
Excluded	“There are no controlled studies that demonstrate effectiveness of dynamic psychotherapy or psychoanalysis in dealing with the core symptoms of OCD. Psychodynamic psychotherapy may still be useful in helping patients overcome their resistance to accepting a recommended treatment by illuminating their reasons for wanting to stay as they are (e.g., best adaptation, secondary gains) [III]”	Reason for exclusion: Beyond the scope of General Practitioner
Excluded	“It may also be useful in addressing the interpersonal consequences of the OCD symptoms [II]. Motivational interviewing may also help overcome resistance to treatment [III]”	Reason for exclusion: Beyond the scope of General Practitioner
Excluded	“Useful strategies to manage medication side effects include small doses of modafinil to minimize fatigue or sleepiness”	Reason for exclusion: Beyond the scope of General Practitioner
Excluded	“Clinicians should consider booster sessions for more severely ill patients, for patients who have relapsed in the past, and for patients who show signs of early relapse [II]”	Reason for exclusion: Beyond the scope of General Practitioner
Excluded	“Combined SRI and CBT treatment may be provided when the patient has a co-occurring disorder that is SRI-responsive [I]”	Reason for exclusion: Beyond the scope of General Practitioner
Excluded	“Another option in the case of partial response to ERP therapy is to increase the intensity of treatment (e.g., from weekly to daily sessions) [III]”	Reason for exclusion: Beyond the scope of General Practitioner
Excluded	“Some evidence suggests that adding cognitive therapy to ERP may enhance the results, but this remains to be established [III]”	Reason for exclusion: Beyond the scope of General Practitioner
Excluded	“After first- and second-line treatments and well-supported augmentation strategies exhausted, can consider augmenting SSRIs with clomipramine, buspirone, pindolol, riluzole, or once-weekly oral morphine sulfate”	Reason for exclusion: Beyond the scope of General Practitioner
Excluded	“However, morphine sulfate should be avoided in patients with contraindications to opiate administration, and appropriate precautions and documentation should occur. If clomipramine is added, appropriate precautions should be utilized with regard to preventing potential cardiac and central nervous system side effects [I]”	Reason for exclusion: Beyond the scope of General Practitioner
Excluded	“Less well-supported monotherapies to consider include d-amphetamine, Tramadol, Monoamine oxidase inhibitors, Ondansetron, Transcranial magnetic stimulation, and deep brain stimulation [III]”	Reason for exclusion: Beyond the scope of General Practitioner
Excluded	“Intensive residential treatment or partial hospitalization may be helpful for patients with severe treatment-resistant OCD [II]”	Reason for exclusion: Beyond the scope of General Practitioner
Excluded	“Ablative neurosurgery for severe and very treatment-refractory OCD is rarely indicated and, along with deep brain stimulation, should be performed only at sites with expertise in both OCD and these treatment approaches [III]”	Reason for exclusion: Beyond the scope of General Practitioner
Schizophrenia		
Added	Counsel regarding medication use, side effects, and disease or treatment related misconceptions (if any)	National Institute for Health Care and Excellence,^36^ National Health Service,^37^ American Journal of Psychiatry^38^
Added	Monitor neutrophil count weekly during the first six months of clozapine administration, every other week for the second six months and every four weeks after one year for the duration of treatment. Also monitor closely for symptoms of myocarditis during initial four weeks of treatment	National Health Service,^39^ National Institute for Health Care and Excellence^40^
Excluded	“Patients who have moderate to severe or disabling tardive dyskinesia associated with antipsychotic therapy be treated with a reversible inhibitor of the vesicular monoamine transporter 2 (1B)”	Reason for exclusion: Unavailable in Pakistan
Excluded	“Patients with schizophrenia be treated with cognitive behavioral therapy for psychosis (1B)”	Reason for exclusion: Beyond the scope of a General Practitioner
Excluded	“Patients with schizophrenia receive supported employment services (1B)”	Reason for exclusion: Unavailable in Pakistan
Post-Traumatic Stress Disorder and Acute Stress Disorder		
Added	Counsel regarding medication use, side effects, and disease or treatment related misconceptions (if any)	National Institute for Health Care and Excellence,^41^ American Psychological Association,^42^ National Institute of Mental Health^43^
Added	Counsel regarding the importance of family in supporting patient, and encourage the practice of religion and spirituality	National Institute of Mental Health,^43^ US Department of Veterans Affairs^44^
Major Depressive Disorder		
Added	Continue medications for nine months, or six months after stability achieved	National Institute for Health Care and Excellence^27^ American Psychiatry Association,^45^ National Institute of Mental Health^46^
Adopt with Minor Changes	“Treatment in the acute phase may include pharmacotherapy, depression-focused psychotherapy, and combination of pharmacological and psychotherapy **or provide adequate referral** for other somatic therapies such as electroconvulsive therapy (ECT), transcranial magnetic stimulation (TMS), or light therapy. Selection of an initial treatment modality should be influenced by clinical features (e.g., severity of symptoms, presence of co-occurring disorders or psychosocial stressors) as well as other factors (e.g., patient preference, prior treatment experiences)”	Changes made: Refer for ECT, TMS, and Light Therapy as they are beyond the scope of a general physician. Source: American Psychiatry Association,^45^ National Institute of Mental Health^47^ UpToDate^48^
Excluded	“Patients who refuse inpatient treatment can be hospitalized involuntarily if their family is on board. Condition meets the criteria of the local jurisdiction for involuntary admission [I]”	Reason for exclusion: no laws for involuntary admission
Excluded	“Admission to a hospital or, if available, an intensive day program, may also be indicated for severely ill patients who lack adequate social support outside of a hospital setting, who have complicating psychiatric or general medical conditions, or who have not responded adequately to outpatient treatment [I]”	Reason for exclusion: no day programs available in Pakistan
Excluded	“In general, the use of nonselective monoamine oxidase inhibitors (MAOIs) (e.g., phenelzine, tranylcypromine, isocarboxazid) should be restricted to patients who do not respond to other treatments [I], given the necessity for dietary restrictions with these medications and the potential for deleterious drug–drug interactions”	Reason for exclusion: nonselective MAOIs are unavailable
Excluded	“Use of a depression-focused psychotherapy alone is recommended as an initial treatment choice for patients with mild to moderate major depressive disorder [I]”	Reason for exclusion: Beyond the scope of General Practitioner
Excluded	“Clinical evidence supporting the use of cognitive behavioral therapy (CBT) [I] Interpersonal psychotherapy [I] Psychodynamic therapy [II] Problem-solving therapy [III] In individual [I] and in group [III] formats”	Reason for exclusion: Beyond the scope of General Practitioner
Excluded	“Considerations in the choice of a specific type of psychotherapy include the goals of treatment (in addition to resolving major depressive symptoms), prior positive response to a specific type of psychotherapy, patient preference, and the availability of clinicians skilled in the specific psychotherapeutic approach [II]”	Reason for exclusion: Beyond the scope of General Practitioner
Excluded	“When determining the frequency of psychotherapy sessions for an individual patient, the psychiatrist should consider multiple factors, including the specific type and goals of psychotherapy, symptom severity (including suicidal ideas), co-occurring disorders, cooperation with treatment, availability of social supports, and the frequency of visits necessary to create and maintain a therapeutic relationship, ensure treatment adherence, and monitor and address depressive symptoms and suicide risk [II]”	Reason for exclusion: Beyond the scope of General Practitioner
Excluded	“For patients in psychotherapy, additional factors to be assessed include the frequency of sessions and whether the specific approach to psychotherapy is adequately addressing the patient's needs [I]”	Reason for exclusion: Beyond the scope of General Practitioner
Excluded	“With some TCAs, a drug blood level can help determine if additional dose adjustments are required [I]”	Reason for exclusion: Unavailable in Pakistan
Excluded	“Particularly for those who have shown minimal improvement or experienced significant medication side effects, other options include augmenting the antidepressant with a depression-focused psychotherapy [I] or with other agents [II]”	Reason for exclusion: Redundant
Excluded	“Medication can be changed to non-MAOI antidepressant in patients with minimal improvement or those with significant medication side effects”	Reason for exclusion: Unavailable in Pakistan
Excluded	“In patients capable of adhering to dietary and medication restrictions, an additional option is changing to a nonselective MAOI [II]”	Reason for exclusion: Beyond the scope of General Practitioner
Excluded	“Enough time should be provided between medication to avoid deleterious interactions”	Reason for exclusion: Beyond the scope of General Practitioner
Excluded	“Transdermal selegiline, a relatively selective MAO B inhibitor with fewer dietary and medication restrictions, or transcranial magnetic stimulation could also be considered [II]”	Reason for exclusion: Unavailable in Pakistan
Excluded	“Vagus nerve stimulation (VNS) may be an additional option for individuals who have not responded to at least four adequate trials of antidepressant treatment, including ECT [III]”	Reason for exclusion: Unavailable in Pakistan
Excluded	“For patients treated with psychotherapy, consideration should be given to increasing the intensity of treatment or changing the type of therapy [II]”	Reason for exclusion: Redundant
Excluded	“If psychotherapy is used alone, the possible need for medications in addition to or in lieu of psychotherapy should be assessed [I]”	Reason for exclusion: Redundant
Excluded	“Patients who have a history of poor treatment adherence or incomplete response to adequate trials of single treatment modalities may benefit from combined treatment with medication and a depression-focused psychotherapy [II]”	Reason for exclusion: Redundant
Excluded	“Maintenance treatment with VNS is also appropriate for individuals whose symptoms have responded to this treatment modality [III]”	Reason for exclusion: Beyond the scope of General Practitioner
Excluded	“Catatonic features that occur as part of a major depressive episode should be treated with a barbiturate”	Reason for exclusion: Beyond the scope of General Practitioner, to be referred to a tertiary care setting for further management
Excluded	“When possible, a period of substance abstinence can help determine whether the depressive episode is related to substance intoxication or withdrawal [II]”	Reason for exclusion: Beyond the scope of General Practitioner
Excluded	“Selegiline has antiparkinsonian and antidepressant effects but may interact with l-dopa and with other antidepressant agents [I]”	Reason for exclusion: Unavailable in Pakistan
Panic Disorder		
Added	Counsel regarding medication use, side effects, and disease or treatment related misconceptions (if any)	National Institute for Health Care and Excellence,^49^ American Family Physician,^50^ National Institute of Mental Health,^51^ National Health Service^52^
Added	Continue medications for nine months, or six months after stability achieved	National Institute for Health Care and Excellence,^49^ American Family Physician^50^
Dementia		
Added	Counsel regarding medication use, side effects, and disease or treatment related misconceptions (if any)	National Institute for Health Care and Excellence,^53^ National Health Service,^54^ National Institute of Neurological Disorders and Stroke^28^^,^^55^
Added	Careful monitoring and prescription are recommended for benzodiazepines as cognition can worsen with its use	American Geriatric Society,^56^
Added	Cognitive Exercises can improve memory and problem solving and slow down cognitive decline (72). They are most effective when paired with physical exercise and healthy diet (73)	American Psychiatry Association,^57^ World Health Organization^58^ National Institute of Neurological Disorders and Stroke^55^
Delirium		
Added	Aim to rule out dementia on subsequent follow up	National Institute for Health Care and Excellence^59^
Added	Close monitoring is required as delirium can continue for weeks after medical issue is resolved	National Institute for Health Care and Excellence^59^
Added	Counsel regarding medication use, side effects, and disease or treatment related misconceptions (if any)	National Institute for Health Care and Excellence^59^
Adopt with Minor Changes	“Haloperidol is most frequently used because it has few anticholinergic side effects, few active metabolites, and a relatively small likelihood of causing sedation and hypotension. Haloperidol may be administered orally, intramuscularly, or intravenously and may cause fewer extrapyramidal symptoms when administered intravenously. Haloperidol can be initiated in the range of 1–2 mg every 2–4 h as needed (0.25–0.50 mg every 4 h as needed for elderly patients), with titration to higher doses for patients who continue to be agitated. For patients who require multiple bolus doses of antipsychotic medications, continuous intravenous infusions of antipsychotic medication may be useful (e.g., haloperidol bolus, 10 mg i.v., followed by continuous intravenous infusion of 5–10 mg/h; lower doses may be required for elderly patients). **For patients who require a more rapid onset of action, haloperidol can be considered followed by the newer antipsychotic medications (risperidone, olanzapine, and quetiapine) in the treatment of patients with delirium.** Patients receiving antipsychotic medications for delirium should have their ECGs monitored. A QTc interval greater than 450 msec or more than 25% over baseline may warrant a cardiology consultation and reduction or discontinuation of the antipsychotic medication. [I]”	Reason for change: Unavailability of droperidol in Pakistan
Eating disorders		
Added	Urgent referral is required for hypotension, bradycardia, arrythmias, hypoglycaemia, syncope, dehydration, refeeding syndrome, BMI<15 kg/m^2^	National Institute for Health Care and Excellence,^60^ American Family Physician^61^
Added	Counsel regarding medication use, side effects, and disease or treatment related misconceptions (if any)	National Institute for Health Care and Excellence,^60^ American Family Physician^61^
Added	Start fluoxetine when weight is stabilized to prevent relapse.	UpToDate^62^
Substance abuse		
Added	Counsel regarding medication use, side effects, and disease or treatment related misconceptions (if any)	World Health Organization,^63^ American Psychiatry Association,^64^ National Institute on Drug Abuse^65^

ASQ: Ask Suicide-Screening Questions, BSI: Beck Scale for Suicide Ideation, CBT: Cognitive behavioral therapy, ECT: Electroconvulsive therapy, ERP: Exposure and Response Prevention, MAOI: Monoamine oxidase inhibitor, OCD: Obsessive compulsive disorder, SNRI: Serotonin and norepinephrine reuptake inhibitors, SSRI: Selective serotonin reuptake inhibitor, TCA: Tricyclic antidepressant, TMS: Transcranial magnetic stimulation, VNS: Vagus nerve stimulation.

The challenges faced were broadly categorised into four main themes: resources, stakeholder support and involvement, resistance to change, and methodological limitations (Table 2).

**Table 2:** Challenges faced and proposed solutions.

Category of challenge	Specific challenge	Proposed solution
Resources	• Inadequate original data from Pakistan^b^	• Make use of regional literature • Judicious use of grey literature
	• Structuring the GRADE-ADOLOPMENT process to the resource-constrained context of Pakistan (revise through experience, highlight resource gap)^b^	• Conduct a thorough, realistic resource assessment, and highlight resource gaps • Revise process accordingly through experience
	• Inadequate expertise and experience with guideline development^a^	• Collaborate with personnel with requisite experience and expertise • Conduct comprehensive, standardised training modules for all personnel involved in the “adolopment” process
	• Inadequate manpower or size of workforce^a^	• Involve students and trainees on a volunteer basis
	• Suboptimal funding^a^	• Lobby for additional institutional and external funding
Stakeholder support and involvement	• Coordination between different stakeholders^b^	• Fixed, scheduled meetings with regular follow-ups with all stakeholders (awareness presentations or meeting in person)
	• Suboptimal provincial or federal government involvement^a^	• Involve all stakeholders from the start • Emphasise and reiterate mutual interests • Design specific curricula for all stakeholders involved • Tailor and deliver presentations to all stakeholders involved
	• Suboptimal involvement of external societies or organisations^a^	
	• Absence of patients' perspective^a^	
	• Absence of general practitioners' perspective^a^	
	• Absence of the allied health perspective^a^	
Resistance to change	• Experts' doubts regarding need for local EBCPGs^b^	• Initial presentation to emphasize need for local EBCPGs, robustness of the GRADE-ADOLOPMENT process, and the importance of strict adherence to rigorous GRADE-ADOLOPMENT processes to produce credible guidelines
	• Rigorousness of the GRADE-ADOLOPMENT process may discourage the process of adaptation (rigor ensures robustness)^b^	
	• Experts' doubts regarding nationwide implementation of local guidelines^a^	• Involve decision-makers from other institutions across the country and ensure buy-in to the newly created EBCPGs
Methodological limitations	• Individual-level biases from experts^b^	• Increase the number and diversity of experts • Gauge acceptability and accuracy of any revisions made by getting feedback from experts from external institutes
	• Group-level biases from experts^b^	
	• Suboptimal generalisability of consensus opinion based on five experts^b^	
	• Expert opinion is no substitute for the lack of scientific evidence^a^	• Supplement the expert opinion with as much auxiliary evidence as possible • Plan future studies to answer specific questions that arise during the GRADE-ADOLOPMENT process

a Minor challenge.

b Major challenge; EBCPG–evidence-based clinical practice guidelines.

## Discussion

We described our use of the GRADE-ADOLOPMENT process for creating local EBCPGs for the practice of psychiatry at the level of the GP in Pakistan. These EBCPGs were then framed into ten thorough, easy-to-follow primary care referral pathways for GPs. These referral pathways were designed to help GPs to easily understand and implement the EBCPGs and provide appropriate referrals to specialist mental health services when indicated.

Referral pathways bridge primary healthcare with secondary healthcare provided by specialists. These pathways serve as a vital means of communication and collaboration, ensuring that patients receive the most effective care possible. In particular, clinical referral pathways play a pivotal role in supporting GPs in fulfilling their critical role throughout the entire continuum of care. By starting with the screening and identification of potential psychiatric illnesses, these pathways enable GPs to refer patients to the appropriate specialists for further diagnosis, treatment, and management. A practical healthcare referral pathway for psychiatric conditions has several benefits: it can relieve the burden on scarce specialists, accelerate the provision of healthcare, improve the quality of clinical information in referrals, and reduce the number of unnecessary referrals.^66^ A study in New Zealand suggested that better integrated primary-to-secondary care pathways were needed to improve the quality of referrals and care provision.^67^ Another study by Wadoo and colleagues found that improvements in primary health care referrals at a mental health clinic in Qatar resulted in a 93% reduction in referrals with incomplete information, an 80% decrease in referrals that should have been directed elsewhere, and received positive feedback from the primary care physicians.^68^ Thus, the referral pathways we have created have the potential to bring about similarly positive changes.

In the context of LMICs, implementation of primary care management and referral pathways and EBCPGs could prove immensely beneficial. Greater than 85% of the world's population resides in LMICs, and greater than 80% of people suffering from mental disorders live in LMICs.^69^ In many LMICs, particularly in Africa and southeast Asia, there is a worrisome absence of mental health policies and laws to provide direction for their mental health programs and services.^70^ Additionally, a significant number of LMICs, including those in Africa, allocate less than 1% of their healthcare budget towards addressing mental health concerns.^71^ Hence, there is an urgent need to revisit existing mental health care systems to counter these alarming numbers. The implementation of EBCPGs is becoming increasingly recognised to improve the quality of healthcare.^72^ The integration of standardised EBCPGs has resulted in fewer discrepancies in the quality of patient care and better patient outcomes.^66^ Therefore, using standardised EBCPGs presents a promising tool to bring about a much-needed improvement in mental health care provision quality within LMICs such as Pakistan. Several countries have reported shorter treatment delays when patients first seek help from primary health care services instead of eliciting the help of traditional health practitioners.^73^ A study in Iran concluded that using GPs as the first contact resulted in a lower duration of untreated psychosis for patients with schizophrenia.^74^ Therefore, well-developed, and accessible primary care pathways can significantly improve measurable health parameters. The success of Colombia lends further support to incorporating standardised EBCPGs into LMICs. The EBCPGs proposed by Colombia in 1998 have now matured into reliable and high-quality (AGREE II) clinical standards implemented routinely nationwide.^75^ A standardised guideline model has been shown to provide a consistent framework for patient care, provide evidence-based recommendations, remove ambiguity, and offer clarity and uniformity in managing various mental health problems.^76^^,^^77^

To maximise the advantages of adopting the primary care referral pathways presented in this study, it is important to consider the types of mental health care provided at the primary care level. Because improvements in the quality of psychiatric referrals rely on the range of mental health care coverage found at the primary care level, increasing the range of mental health care at the base can directly improve access to care, the quantity and quality of referrals, the number of timely diagnoses made and the early initiation of treatments or therapies. Several high-income countries have begun incorporating cognitive behavioral therapy (CBT) and other forms of therapy in primary care.^78^ Therefore, to maximise benefit, the implementation of revised primary care pathways must be complemented by an expansion in the types of mental health care available at the primary care level. To aid this process, physicians should familiarise themselves with various manuals available.^79^ Provision of CBT at the primary care level will decrease treatment delay and prevent unnecessary referrals for conditions manageable by a GP.

Our study involved a thorough application of the GRADE-ADOLOPMENT process to introduce psychiatric EBCPGs in Pakistan. Table 1 summarises the recommendations which were either excluded or adopted with minor changes from the source guidelines and those that were added to the referral pathways. Several recommendations were excluded because of concerns pertaining to local availability and feasibility at the primary care level. General recommendations which were added for the management of psychiatric conditions in Pakistan included the discouragement of combining two selective serotonin reuptake inhibitors (SSRIs) or an SSRI with a serotonin and norepinephrine reuptake inhibitor (SNRI) because of a lack of evidence to support this augmentation.^20^ The recommendation to counsel patients on the delay in effects seen with SSRI drug therapy was also incorporated. This is important as many anti-depressants are slow to take effect, and the resulting window of therapeutic latency can be a cause for considerable frustration for patients and can also lead to issues of non-adherence to therapy.^80^

Furthermore, the use of the Triage Tool to assess a patient's potential to harm others or themselves and the Danger Assessment Tool to evaluate the patient's violent risk to healthcare personnel were added. The Ask Suicide-Screening Questions (ASQ) tools for assessment of suicide risk were also included from the National Institute for Occupational Safety and Health and the National Institute of Mental Health guidelines.^30^^,^^31^ In the primary care setting, the ASQ tool has been tested to have a sensitivity of 100% and a specificity of 87.9%. It was also found to be an effective tool in screening youth for risk of suicide.^81^ The Beck Scale for Suicide Ideation (BSI) is another Mental Health Triage (MHT) tool that may be used to assess the risk of suicide in psychiatric patients. BSI has a translated version in Urdu (national language of Pakistan) which may benefit in settings where questionnaires in English cannot be utilised.^29^ Overall, MHT tools in the primary care setting have been shown to cause an increase in patient satisfaction and accessibility to care and led to a rise in adequate and timely care responses for psychiatric patients.^82^

Going forward, we have several plans to ensure that the guidelines are effectively implemented and disseminated. We plan to incorporate the guidelines into the electronic health record system used in AKUH, which will ensure that the guidelines are easily accessible to healthcare professionals. Additionally, we plan to create and disseminate a “Manual of Therapeutics” that will contain all guidelines being created by the CCBP, which will serve as a comprehensive resource for healthcare professionals in our local setting. We also plan to develop a mobile application that will allow easy access to the guidelines and support remote usage and create a video lecture series delivered by local experts based on the guidelines. We aim to pilot these guidelines in our local setting and other hospitals and clinics. Furthermore, we plan to collaborate with external non-governmental organisations as well as government stakeholders to ensure that the guidelines are disseminated widely and that healthcare professionals are trained in their use. Additionally, to maximise the utility of EBCPGs it is necessary to also prepare GPs adequately for the proper implementation of these EBCPGs. Incentivised and institution-sanctioned training courses and workshops may help accustom GPs to the effective use of these guidelines within the Pakistani healthcare setting. This is important because GPs are able to screen patients en masse, and serve as first responders to psychiatric ailments, and operate as gatekeepers to appropriate specialist care. We believe that these efforts will ensure that the guidelines are effectively implemented and that patients receive the best possible care for their mental health conditions.

The transparency of the GRADE-ADOLOPMENT process will aid GPs in making a more informed decision, while the thorough step-by-step referral pathways will help streamline the referral process. The clear methodology will also aid researchers from other LMICs in developing a similar EBCPG and referral pathway. A limitation of the study is that our panel of experts comprised of four attending psychiatrists and the Head, Department of Psychiatry from the same hospital. A more diversified panel could have minimised individual or institutional biases that may have been involved in the findings.

## Conclusion

This study used the GRADE-ADOLPOMENT process to create ten EBCPGs for the management of psychiatric disorders in Pakistan. The recommendations added will help to bridge the gap in care provision and will help the physicians to treat patients holistically. Ten primary care management and referral pathways were also created for use by physicians for a smooth referral process. These psychiatric management and referral pathways, and EBCPGs will bring the healthcare system a step closer to achieving optimal health outcomes for patients.

## Contributors

The authors’ contributions were assessed using an objectively ranked score based on their involvement in guideline conceptualisation, guideline creation, manuscript conceptualisation, abstract, introduction, method, results write-up, data visualization (figures and tables), discussion, manuscript critical review, supervision, coordination, and management. TN, NA, RC, NAR, SN, MAM, and AHH were all involved in the conceptualisation process for the guideline. The creation of the guideline, which involved developing a methodology for the study and forming a panel of experts, was done by BA, TN, SSA, RC, NA, AAR, and NAR. AP contributed towards all aspects of the manuscript writing process and was involved in the supervision, coordination, and management of the manuscript development process. RSM, SP, MMB, MIB assisted in the write-up for various sections of the manuscript. All authors critically reviewed the article.

## Data sharing statement

All data generated or analysed during this study are included in this published article and its supplementary information files.

## Declaration of interests

The authors have no financial or personal conflict of interests to declare. We received no external funding for this research.
