# Supplementary material for: Adolopment of clinical practice guidelines and creation of referral pathways for psychiatric conditions in Pakistan
Source: Lancet Reg Health Southeast Asia. 2024 Mar 10;23:100387. doi: 10.1016/j.lansea.2024.100387 (PMC10938165; doi:10.1016/j.lansea.2024.100387)
Supplement: Supplementary Material [file mmc1.docx]

**Supplementary Material**

**Adolopment of clinical practice guidelines and creation of referral pathways for psychiatric conditions in Pakistan**

1. *Abbreviations*
2. *Supplementary Figure A: Process for Psychiatric Management EBCPG and Referral Pathway Creation*
3. *GRADE-ADOLOPMENT process for adaptation using Evidence to Decision (EtD) tables*
4. *Supplementary Table 1: Criteria evaluated in the Evidence to Decision (EtD) tables*
5. *Supplementary Table 2: Sample of Evidence to Decision (EtD) table*
6. *Supplementary Figure 1: Bipolar Disorder*
7. *Supplementary Figure 2: Obsessive Compulsive Disorder*
8. *Supplementary Figure 3: Schizophrenia*
9. *Supplementary Figure 4: Post-Traumatic Stress Disorder and Acute Stress Disorder*
10. *Supplementary Figure 5: Major Depressive Disorder*
11. *Supplementary Figure 6: Panic Disorder*
12. *Supplementary Figure 7: Delirium*
13. *Supplementary Figure 8: Dementia*
14. *Supplementary Figure 9a and 9b: Eating Disorders*
15. *Supplementary Figure 10: Substance Abuse*
16. *Supplementary Table 3: Key for Table of Recommendations*
17. *Section 1: Bipolar Disorder*
18. *Section 2: Obsessive Compulsive Disorder*
19. *Section 3: Schizophrenia*
20. *Section 4: Post-Traumatic Stress Disorder and Acute Stress Disorder*
21. *Section 5: Major Depressive Disorder*
22. *Section 6: Panic Disorder*
23. *Section 7: Delirium*
24. *Section 8: Dementia*
25. *Section 9: Eating Disorders*
26. *Section 10: Substance Abuse*

**Abbreviations**

- ABG: Arterial blood gas
- ADL: Activities of daily livings
- ADHD Attention deficient hyperactivity disorder
- AN: Anorexia nervosa
- ARFID-CBT: Cognitive behavioral therapy for avoidant/restrictive food intake disorder
- ASD: Acute stress disorder
- BED-CBT: Cognitive-behavioral therapy for binge-eating disorder
- BPD: Bipolar disorder
- BUN: Blood urea nitrogen
- CBC: Complete blood count
- CBT: Cognitive behavioral therapy
- CCBP: Centre for clinical best practices
- CDC: Centers for disease control and prevention
- CRP: C-reactive protein
- CT: Computed Tomography
- CXR: Chest X-ray
- DEXA: Dual-energy X-ray absorptiometry scan
- DLB: Dementia with lewy bodies
- DSM: Diagnostic and statistical manual of mental disorders
- EBCPG: Evidence based clinical practice guidelines
- ECG: Electrocardiography
- EEG: Electroencephalogram
- ECT: Electroconvulsive therapy
- EMDR: Eye movement desensitization and reprocessing
- ERP: Exposure & response prevention
- EtD: Evidence to decision table
- FDA: Food and drug administration
- FDG-PET: Fluorodeoxyglucose -Positron emission tomography
- GABA: γ-Aminobutyric acid
- HRT: Hormone replacement therapy
- ICD: International classification of diseases
- IDA: Iron deficiency anemia
- IPT: Interpersonal therapy
- MAOI: Monoamine oxidase inhibitor
- MET: Motivational enhancement therapy
- MDD: Major depressive disorder
- MoCA: Montreal cognitive assessment
- MRI: Magnetic resonance imaging
- NMDA: N-Methyl D-Aspartate
- NRT: Nicotine replacement therapy
- OCD: Obsessive compulsive disorder
- FPP: Planned focused psychodynamic psychotherapy
- PTSD: Post traumatic stress disorder
- RBS: Random blood sugar
- SGPT: Serum glutamic pyruvic
- SNRI: Serotonin and norepinephrine reuptake inhibitors
- SPECT: Single photon emission computed tomography
- SSRI: Selective serotonin reuptake inhibitors
- TCA: Tricyclic antidepressents
- TOR: Table of recommendations
- TSF: Twelve step facilitation
- TSH: Thyroid stimulating hormone
- VMAT2: Vesicular monoamine transporter 2
- Y-BOCS: Yale-brown obsessive-compulsive scale

**GRADEPro Evidence to Decision Framework**

**Supplementary Figure A: Process for Psychiatric Management EBCPG and Referral Pathway Creation**

**Selection of Source Guideline**

**Review all recommendations
●** Adopt, Exclude, Adapt

**Adopt in Current State/ with Minor Changes**

**Adaptation: Creation of GRADEPro Evidence to Decision (EtD) tables
●** Formulation of PICO Question **●** Best-Evidence Review
● Summary of Best-Evidence Review

**Exclude recommendations with reason of exclusion**

**Independent Expert Panel Review of EtD Tables**

**Combined Consensus Meeting between CCBP Staff and Expert Panel**

**Primary Care Referral Pathway Created**

**Completion of Review and Consensus on Final EBCPG**

**Primary care referral pathways drafted with local expert opinion**

**Additional recommendations sought by Best-Evidence Review if needed**

**Extraction of all recommendations unchanged into a Table of Recommendations.**

**GRADE-ADOLOPMENT process for adaptation using Evidence to Decision (EtD) tables**

GRADEPro is a web application used to create, manage, and share summaries of collected evidence.31 The CCBP staff involved in this study completed a training module to master the use of GRADEPro for the GRADE-ADOLOPMENT process. GRADEPro will be used to develop Evidence to Decision (EtD) tables to reach consensus on any recommendations marked as "Adapt".

EtD table is a framework that allows members of an expert panel to make health care recommendations or decisions based on compiled, balanced evidence. The development of EtD tables begins with the formulation of a question structured as follows: "Should the Intervention/Suggested Change be favored over the Comparison/Current Standard of Practice?" The merits and demerits of the proposed change are evaluated by a panel of experts based on 12 criteria, as shown in Supplementary Table 1.

**Best-evidence review**

A best-evidence review was performed to find research evidence against which the recommendation can be evaluated across the 12 criteria. The best-evidence assessment was performed separately for each of the 12 criteria and included a mini-systematic review and collection of supporting evidence.

- *Mini-systematic review*: A mini-systematic review follows the same general process as a full systematic review. However, it applies arbitrary selection criteria (such as publications’ geographical area) or a limit on the number of databases searched.^1^ In our mini-systematic review, PubMed and Google Scholar were queried using a search string generated using keywords from the recommendation in question. Only articles reporting data relevant to Pakistan were selected to maintain a local focus.

Two CCBP members independently screened titles and abstracts of articles sourced from PubMed and Google, and only articles that reported Pakistan-specific information were evaluated in full text for final inclusion. Since the source guide itself was created based on a systematic review process, a careful review of the entire text of the bibliography in the source document was also performed. The two CCBP members then extracted pertinent evidence from the final list of articles and summarized it in the EtD against the respective criteria.

- *Supporting evidence*: Information on the costs of various investigations and treatments, as well as the availability of diagnostic and management facilities was collected as needed from several local hospitals, facilities health, and pharmacy through telephone inquiries and websites.

**Expert panel review**

An expert panel of five senior attending psychiatrists will then be invited to review the completed EtD table for each recommendation and provide their input for each criterion in the form of a single selection from multiple response options. The experts will be instructed that if, for any criteria, they require additional evidence, they should inform the CCBP team. An effort will be made to source the requisite information, which, if found, will be shared with all the expert panel members. An unfilled GRADEPro EtD is shown as **Supplementary Table 2**.

**Final recommendation revisions and synthesis**

The CCBP staff will convene a meeting with the expert panel to review the anonymous EtD tables and reach a final consensus on the need and nature of any amendments to the proposed recommendations. The strength of each recommendation will also be decided. Finally, the consensus will be presented to the Head of Psychiatry, after which the recommendation will be included (in either original or revised form) or excluded in the local EBCPG depending on the nature of the consensus, along with a summary of the consensus decision.

| **Supplementary Table 1: Criteria evaluated in the Evidence to Decision (EtD) tables** | | |
| --- | --- | --- |
| **Criteria** | **Description** | **Interpretation** |
| **Problem** | The magnitude of a problem, as measured by its prevalence and severity in a local context | The more serious or urgent a problem is, the more likely that the option that better addresses the problem receives a strong recommendation. |
| **Desirable effects** | The magnitude of desirable effects is judged by considering the importance of the outcome and the size of the desirable effects (likelihood of experiencing a benefit or degree of benefits an individual experiences). | An option with greater desirable effects is more likely to gain a strong recommendation. |
| **Undesirable effects** | The magnitude of undesirable effects is similarly judged by considering the importance of the outcome and the size of the undesirable effects. | An option with fewer undesirable effects is more likely to gain a strong recommendation. |
| **Certainty of evidence** | Determined by likelihood that the research provides valid evidence regarding the effect of the option on all critical outcomes. | Evidence with higher certainty lends to a strong recommendation. |
| **Values** | Magnitude of value is judged by the variability or uncertainty of weightage placed upon the outcome by individuals. | Less variability/uncertainty of value leads to a strong recommendation. |
| **Balance of Effects** | The balance of effects is judged by considering the value individuals place upon the main outcomes, the degree of desirable and undesirable effects, and the certainty of those estimates. | The overall balance of effects can be judged as either favoring the intervention or comparison. |
| **Resources Required** | An estimate of the cost of the difference in resource use between the intervention and comparison. | An option with large savings is more likely to receive a strong recommendation. |
| **Certainty of Evidence of Required Resources** | It is determined by the likelihood that the research provides valid evidence of cost differences between the intervention and comparison. | Evidence with higher certainty lends to a strong recommendation. |
| **Cost-effectiveness** | Determines cost-effectiveness by considering uncertainty about or variability in costs or net benefit, sensitivity analyses, and the reliability and applicability of the economic evaluation. | An option that is more cost-effective is more likely to receive a strong recommendation |
| **Equity** | Likelihood of differences in the relative effectiveness of the intervention for disadvantaged subgroups that influence the absolute effectiveness of the intervention. | An option with a greater likelihood to favor equity is more likely to receive a strong recommendation. |
| **Acceptability** | Likelihood of key stakeholders to accept the distribution of benefits, harms, costs, and ethical concerns associated with the intervention, over an extended period. | An option more acceptable to most stakeholders is more likely to receive a strong recommendation. |
| **Feasibility** | Practicality of sustained use of the intervention. | An option more feasible to most stakeholders is more likely to receive a strong recommendation. |

| **Supplementary Table 2: Sample Evidence to Decision (EtD) table** | | | |
| --- | --- | --- | --- |
| **Question**: Should *Intervention/Suggested Change* be favored over *Comparison/Current Standard of Practice*? | | | |
| **Criteria** | **Research Evidence** | **Additional Considerations** | **Judgment** |
| **Problem:** Is the problem a priority? |  |  | - No - Probably No - Probably Yes - Yes - Varies - Don’t Know |
| **Desirable effects:** How substantial are the desirable anticipated effects? |  |  | - Trivial - Small - Moderate - Large - Varies - Don’t Know |
| **Undesirable effects**: How substantial are the undesirable anticipated effects? |  |  | - Large - Moderate - Small - Trivial - Varies - Don’t Know |
| **Certainty of evidence**: What is the overall certainty of the evidence of effects? |  |  | - Very Low - Low - Moderate - High - No Included Studies |
| **Value**: Is there important uncertainty about or variability in how much people value the main outcomes? |  |  | - Important Uncertainty or Variability - Possible Uncertainty or Variability - Probably No Important Uncertainty or Variability - No Important Variability or Uncertainty |
| **Balance of effects**: Does the balance between desirable and undesirable effects favor the intervention or the comparison? |  |  | - Favors Comparison - Probably Favors the Comparison - Does Not Favor Either the Intervention or Comparison - Probably Favors the Intervention - Favors Intervention - Varies - Don’t Know |
| **Resources eequired**: How large are the resource requirements (costs)? |  |  | - Large Costs - Moderate Costs - Negligible Costs or Savings - Moderate Savings - Large Savings - Varies - Don’t Know |
| **Certainty of evidence of required resources**: What is the certainty of the evidence of resource requirements (costs)? |  |  | - Very Low - Low - Moderate - High - No Included Studies |
| **Cost-effectiveness**: Does the cost-effectiveness of the intervention favor the intervention or the comparison? |  |  | - Favors Comparison - Probably Favors the Comparison - Does Not Favor Either the Intervention or Comparison - Probably Favors the Intervention - Favors Intervention - Varies - No Included Studies |
| **Equity**: What would be the impact on health equity? |  |  | - Reduced - Probably Reduced - Probably No Impact - Probably Increased - Increased - Varies - Don’t Know |
| **Acceptability**: Is the intervention acceptable to key stakeholders? |  |  | - No - Probably No - Probably Yes - Yes - Varies - Don’t Know |
| **Feasibility**: Is the intervention feasible to implement? |  |  | - No - Probably No - Probably Yes - Yes - Varies - Don’t Know |
| **Overall Recommendations**   - Strong Recommendation Against the Intervention - Conditional Recommendation Against the Intervention - Conditional Recommendation for Either the Intervention or the Comparison - Conditional Recommendation for the Intervention - Strong Recommendation for Intervention | | | |


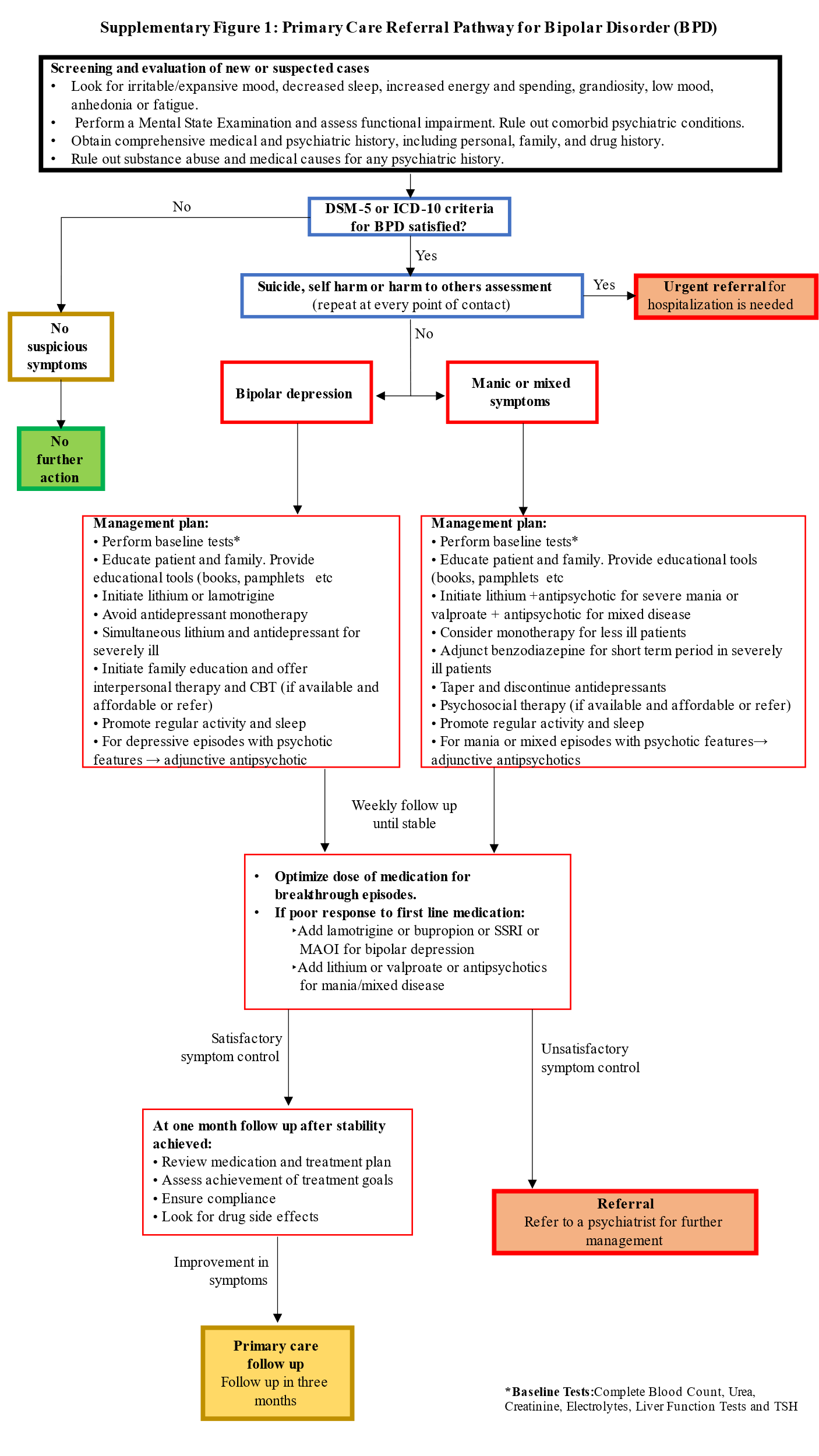


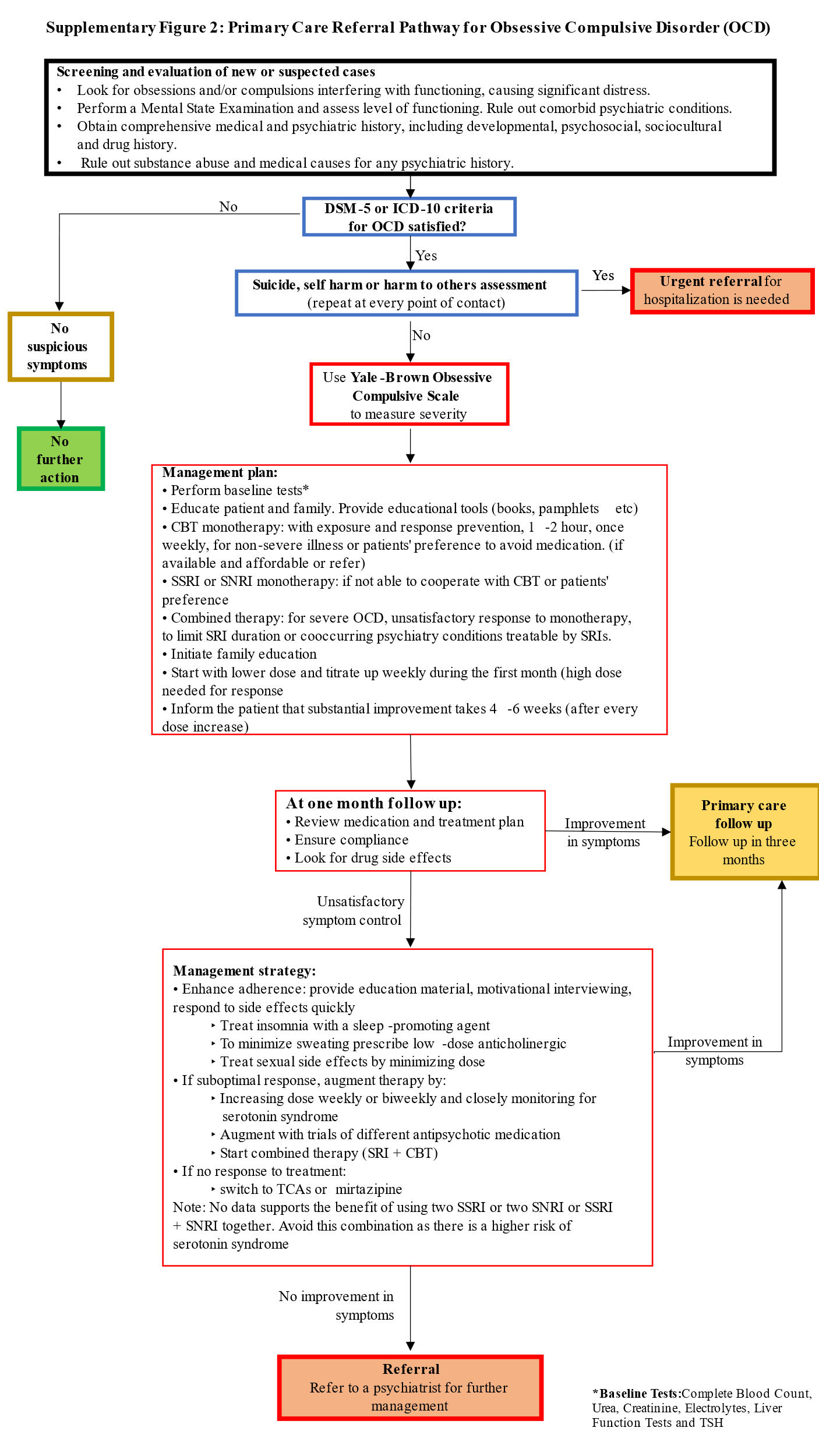


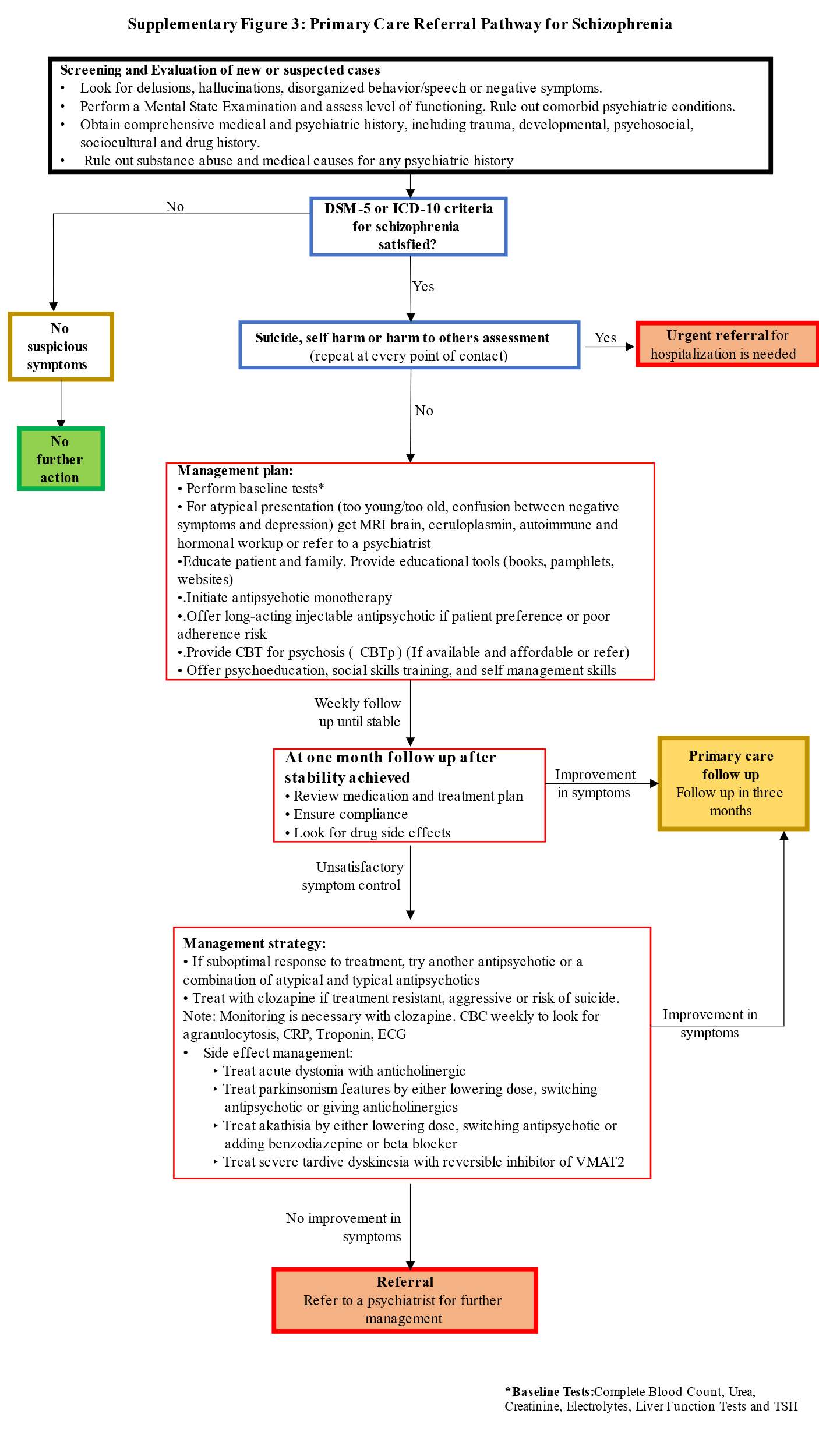


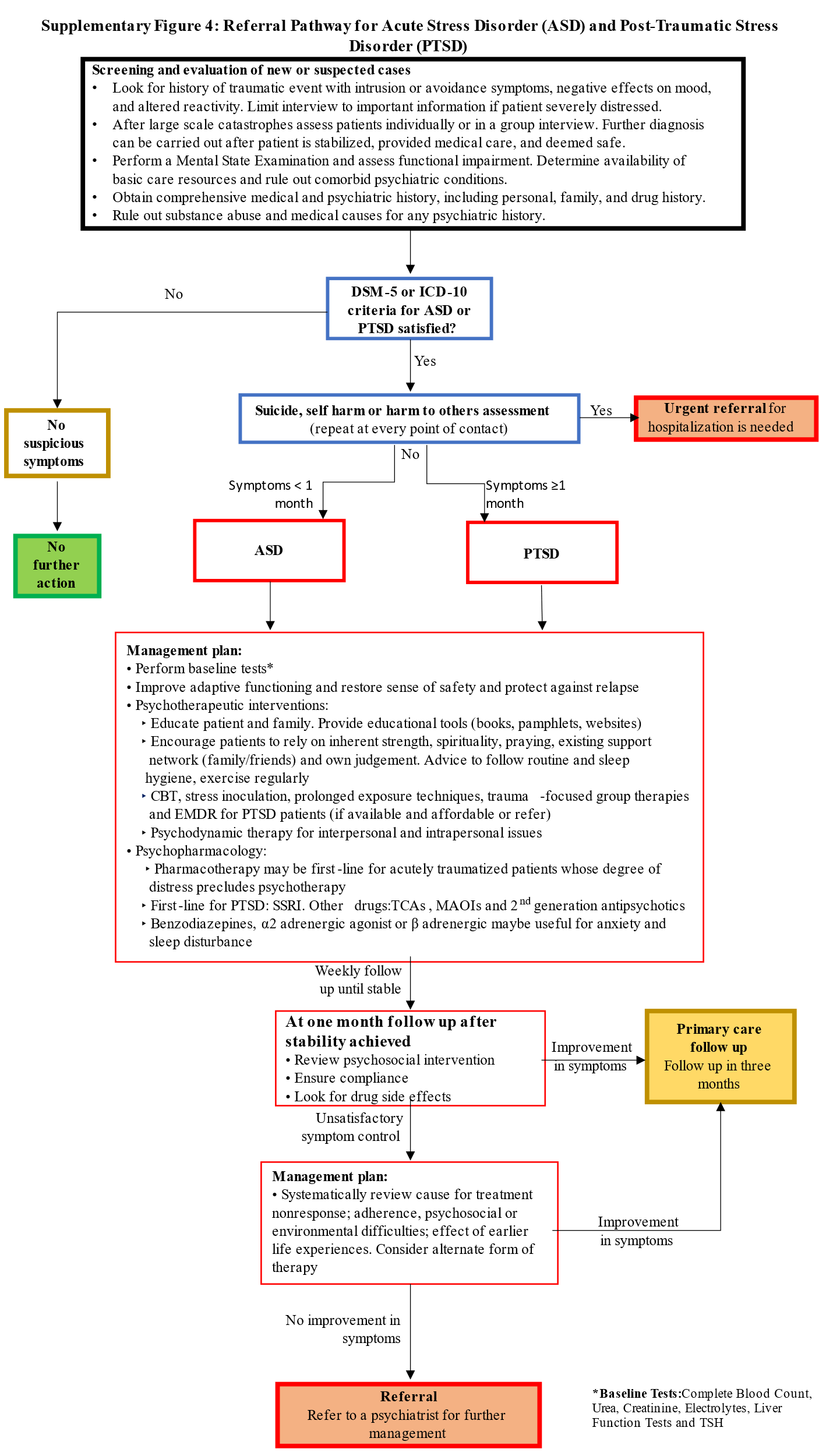


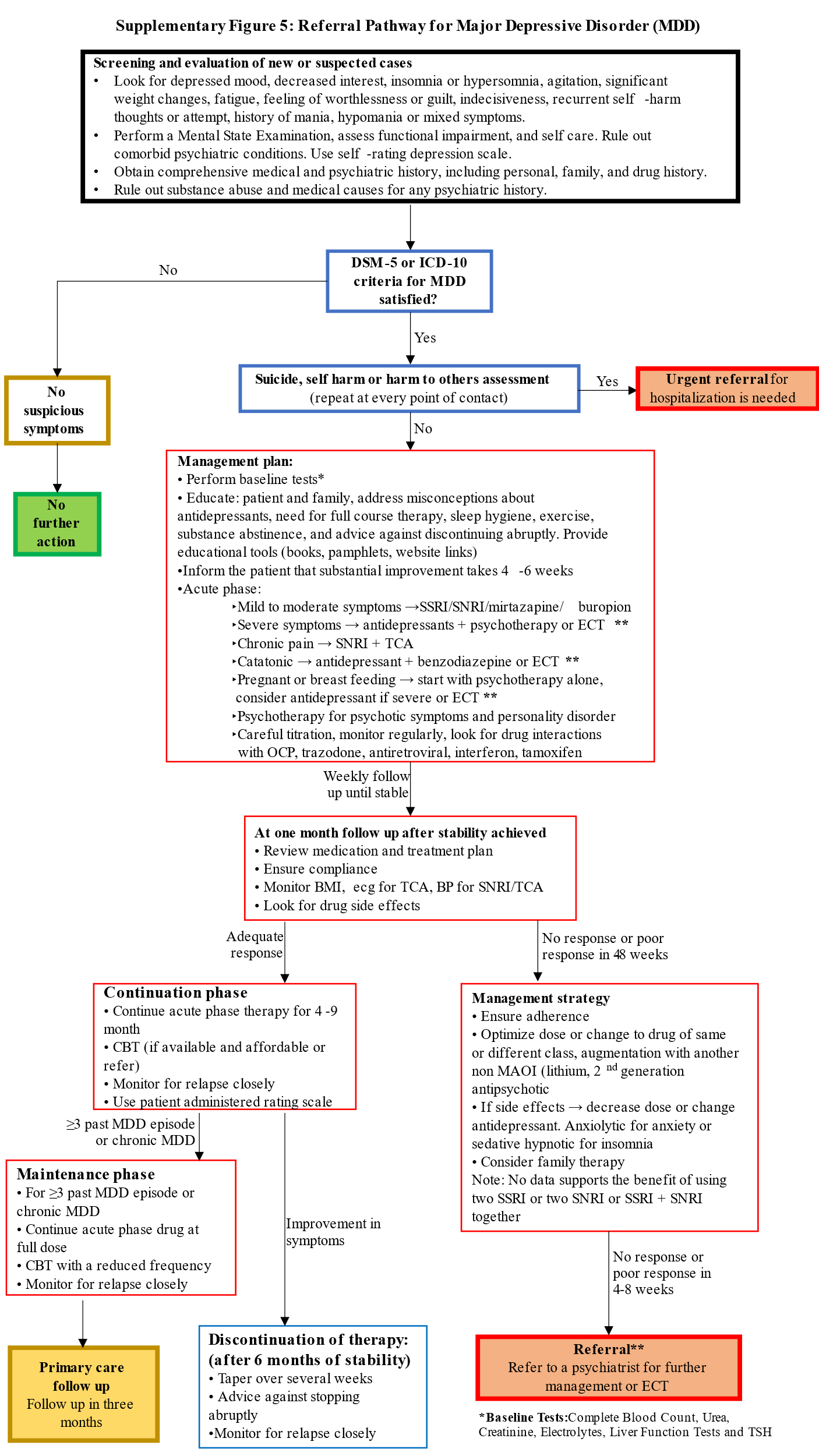


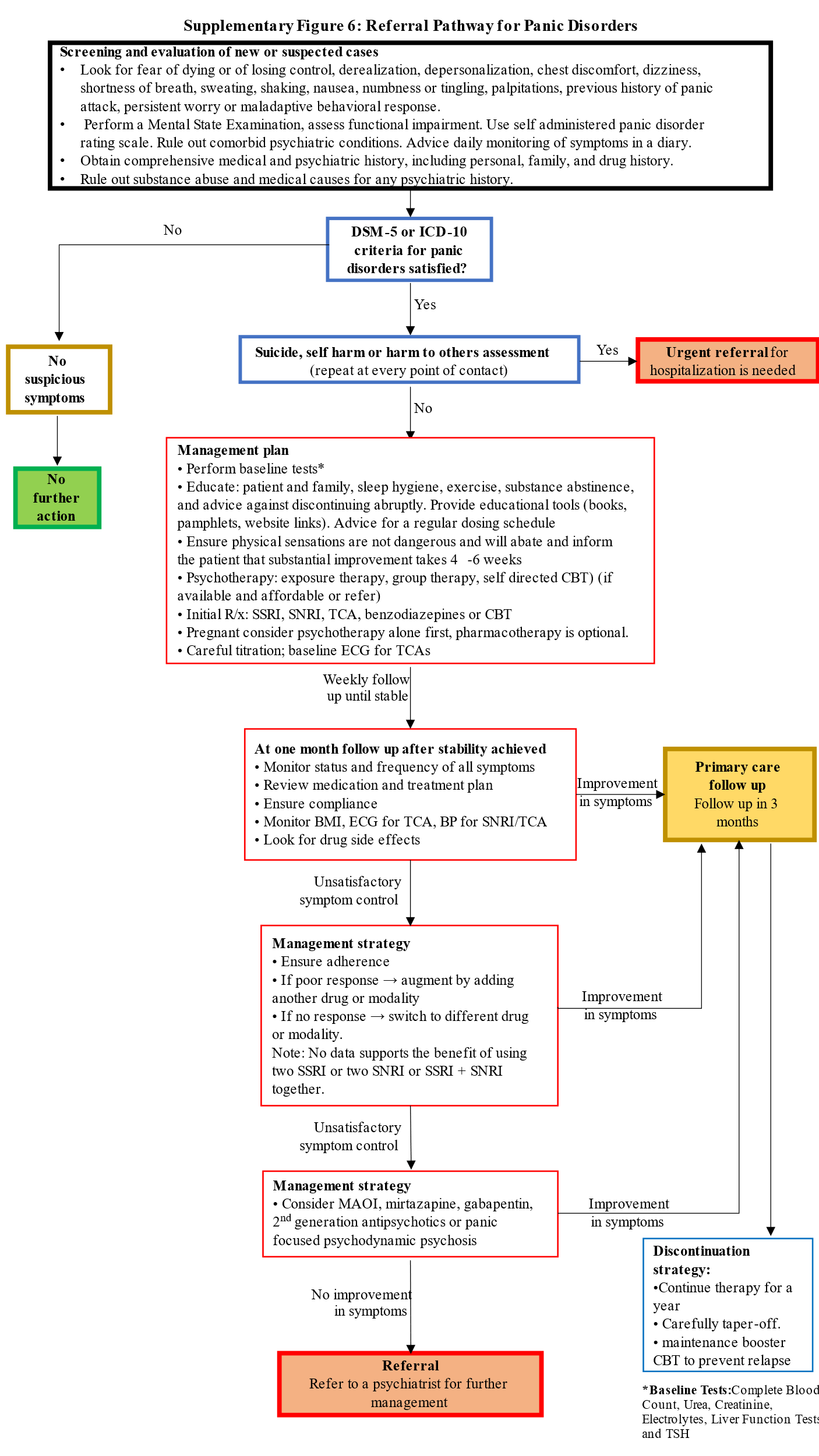


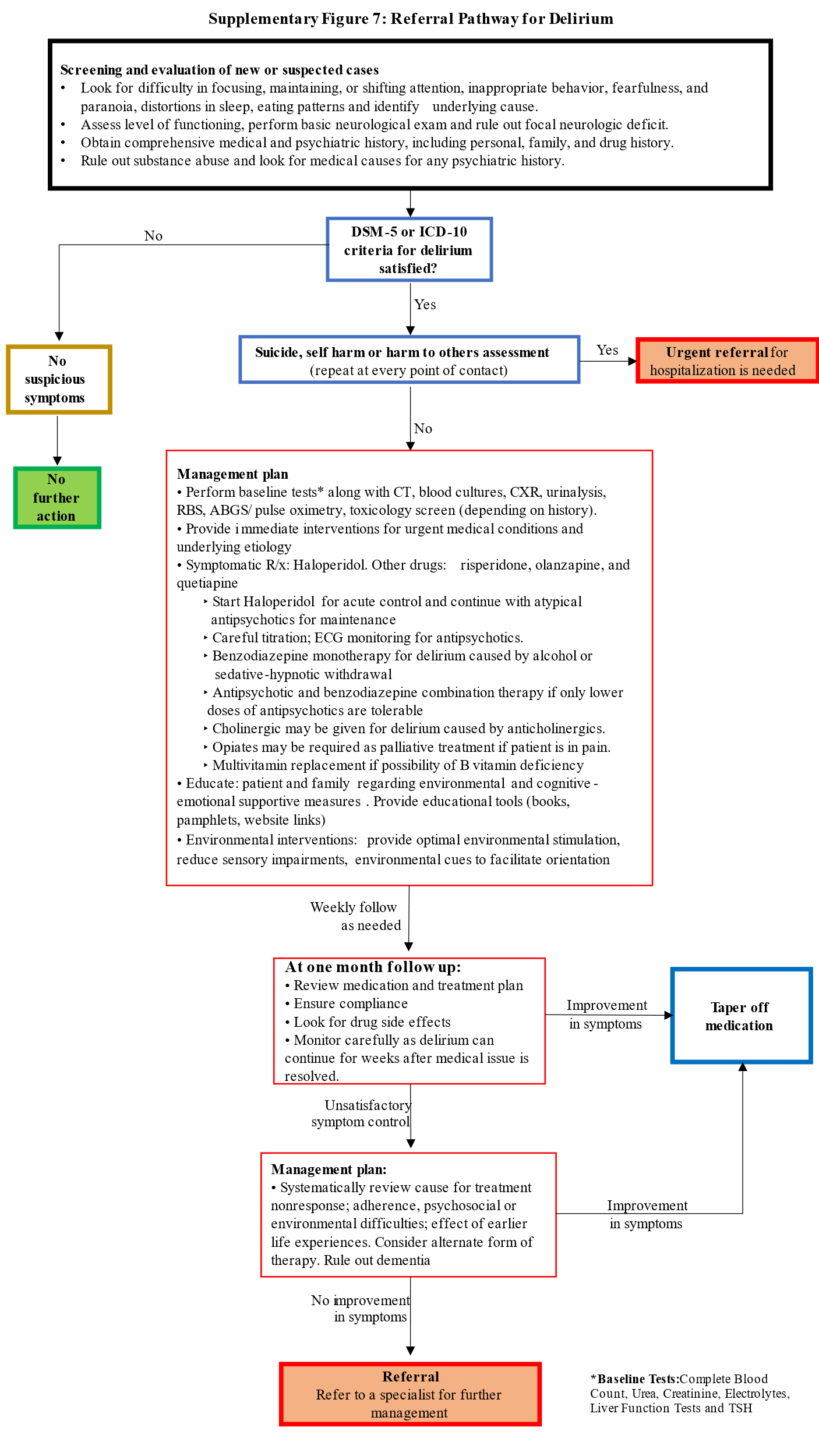


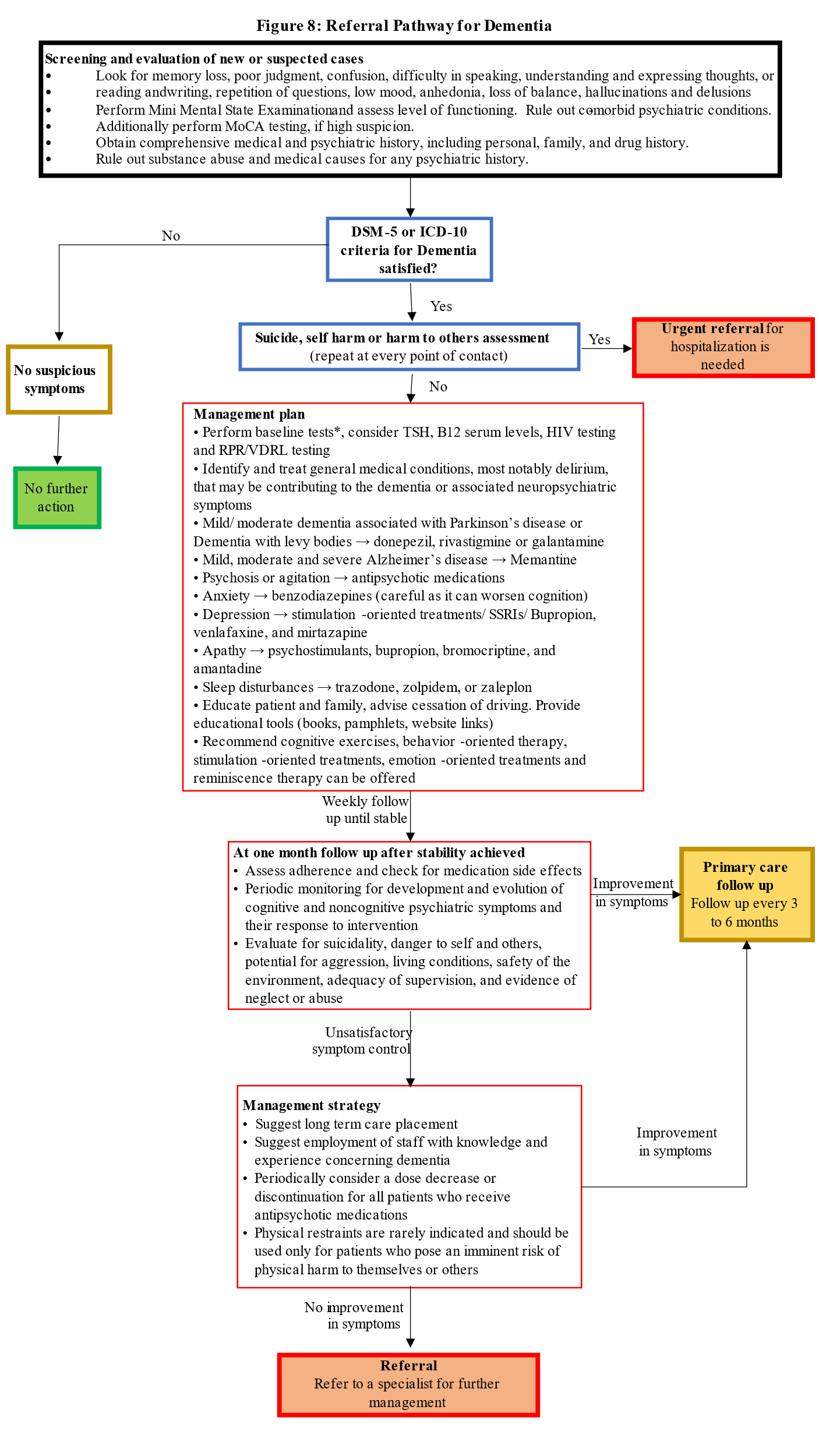


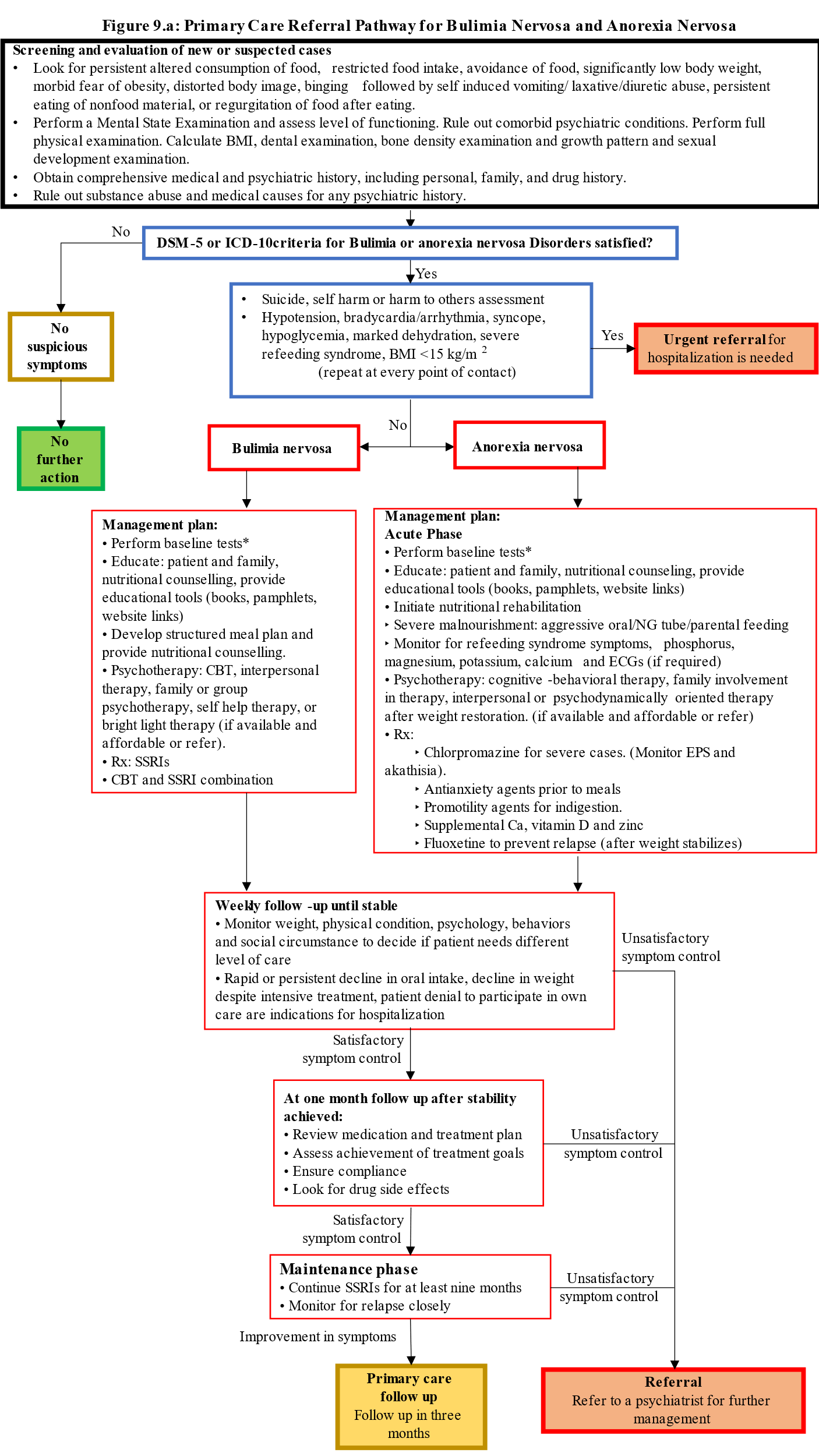


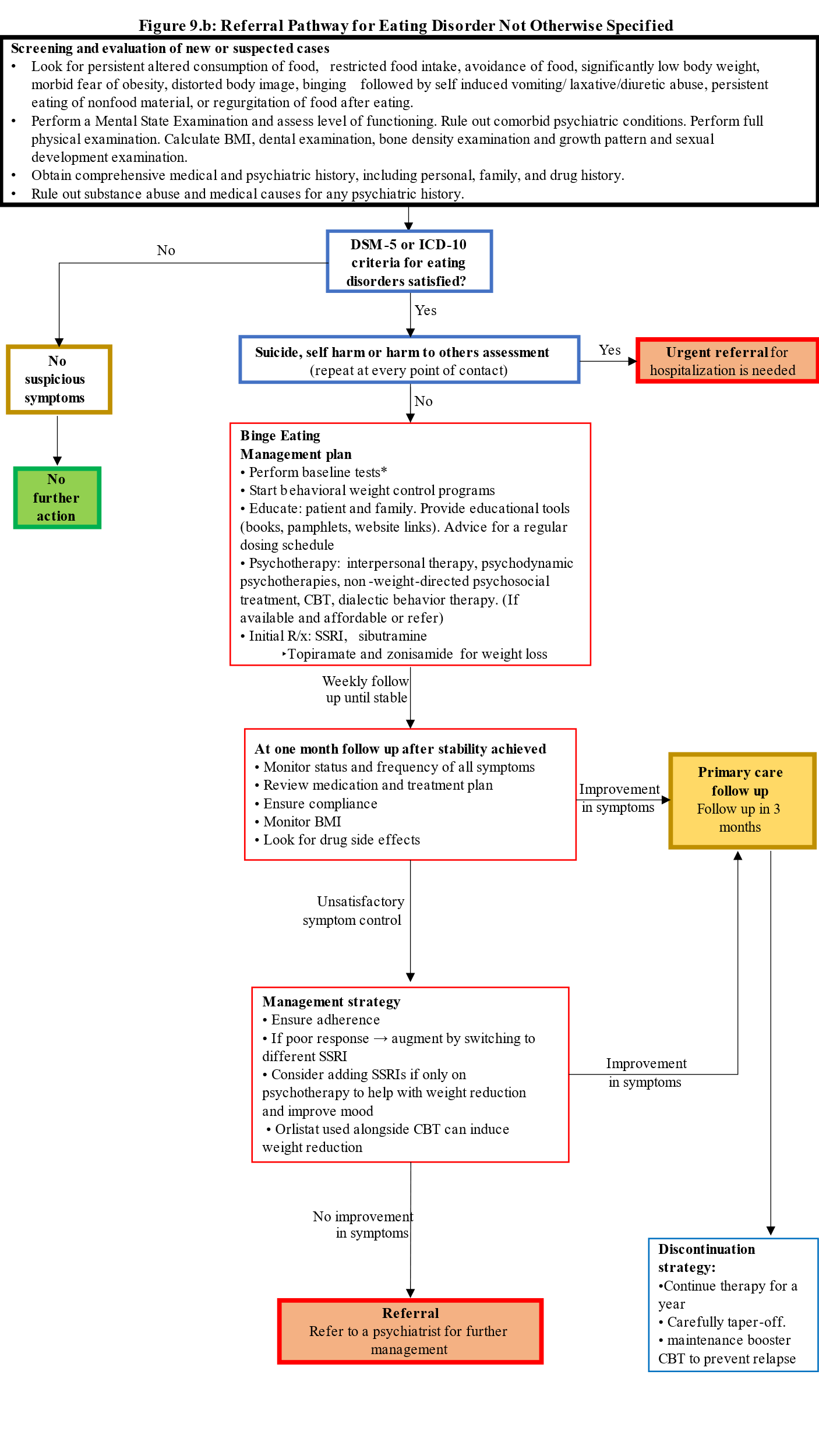


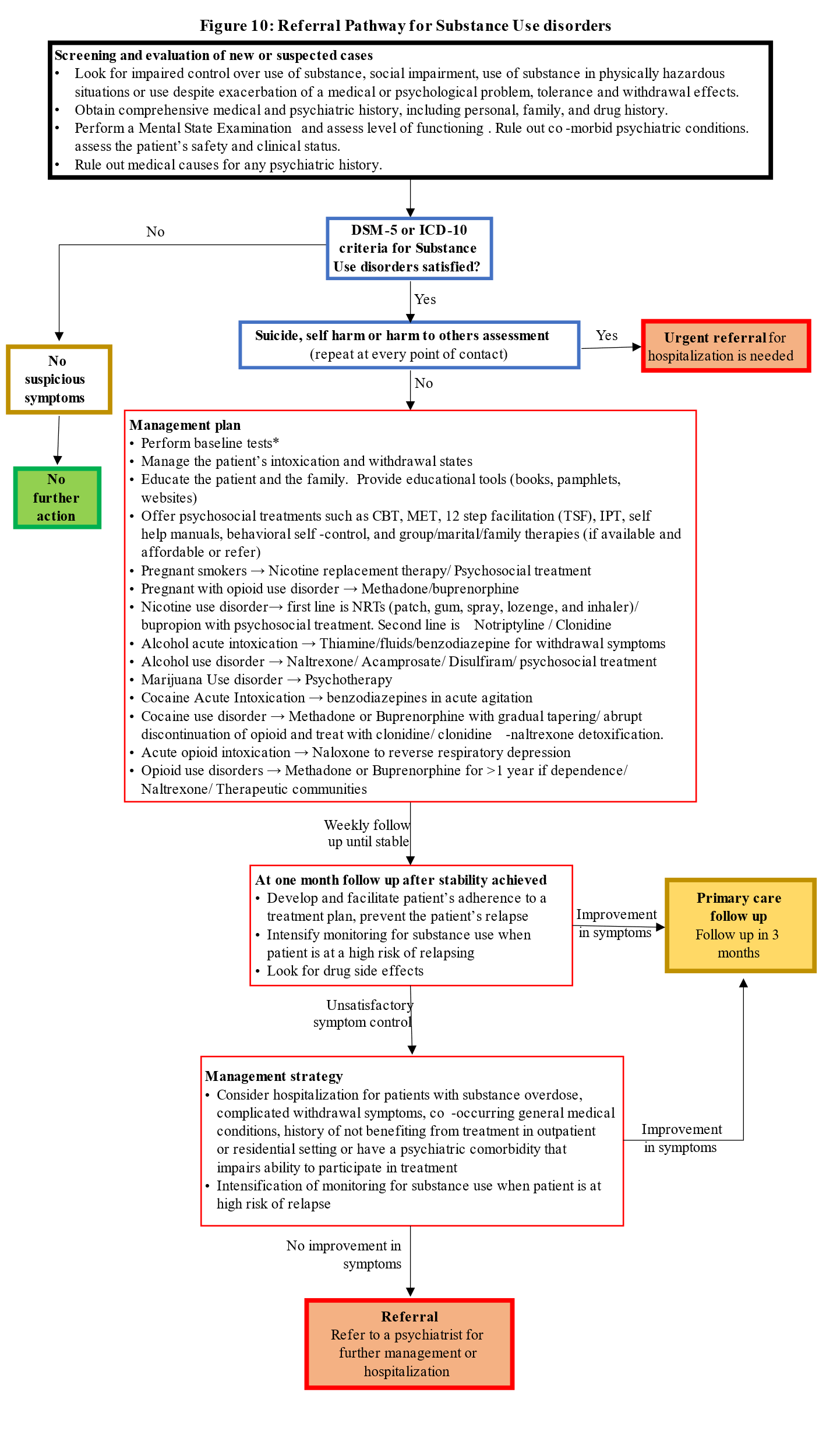


| **Supplementary Table 3: Key for table of recommendations** | |
| --- | --- |
| **Adopt** | The primary care physician should adopt the recommendation as is |
| **Refer** | The primary care physician should refer the patient to a specialist at this point |
| **Adopt/ Refer** | The primary care physician should provide the service if available in the primary care set-up, if the service is not available the patient should be referred to a specialist |

**SECTION 1: BIPOLAR DISORDER**

**The Treatment of Patients with Bipolar Disorder**

**Source Guideline**: Practice guideline for the treatment of patients with bipolar disorder (revision).
American Psychiatric Association. 2002.^2^

**Key to understanding level of evidence and strength of recommendation**

| Grade of Recommendation | |
| --- | --- |
| [I] | Recommended with substantial clinical confidence |
| [II] | Recommended with moderate clinical confidence |
| [III] | May be recommended based on individual circumstances |

**Table of Recommendations**

|  | **Psychiatric management** |
| --- | --- |
| **Adopt** | Morbidity and mortality can be **decreased** with treatment.  [I] |
| **Adopt** | Perform a **diagnostic evaluation** and assess **patient’s safety** and level of functioning to arrive at a decision  about optimum treatment setting.  [I] |
| **Adopt** | Establish and maintain a **therapeutic alliance**, monitor patient’s psychiatric status, provide education  regarding bipolar disorder, enhance treatment compliance, promote regular patterns of activity and sleep,  anticipate stressors, identify new episodes early and minimize functional impairments. [I] |
|  | **Acute Treatment** |
|  | **Manic or mixed episodes** |
| **Adopt** | The **first-line pharmacological treatment** for more severe manic or mixed episodes is initiation of either  **lithium** plus an **antipsychotic** or **valproate** plus an **antipsychotic**.  [I] |
| **Adopt** | Monotherapy with lithium, valproate, or an antipsychotic such as olanzapine may be sufficient for less ill  patients. [I] |
| **Adopt** | **Adjunctive treatment** with a **benzodiazepine** for short-term.  [II] |
| **Adopt** | **Prefer valproate over lithium** for mixed episodes.  [II] |
| **Adopt** | Treat with **atypical antipsychotics** because of their more **benign side effect** profile.  [I] |
| **Adopt** | Use olanzapine or risperidone.  [II] |
| **Adopt** | Alternatives include carbamazepine or oxcarbazepine in lieu of lithium or valproate. [II] |
| **Adopt** | **Taper and discontinue antidepressants** if possible.  [I] |
| **Adopt** | Combine **psychosocial therapy** with pharmacotherapy.  [I] |
| **Adopt** | **Optimize the medication dose** in patients who, despite receiving maintenance medication treatment,  experience a manic or mixed episode (i.e., a “breakthrough” episode). [I] |
| **Adopt** | Introduce or resume an antipsychotic.  [II] |
| **Adopt** | Treat severely ill or **agitated patients** requiring short-term adjunctive treatment with a **benzodiazepine.**  [I] |
| **Adopt** | After failure of first-line medication at optimal doses to control symptoms, consider addition of another  first-line medication.  [I] |
| **Adopt** | Include carbamazepine or oxcarbazepine in lieu of an additional first line medication [II], adding an  antipsychotic if not already prescribed.  [I]  Or changing from one antipsychotic to another.  [III] |
| **Adopt** | **Clozapine** may be effective in treatment of **refractory illness**  [II] |
| **Refer** | Consider **Electroconvulsive therapy (ECT)** for patients with severe or treatment-resistant mania or if  preferred by patient in consultation with psychiatrist. [I] |
| **Refer** | **ECT** is a potential treatment for patients experiencing mixed episodes or for patients experiencing severe  mania during **pregnancy.**  [II] |
| **Adopt** | **Manic or mixed episodes** with psychotic features require treatment with an **antipsychotic medication**.  [II] |
|  | **Depressive episodes** |
| **Adopt** | Initiate treatment with **lithium**  [I] or  **lamotrigine**  [II] |
| **Adopt** | **Avoid antidepressant monotherapy**.  [I] |
| **Adopt** | Initiate **simultaneous treatment** with lithium and an antidepressant for more severely ill patients.  [III] |
| **Refer** | Offer **ECT** to patients with **life-threatening inanition, suicidality, or psychosis**.  [I] |
| **Refer** | **ECT** is also a potential treatment for severe depression during **pregnancy**.  [II] |
| **Adopt/**  **Refer** | Offer **psychotherapy** in treatment of **unipolar depression**.  [I] |
| **Adopt/**  **Refer** | In **bipolar depression**, use **interpersonal therapy** and **cognitive behavior therapy** together with  pharmacotherapy.  [II]  Psychodynamic psychotherapy is used in addition to medication.  [III] |
| **Adopt** | **Optimize the dose** of maintenance medication for patients who suffer a breakthrough depressive episode.  [II] |
| **Adopt** | When an acute depressive episode of bipolar disorder **does not respond to first-line** medication treatment  at optimal doses, add **lamotrigine.** [I] |
| **Adopt** | **bupropion**  [II] |
| **Adopt** | or **paroxetine**  [II] |
| **Adopt** | add other **newer antidepressants** (e.g., a selective serotonin reuptake inhibitor [SSRI] or venlafaxine) [II] |
| **Adopt** | or a monoamine oxidase inhibitor (MAOI)  [II] |
| **Refer** | Consider **ECT** for patients with **severe or treatment-resistant depression** or depression with **psychotic or**  **catatonic features.**  [I] |
| **Adopt** | Offer use of **antidepressant treatment earlier** in patients with **bipolar II disorder**.  [II] |
| **Adopt** | Provide **adjunctive treatment** with an antipsychotic medication in **depressive episodes** with psychotic.  [I] |
| **Refer** | Offer ECT as alternative.  [I] |
|  | **Rapid cycling** |
| **Adopt** | The initial intervention in patients who experience rapid cycling is to identify and treat **medical conditions**,  such as hypothyroidism or drug or alcohol use, that may contribute to cycling. [I] |
| **Adopt** | **Taper** certain medications, particularly antidepressants.  [II] |
| **Adopt** | Provide **initial treatment** with **lithium or valproate**.  [I] |
| **Adopt** | Alternate treatment with **lamotrigine.** [I] |
| **Adopt** | Use combinations of medications.  [II] |
|  | **Maintenance Treatment** |
| **Adopt** | Offer maintenance regimens of medication following a **manic episode.**  [I] |
| **Adopt** | Consider maintenance treatment. [II] |
| **Adopt** | Treat with **lithium**  [I]  and **valproate**  [I] |
| **Adopt** | Alternatives include **lamotrigine**. [II] |
| **Adopt** | **Carbamazepine or oxcarbazepine**.  [II] |
| **Adopt** | If one of these medications was used to achieve remission from the most recent depressive or manic episode,  then continue same treatment.  [I] |
| **Refer** | Consider maintenance sessions of ECT for patients whose acute episode responded to ECT. [II] |
| **Adopt** | **Reassess the need** for ongoing antipsychotic treatment for patients treated with an antipsychotic medication  during the preceding acute episode, upon entering maintenance treatment.  [I] |
| **Adopt** | **Discontinue antipsychotics** **unless** they are required for control of **persistent psychosis.** [I] |
| **Adopt** | or **prophylaxis against recurrence.** [III] |
| **Adopt** | Consider **maintenance therapy with atypical antipsychotics**.  [III] |
| **Adopt/**  **Refer** | During maintenance treatment, patients are likely to benefit from a **concomitant psychosocial intervention**  —including **psychotherapy**—that addresses illness management (i.e., adherence, lifestyle changes and early  detection of prodromal symptoms) and interpersonal difficulties. [II] |
| **Refer** | **Group psychotherapy** may help patients address such issues as **adherence** to treatment plan, **adaptation** to  chronic illness, **regulation** of self-esteem and management of marital and other psychosocial issues.  [II] |
| **Refer** | Support groups provide useful information about bipolar disorder and its treatment.  [I] |
| **Adopt** | Patients who continue to experience **subthreshold symptoms or breakthrough mood episodes** may require  the **addition** of another maintenance medication.  [II]  an **atypical antipsychotic**  [III] |
| **Adopt** | or an **antidepressant**  [III] |
| **Refer** | Consider **maintenance sessions of ECT** for patients whose acute episode responded to ECT.  [II] |

**ACRONYMS AND ABBREVIATIONS**

| BUN: Blood urea nitrogen | ECT: Electro convulsive therapy |
| --- | --- |
| CBC: Complete blood count | SGPT: Serum glutamic pyruvic |
| Cr: Creatinine | TSH: Thyroid stimulating hormone |

**SECTION 2: OBSESSIVE COMPULSIVE DISORDER MANAGEMENT**

**Treatment of patients with obsessive-compulsive disorder
Source Guideline**: Practice Guideline for the treatment of patients with obsessive-compulsive disorder
American Psychiatric Association. 2007.^3^

**Key to understanding level of evidence and strength of recommendation**

| Grade of Recommendation | |
| --- | --- |
| [I] | Recommended with substantial clinical confidence |
| [II] | Recommended with moderate clinical confidence |
| [III] | May be recommended on the basis of individual circumstances |

**Table of Recommendations**

|  | **Psychiatric management** |
| --- | --- |
| **Adopt** | Obsessive-compulsive disorder (OCD) seen in clinical practice is usually a **chronic** illness with a  **waxing and waning** course. Treatment is indicated when OCD symptoms interfere with **functioning**  or cause significant distress.  [I] |
| **Adopt** | Psychiatric management consists of an array of therapeutic actions that may be offered to all patients  with OCD during the course of their illness at an intensity consistent with the individual patient’s  **needs, capacities, and desires**.  [I] |
| **Adopt** | It is important to **coordinate** the patient’s care with physicians treating co-occurring medical  conditions, other clinicians, and social agencies such as schools and vocational rehabilitation programs.  [I] |
| **Adopt** | OCD patients who are parents of young children may want advice regarding the **genetic risk** of OCD.  It is important for clinicians to explain to such patients that the available data indicate an **increased**  **but modest risk** of OCD in the children of affected individuals; patients wanting more information  may be referred to a genetic counselor.  [I] |
|  | **Establishing a Therapeutic Alliance** |
| **Adopt** | Establishing and maintaining a **strong therapeutic alliance** is important so that treatment may be  jointly, and therefore more effectively, planned and implemented.  [I] |
| **Adopt** | Steps toward this end include tailoring one’s communication style to the **patient’s needs and**  **capacities**, explaining symptoms in **understandable** terms, and being both **encouraging** and  **comforting.**  [I] |
| **Adopt** | The **excessive doubting** that is characteristic of OCD may require special approaches to building  the alliance, including allowing the patient **extra time** to consider treatment decisions and **repeating**  **explanations** (a limited number of times).  [I] |
| **Adopt** | In building the therapeutic alliance, the psychiatrist should also consider how the **patient feels and**  **acts** toward him or her as well as what the **patient wants and expects** from treatment.  [I] |
|  | **Assessing the Patient’s Symptoms** |
| **Adopt** | In assessing the patient’s symptoms with the aim of establishing a diagnosis using DSM-IV-TR  criteria, it is important to differentiate the obsessions, compulsions, and rituals of OCD from **similar**  **symptoms** found in **other disorders**, including depressive ruminations, the worries of **generalized**  **anxiety disorder**, the intrusive thoughts, and images of **posttraumatic stress disorder**, and  **schizophrenic,** and **manic** delusions.  [I] |
|  | **Using Rating Scales** |
| **Adopt** | The psychiatrist should consider **rating the baseline severity** of OCD symptoms and co-occurring  conditions and their effects on the patient’s functioning, using a scale such as the **10-item Yale-Brown**  **Obsessive Compulsive Scale (Y-BOCS),** since this provides a way to **measure response** to treatment.  [I] |
| **Adopt** | If a rating scale is not used, it is helpful to document the patient’s **estimate of the number of hours**  **per day** spent obsessing and performing compulsive behaviors, and the **degree of effort** applied to  trying to escape the obsessions and to resisting the behaviors.  [I] |
| **Adopt** | Recording actively avoided items or situations also provides a **useful baseline** against which change  can be measured.  [I]  Scales may also be utilized to rate other symptoms, such as **depression or degree of disability** |
|  | **Enhancing the Safety of the Patient and Others** |
| **Adopt** | The psychiatrist should **evaluate** the **safety** of the patient and others  [I]  This entails assessing the patient’s **potential for self-injury or suicide**, since individuals with OCD  alone or with a lifetime history of any co-occurring disorder have a higher suicide attempt rate than do  individuals in the general population. Although acting on aggressive impulses or thoughts has not been  reported in OCD, and patients **rarely resort to violence** when others interfere with their performing  their compulsive rituals, it remains important to inquire about past aggressive behavior. OCD patients  who fear loss of control may engage in extensive **avoidance rituals** in an effort to contain their  symptoms. |
| **Adopt** | The psychiatrist should understand that individuals with OCD are not immune to **co-occurring**  **disorders** that may increase the likelihood of suicidal or aggressive behavior. When such co-occurring  conditions are present, it is important to arrange treatments that will **enhance the safety** of the patient  and others.  [I] |
|  | **Completing the Psychiatric Assessment** |
| **Adopt** | In completing the psychiatric assessment, the psychiatrist will usually consider **all the elements** of  the traditional medical evaluation.  [I] |
| **Adopt** | With regard to co-occurring conditions, the psychiatrist should pay particular attention to past or  current **evidence of depression**, given its frequency and association with suicidal ideation and  behaviors.  [I] |
| **Adopt** | Exploration for **co-occurring bipolar disorder** and family history of bipolar disorder is also important  in view of the risk of **precipitating hypomania or mania** with anti-OCD medications.  [I] |
| **Adopt** | Other **anxiety disorders** are common in OCD patients, as are **tic disorders**, and may complicate  treatment planning. Other disorders that may be more common and may complicate treatment  planning include **impulse-control** disorders, **anorexia nervosa**, **bulimia nervosa, alcohol use**  **disorders, and attention-deficit/ hyperactivity disorder**. Past histories of panic attacks, mood  swings, and substance abuse or dependence are also relevant.  [I] |
| **Adopt** | It is important to document the patient’s **course of symptoms and treatment history**, including  psychiatric hospitalizations and trials of medications (with details on treatment adequacy, dose,  duration, response, and side effects) and psychotherapies (with details on the nature, extent, and  response to all trials).  [I] |
| **Adopt** | The psychiatrist should also assess the patient’s **developmental, psychosocial, and sociocultural**  **history**, including his or her primary support group and sociocultural supports, potential psychosocial  stressors, educational and occupational history (including military history), sexual history, and capacity  to navigate developmental transitions and achieve stable and gratifying familial and social relationships.  [I] |
| **Adopt** | In addition, the psychiatrist should evaluate **how OCD has interfered** with academic and vocational  achievement as well as familial, social, and sexual relationships [I]. Having evaluated the symptoms  and their effects on well-being, functioning, and quality of life, the psychiatrist should assess the role  of the patient’s social supports in facilitating treatment and in maintaining or exacerbating symptoms.  [I] |
| **Adopt** | The psychiatrist should consider whether the OCD is a **manifestation of a general medical condition**  [I]; document current medical conditions, relevant hospitalizations, and any history of head trauma,  loss of consciousness, or seizures.  [I] |
| **Adopt** | Record the presence and severity of **somatic or psychological symptoms** that could be confused with  medication side effects.  [I] |
| **Adopt** | Current medications and doses, including hormonal therapies, herbal or “natural” remedies, vitamins,  and other over-the-counter medications, should be reviewed to assess the potential for  **pharmacokinetic and pharmacodynamic interactions** with psychotropic drugs.  [I] |
| **Adopt** | **Allergies or sensitivities** to medications should be recorded.  [I] |
| **Adopt** | A mental status examination, including an **evaluation of insight and judgment**, should be performed to  systematically collect and record data related to the patient’s signs and symptoms of illness during the  interview.  [I] |
|  | **Establishing Goals for Treatment** |
| **Adopt** | Clinical recovery and full remission, if they occur, do not occur rapidly. Thus, ongoing **goals of treatment**  include decreasing symptom frequency and severity, improving the patient’s functioning, and helping the  patient to improve his or her quality of life.  [I] |
| **Adopt** | Treatment goals also include enhancing the patient’s ability to **cooperate with care** despite the frightening  cognitions generated by OCD, **minimizing any adverse effects** of treatment (e.g., medication side effects),  helping the patient **develop coping strategies** for stressors, and **educating** the patient and family regarding  the disorder and its treatment.  [I] |
|  | **Enhancing Treatment Adherence** |
| **Adopt** | To enhance treatment adherence, the psychiatrist should **consider factors** related to the illness, the patient,  the physician, the patient-physician relationship, the treatment, and the social or environmental milieu.  [I] |
| **Adopt** | Because the **patient’s beliefs** about the nature of the illness and its treatments will influence adherence,  providing patient and family **education** may enhance adherence.  [II] |
| **Adopt** | Many patients with OCD benefit from educational materials and access to support groups provided by  the Obsessive Compulsive Foundation (www.ocfoundation.org). When a patient has insufficient  motivation to participate effectively in treatment, **motivational interviewing** or other psychosocial  interventions designed to **enhance readiness** for change may be helpful.  [II] |
| **Adopt** | Because medications used to treat OCD have side effects, particularly at high doses, adherence may  be enhanced by **informing** the patient about any likely side effects, **responding quickly** to side effect  concerns, and **scheduling follow-up** appointments soon after starting or changing medications.  [I] |
| **Adopt** | In describing cognitive-behavioral therapy (CBT), it is helpful to advise that it involves **confronting**  feared thoughts and situations, though at a **tolerable rate.**  [I] |
| **Adopt** | **Practical issues** such as treatment cost, insurance coverage, and transportation may need to be addressed.  When a patient with OCD refuses or prematurely discontinues treatment, the clinician may wish to  recommend that family members and others negatively affected by the OCD seek therapy to help  **develop strategies** to mitigate the effect of the patient’s OCD on their lives and to **encourage the**  **patient** to obtain treatment.  [II] |
|  | **Choosing an Initial Treatment Modality** |
| **Adopt** | In choosing a treatment approach, the clinician should consider the **patient’s motivation** and **ability**  **to comply** with pharmacotherapy and psychotherapy.  [I] |
| **Adopt/**  **Refer** | **CBT and serotonin reuptake inhibitors (SRIs)** are recommended as safe and effective **first-line**  **treatments** for OCD.  [I] |
| **Adopt** | Whether to utilize CBT, an SRI, or combined treatment will depend on factors that include the **nature**  **and severity** of the patient’s **symptoms**, the nature of any **co-occurring psychiatric and medical**  **conditions** and their treatments, the **availability of CBT**, and the patient’s **past treatment** history,  **current medications**, **capacities**, and **preferences.** CBT alone, consisting of exposure and response  prevention, is recommended as initial treatment for a patient who is not too depressed, anxious, or  severely ill to cooperate with this treatment modality, or who prefers not to take medications and is  willing to do the work that CBT requires.  [II] |
| **Adopt** | An SRI alone is recommended for a patient who is **not able to cooperate with CBT**, has previously  responded well to a given drug, or prefers treatment with an SRI alone.  [II] |
| **Adopt/**  **Refer** | **Combined treatment** should be considered for patients with an **unsatisfactory response** to monotherapy.  [II] |
| **Adopt/**  **Refer** | Those with **co-occurring psychiatric conditions** for which SRIs are effective.  [I] |
| **Adopt/**  **Refer** | For those who wish to **limit the duration** of SRI treatment.  [II] |
| **Adopt/**  **Refer** | In the latter instance, uncontrolled follow-up studies suggest that **CBT may delay or mitigate relapse**  when SRI treatment is discontinued.  [II] |
| **Adopt/**  **Refer** | **Combined treatment** or treatment with an SRI alone may also be considered in patients with **severe**  **OCD**, since the medication may diminish symptom severity sufficiently to allow the patient to engage  in CBT.  [II] |
| **Adopt** | Deciding whether to start or stop a psychotropic drug during **pregnancy or breast-feeding** requires  making a **risk-benefit calculation** with the patient and her significant other; this process may be  enhanced by providing clear information, seeking consultation from an obstetrician, and providing  counseling over several sessions to help the patient come to terms with the uncertainty of the risks.  [I] |
|  | **Choosing a Specific Pharmacological Treatment** |
| **Adopt** | **Clomipramine, fluoxetine, fluvoxamine, paroxetine, and sertraline**, which are approved by the  U.S. Food and Drug Administration (FDA) for treatment of OCD, are recommended pharmacological  agents.  [I] |
| **Adopt** | Although meta-analyses of placebo-controlled trials suggest greater efficacy for clomipramine than  for fluoxetine, fluvoxamine, and sertraline, the results of head-to-head trials comparing clomipramine  and selective serotonin reuptake inhibitors (SSRIs) directly do not support this impression. Because  the **SSRIs have a less troublesome side-effect** profile than clomipramine, an **SSRI is preferred** for  a first medication trial.  [I] |
| **Adopt** | Although all SSRIs (including citalopram and escitalopram) appear to be equally effective, **individual**  **patients may respond well to one medication and not to another.** In choosing among the SSRIs, the  psychiatrist should consider the safety and acceptability of **particular side effects for the patient**,  including any applicable FDA warnings, potential drug interactions, past treatment response, and the  presence of co-occurring general medical conditions.  [I] |
|  | **Choosing a Specific Form of Psychotherapy** |
| **Adopt/**  **Refer** | Some data support the use of **CBT** that focuses on **cognitive techniques**.  [II] |
| **Adopt/**  **Refer** | CBT that relies primarily on behavioral techniques such as **exposure and response prevention** (ERP)  is recommended because it has the **best evidentiary** support.  [I] |
| **Adopt/**  **Refer** | **Family therapy** may reduce **inter-family tensions** that are exacerbating the patient’s symptoms or  ameliorate the family’s collusion with symptoms.  [III] |
|  | **Implementing a Treatment Plan** |
| **Adopt** | When treatment is initiated, the patient’s motivation and adherence may be challenged by factors such  as treatment **cost** and medication **side effects**. It is essential for the psychiatrist to employ strategies to  enhance adherence, as described above in Section I.B.1.h  [I] |
|  | **Implementing Pharmacotherapy** |
| **Adopt** | For most patients, the starting dose is that **recommended by the manufacturer**.  [I] |
| **Adopt** | Patients who are worried about medication side effects can have their medication started at **lower doses**,  since many SSRIs are available in liquid form or in pills that can be split.  [I] |
| **Adopt** | Most patients will not experience substantial improvement until **4–6 weeks** after starting medication,  and some who will ultimately respond will **experience little improvement for as many as 10–12 weeks**.  Medication doses may be **titrated up weekly** in increments recommended by the manufacturer during  the first month of treatment.  [II] |
| **Adopt** | When little or no symptom improvement is seen **within 4 weeks** of starting medication, the dose may  be **increased weekly or biweekly** to the maximum dose comfortably tolerated and indicated.  [II] |
| **Adopt** | This **maximum dose may exceed the manufacturer’s recommended** maximum dose in some cases.  [III] |
| **Adopt** | The treatment trial is then **continued at this dosage for at least 6 weeks**.  [II] |
| **Adopt** | Since available trial data suggest that higher SSRI doses produce a somewhat higher response rate  and a somewhat greater magnitude of symptom relief, such doses should be **considered when treatment**  **response is inadequate**.  [II] |
| **Adopt** | Higher doses may also be appropriate for patients who have had little response to treatment and are  **tolerating a medication** well.  [I] |
| **Adopt** | If **higher doses** are prescribed, the patient should be **closely monitored** for side effects, including the  **serotonin syndrome.**  [I] |
| **Adopt** | Experience with pharmacotherapy in the **elderly** indicates that **lower starting doses** of medication and  a more gradual approach to dose increase is often advisable.  [I] |
| **Adopt** | Medication side effects should be **inquired about** and actively managed.  [I] |
| **Adopt** | Useful strategies to manage medication side effects include **gradual initial dose titration** to minimize  gastrointestinal distress.  [I] |
| **Adopt** | Addition of a **sleep-promoting agent** to minimize insomnia.  [I] |
| **Adopt** | Use of a **low-dose anticholinergic agent** to minimize sweating.  [III] |
| **Adopt** | **Sexual side effects** may be minimized by **reducing** the dose.  [II] |
| **Adopt** | Waiting for symptoms to remit.  [II] |
| **Adopt** | Trying a once-weekly, one-day **“drug holiday”** before sexual activity  [II] |
| **Adopt** | **Switching** to another SSRI  [II] |
| **Adopt** | Adding a pharmacological agent such as **bupropion.**  [II] |
| **Adopt** | The **frequency of follow-up visits** after a new pharmacotherapy is initiated may vary from a **few days**  **to two weeks**. The indicated frequency will depend on the severity of the patient’s symptoms, the  complexities introduced by co-occurring conditions, whether suicidal ideation is present, and the  likelihood of troubling side effects.  [I] |
|  | **Implementing Cognitive-Behavioral Therapies** |
| **Adopt/**  **Refer** | Cognitive-behavioral therapies have been delivered in **individual, group, and family therapy sessions**,  with session length varying from less than **1 hour to 2 hours**. One group has explored a computer-based  approach coupled with a touch-tone telephone system accessible 24 hours a day. CBT sessions should be  scheduled **at least once weekly**.  [I] |
| **Adopt/**  **Refer** | Five ERP sessions per week may be more effective than once-weekly sessions but are not necessarily  more effective than twice-weekly sessions .  [II] |
| **Adopt/**  **Refer** | The number of treatment sessions, their length, and the duration of an adequate trial have not been established,  but expert consensus recommends 13–20 weekly sessions for most patients.  [I] |
| **Adopt/**  **Refer** | When resources for CBT are not available, the psychiatrist can suggest and supervise the use of **self-help**  **treatment guides** and recommend support groups such as those accessible through the Obsessive  Compulsive Foundation  [III] |
|  | **Changing Treatments and Pursuing Sequential Treatment Trials** |
| **Adopt/**  **Refer** | First treatments **rarely produce freedom** from all OCD symptoms. When a good response is not  achieved after **13–20 weeks of weekly outpatient CBT, 3 weeks of daily CBT, or 8–12 weeks of**  **SRI treatment** (including 4–6 weeks at the highest comfortably tolerated dose), the psychiatrist should  decide with the patient when, whether, and how to alter the treatment.  [I] |
| **Adopt** | This decision will depend on **the degree of suffering and disability the patient wishes to accept**.  However, it is important to consider that illness can bring secondary gains and that depressed mood  can diminish hopefulness; the psychiatrist may have to address issues such as these when patients are  not well motivated to pursue further treatments despite limited improvement.  [I] |
| **Adopt** | When initial treatment is unsatisfactory, the psychiatrist should first consider the **possible contribution**  **of several factors**: interference by **co-occurring conditions**, inadequate patient **adherence** to treatment,  the presence of **psychosocial stressors**, the level of family members’ **accommodation** to the  obsessive-compulsive symptoms, and an inability to **tolerate an adequate trial** of psychotherapy  or the maximum recommended drug doses.  [I] |
| **Adopt** | When no interfering factor can be identified, **augmentation strategies** may be preferred to switching  strategies in patients who have a partial response to the initial treatment.  [II] |
| **Adopt/**  **Refer** | The psychiatrist should first consider augmentation of SRIs with trials of different antipsychotic  medications or with CBT consisting of ERP, or augmentation of CBT with an SRI.  [II] |
| **Adopt** | Or has a **partial response** to monotherapy**.**  [II] |
| **Adopt/**  **Refer** | Combined SRI and CBT treatment may also **reduce the chance of relapse** when medication is  discontinued.  [II] |
| **Adopt** | Patients who do not respond to their first SRI may have their **medication switched** to a different SRI.  [I] |
| **Adopt** | A switch to **venlafaxine is less likely** to produce an adequate response.  [II] |
| **Adopt** | For patients who have not benefitted from their first SSRI trial, a **switch to mirtazapine** can also be  considered.  [III] |
| **Adopt** | The available evidence does not allow one to predict the chance of response to switching medications.  SRI nonresponders, like partial responders, have responded to augmentation with antipsychotic  medications.  [II]  The available evidence **does not allow** one to **predict the chance** of response to switching medications.  SRI nonresponders, like partial responders, have **responded to augmentation with antipsychotic**  **medications**.  [II] |
| **Adopt/**  **Refer** | Or CBT.  [II] |
| **Adopt** | After first- and second-line treatments and well-supported augmentation strategies have been exhausted,  **less well-supported treatment** strategies may be considered.  [III] |
| **Adopt** | Successful medication treatment should be continued for **1–2 years** before considering a **gradual taper**  by **decrements of 10%–25% every 1–2 months** while observing for symptom return or exacerbation.  [I] |
|  | **Discontinuing Active Treatment** |
| **Adopt/**  **Refer** | Successful ERP should be followed by **monthly booster sessions** for **3–6 months**, or more intensively  if response has been only partial.  [II] |
| **Adopt** | In medication discontinuation trials, rates of relapse or trial discontinuation for insufficient clinical  response are substantial but vary widely because of major methodological differences across studies.  Thus, **discontinuation of pharmacotherapy should be carefully considered**, and for most patients,  continued treatment of some form is recommended.  [II] |
| **Adopt/**  **Refer** | The data suggest that CBT consisting of **ERP may have more durable effects** than some SRIs after  discontinuation, but the observed differences in relapse rates could be explained by other factors. |

**ACRONYMS AND ABBREVIATIONS**

| BUN | Blood urea nitrogen | OCD | Obsessive -compulsive disorder |
| --- | --- | --- | --- |
| CBT | Cognitive behavioral therapy | SSRIs | Selective serotonin reuptake inhibitors |
| Cr | Creatinine | SRIs | Serotonin reuptake inhibitors |
| CBC | Complete blood count | SSRIs | Selective serotonin reuptake inhibitors |
| DSM IV-TR | Diagnostic & statistical manual of mental disorder fourth edition- text revision | SGPT | Serum-glutamic pyruvic transaminase |
| ERP | Exposure & response prevention | TSH | Thyroid Stimulating Hormone |
| FDA | Food & Drugs Administration | Y-BOCS | Yale-brown obsessive-compulsive scale |

**SECTION 3: SCHIZOPHRENIA**

**Treatment of Patients with Schizophrenia**

**Source Guideline**: Practice Guideline for the treatment of patients with schizophrenia
American Psychiatric Association, 3^rd^ Edition. Washington, DC, American Psychiatric Publishing. 2021.^4^

**Key to understanding level of evidence and strength of recommendation**

| Quality of evidence | |
| --- | --- |
| A | High |
| B | Moderate |
| C | Low |
| Rating of Recommendations | |
| 1 | Benefits of the intervention clearly outweigh the harms |
| 2 | Indicates greater uncertainty: although benefits of statement are still viewed as outweighing the harms, the balance of benefits and harms is more difficult to judge, or the benefits or the harms may be less clear. |

**Table of Recommendations**

|  | **Assessment and Determination of Treatment Plan** |
| --- | --- |
| **Adopt** | Initial examination includes **assessing reason** the individual is presenting for evaluation; **patient’s goals**  **and preference**s for treatment; **review of psychiatric symptoms** and **trauma history**; tobacco use and  other **substance use**; **psychiatric treatment history**; **physical health**; **psychosocial and cultural factors**;  **mental status** examination, including cognitive assessment; **assessment of risk of suicide and aggressive**  **behaviours**.  [1C] |
| **Adopt** | **Quantitative measure** to identify and determine severity of symptoms and impairments of functioning  that may be a focus of treatment.  [1C] |
| **Adopt** | Patients to have a **documented, comprehensive, and person-centered treatment plan** that includes  evidence-based nonpharmacological and pharmacological treatments.  [1C] |
|  | **Pharmacotherapy** |
| **Adopt** | Treat with an **antipsychotic medication** and monitor for effectiveness and side effects  [1A] |
| **Adopt** | **Continue treatment** with an antipsychotic medication in patients whose symptoms have improved.  [1C] |
| **Adopt** | Patients whose symptoms have improved with an antipsychotic medication continue to be treated with  the same antipsychotic medication.  [2B] |
| **Adopt** | Treat patients with **treatment-resistant** schizophrenia with **clozapine (**1B)  Treat with clozapine if the risk for **suicide attempts** or suicide remains substantial despite other treatments.  [1B] |
| **Adopt** | Treat with clozapine if the risk for **aggressive behavior** remains substantial despite other treatments.  [2C] |
| **Adopt** | Offer treatment with **a long-acting injectable antipsychotic medication** if patients prefer such treatment  or they have a history of poor or uncertain adherence.  [2B] |
| **Adopt** | Treat patients who have **acute dystonia** associated with antipsychotic therapy with an **anticholinergic**  **medication**.  [1C] |
| **Adopt** | Offer treatment options to patients who have **parkinsonism** associated with antipsychotic therapy;  **lowering dosage** of antipsychotic medication, **switching** to another antipsychotic medication, or **treating**  **with an anticholinergic medication**.  [2C] |
| **Adopt** | Treatment options for patients who have **akathisia** associated with antipsychotic therapy: **lowering dosage**  of antipsychotic medication, **switching to another** medication, adding a **benzodiazepine or adding beta**  **blockers**.  [1C] |
| **Adopt** | Treat patients who have **moderate to severe or disabling tardive dyskinesia** associated with antipsychotic  therapy with a **reversible inhibitor of vesicular monoamine transporter 2 (VMAT2)**  [1B] |
|  | **Psychosocial Interventions** |
| **Adopt/**  **Refer** | Treat patients who are experiencing a **first episode of psychosis** in a **coordinated specialty care program.** [1B] |
| **Adopt/**  **Refer** | Provide treatment with **cognitive-behavioural therapy** for psychosis (CBTp).  [1B] |
| **Adopt/**  **Refer** | Offer **psychoeducation**  [1B]  supported employment services.  [1B] |
| **Adopt/**  **Refer** | Offer **assertive community treatment** if there is a history of **poor engagement** with services leading to  frequent relapse or social disruption (e.g., homelessness; legal difficulties, including imprisonment)  [1B] |
| **Adopt/**  **Refer** | Provide **family interventions** to patients who have ongoing contact with family.  [2B] |
| **Adopt/**  **Refer** | Offer interventions aimed at developing **self-management skills and enhancing person-oriented recovery.** [2C] |
| **Adopt/**  **Refer** | Offer **cognitive remediation**. [2C] |
| **Adopt/**  **Refer** | Provide patients who have a therapeutic goal of enhanced social functioning with **social skills training.** [2C] |
| **Adopt/**  **Refer** | Treat with **supportive psychotherapy.**  [2C] |

**ACRONYMS AND ABBREVIATIONS**

| BUN | blood urea nitrogen | SGPT | Serum glutamic pyruvic transaminase |
| --- | --- | --- | --- |
| CBC | complete blood count | TSH | thyroid stimulating hormone |
| CBTp | Cognitive Behavioral therapy for psychosis | VMAT2 | vesicular monoamine transporter 2 |
| Cr | creatinine |  |  |
| MRI | magnetic resonance imaging |  |  |

**SECTION 4:**

**ACUTE STRESS DISORDER & POST-TRAUMATIC STRESS DISORDER MANAGEMENT**

**Treatment of Patients with acute stress disorder & post-traumatic stress disorder**

**Source Guideline:** Practice guideline for the Treatment of Patients with Acute Stress Disorder and Posttraumatic Stress Disorder 2004.^5^

**Key to understanding level of evidence and strength of recommendation.**

| Grade of Recommendation | |
| --- | --- |
| [I] | Recommended with substantial clinical confidence |
| [II] | Recommended with moderate clinical confidence |
| [III] | May be recommended on the basis of individual circumstances |

**Table of Recommendations**

|  | **Initial Assessment** |
| --- | --- |
| **Adopt** | The initial step in identifying individuals with ASD or PTSD involves **screening for recent or remote**  **trauma exposure**, although the clinical approach may vary depending on the recency of the traumatic event.  [I] |
| **Adopt** | If eliciting vivid and detailed recollections of the traumatic event immediately after exposure enhances  the patient’s distress, the interview may be limited to **gathering information that is essential** to provide  needed medical care.  [I] |
| **Adopt** | The first interventions in the aftermath of an acute trauma consist of **stabilizing and supportive medical**  **care** and supportive psychiatric care and assessment.  [I] |
| **Adopt** | After **large-scale catastrophes**, initial psychiatric assessment includes differential diagnosis of **physical**  **and psychological effects** of the traumatic event (eg anxiety resulting from hemodynamic compromise,  hyperventilation, somatic expressions of psychological distress, fatigue) and identification of persons or  groups who are at greatest risk for subsequent psychiatric disorders, including ASD or PTSD.  [I] |
| **Adopt** | This **identification** may be accomplished through individual evaluation, group interviews, consultation,  and use of surveillance instruments.  [I] |
| **Adopt** | **Diagnostic evaluation** may be continued after the initial period has passed and a physically and  psychologically safe environment has been established, the individual’s medical condition has been  stabilized, psychological reassurance has been provided, and, in disaster settings, necessary triage has  been accomplished. It is important for this diagnostic assessment to include a complete psychiatric  evaluation that specifically assesses for the symptoms of ASD and PTSD, including dissociative,  reexperiencing, avoidance/numbing, and hyperarousal symptom clusters and their temporal sequence  relative to the trauma (i.e., before versus after 1 month from the traumatic event).  [I] |
| **Adopt** | Other important components of the assessment process include functional assessment, determining the  availability of basic care resources (e.g., safe housing, social support network, companion care, food,  clothing), and identifying previous traumatic experiences and comorbid physical or psychiatric disorders,  including depression and substance use disorders.  [I] |
|  | **Psychiatry Management** |
| **Adopt** | Psychiatric management for all patients with ASD or PTSD includes instituting interventions and  activities to ensure physical and psychological safety, required medical care, and availability of needed  resources for self-care and recovery.  [I] |
| **Adopt** | The patient’s level of functioning and safety, including his or her risk for suicide and potential to harm  others, are always important to evaluate during initial assessment and may determine the treatment setting.  [I] |
| **Adopt** | The **goals of psychiatric management** for patients with ASD and PTSD also include establishing a  **therapeutic alliance** with the patient; providing ongoing assessment of safety and psychiatric status,  including possible comorbid disorders and response to treatment; and increasing the patient’s understanding  of and active adaptive coping with psychosocial effects of exposure to the traumatic event, such as injury,  job loss, or loss of loved ones.  [I] |
| **Adopt** | **Additional goals of psychiatric management** include providing education regarding ASD and PTSD,  enhancing treatment adherence, evaluating and managing physical health and functional impairments,  and coordinating care to include collaborating with other clinicians.  [I] |
|  | **General Principles of Treatment selection** |
| **Adopt** | The **goals of treatment** for individuals with a diagnosis of ASD or PTSD include reducing the severity  of ASD or PTSD symptoms, preventing or treating trauma-related comorbid conditions that may be  present or emerge, improving adaptive functioning and restoring a psychological sense of safety and  trust, limiting the generalization of the danger experienced as a result of the traumatic situation(s),  and protecting against relapse.  [I] |
| **Adopt** | Patients assessed within hours or days after an acute trauma may present with **overwhelming**  **physiological and emotional symptoms** (e.g., insomnia, agitation, emotional pain, dissociation).  Limited clinical trial evidence is available in this area, as randomized designs are difficult  to implement; however, clinical experience suggests that these acutely traumatized individuals may  benefit from supportive psychotherapeutic and psychoeducational interventions.  [II] |
| **Adopt** | **Pharmacotherapy** may be the first-line intervention for acutely traumatized patients whose degree of  distress precludes new verbal learning or non-pharmacological treatment strategies.  [II] |
| **Adopt** | Research has not consistently identified **patient- or trauma-specific factors** that predict the development  of ASD or interventions that will alter the evolution of ASD into PTSD. However, early after a trauma,  once the patient’s safety and medical stabilization have been addressed, supportive psychotherapy,  psychoeducation, and assistance in obtaining resources such as food and shelter and locating family  and friends are useful.  [II] |
| **Adopt** | Effective treatments for the symptoms of ASD or PTSD encompass **psychopharmacology,**  **psychotherapy, and psychoeducation** and other supportive measures.  [I] |
| **Adopt** | Although studies using a combination of these approaches for ASD and PTSD are not presently available,  combination treatment is widely used and may offer advantages for some patients.  [II] |
| **Adopt** | For patients with ASD or PTSD, choice of treatment includes **consideration of age and gender**, presence  of **comorbid medical and psychiatric illnesses**, and propensity for aggression or self-injurious behaviour.  [I] |
| **Adopt** | Other factors that may influence treatment choice include the **recency** of the precipitating traumatic event;  the **severity and pattern** of symptoms; the presence of particularly distressing **target symptoms** or  symptom clusters; the development of **interpersonal or family issues** or occupational or work-related  problems; **pre-existing developmental** or **psychological vulnerabilities**, including prior trauma exposure;  and the patient’s preferences.  [I] |
| **Adopt** | When the patient’s symptoms do not respond to a plan of treatment, selection of subsequent interventions  will depend on clinical judgment, as there is limited data to guide the clinician. It is important to  **systematically review factors** that may contribute to treatment nonresponse, including the specifics  of the initial treatment plan and its goals and rationale, the patient’s perceptions of the effects of treatment,  the patient’s understanding of and adherence to the treatment plan, and the patient’s reasons for nonadherence  if nonadherence is a factor.  [I] |
| **Adopt** | Other factors that may need to be addressed in patients who are not responding to treatment include  **problems in the therapeutic alliance**; the presence of **psychosocial or environmental difficulties**; the  effect of **earlier life experiences** such as childhood abuse or previous trauma exposures; and comorbid  psychiatric disorders, including substance-related disorders and personality disorders.  [I] |
|  | **Specific treatment strategies**  **(a) Psychopharmacology** |
| **Adopt** | Although it has been hypothesized that pharmacological treatment soon after trauma exposure may prevent  the development of ASD and PTSD, existing evidence is limited and preliminary. Thus, no specific  pharmacological interventions can be recommended as efficacious in preventing the development of ASD  or PTSD in at-risk individuals |
| **Adopt** | **SSRIs** are recommended as **first-line medication** treatment for PTSD.  [I] |
| **Adopt** | Other antidepressants, including **tricyclic antidepressants and monoamine oxidase inhibitors** (MAOIs),  may also be beneficial in the treatment of PTSD.  [II] |
| **Adopt** | **Benzodiazepines** may be useful in **reducing anxiety and improving sleep**  [III] |
| **Adopt** | In addition to being indicated in patients with comorbid psychotic disorders, **second generation**  **antipsychotic medications** (e.g., olanzapine, quetiapine, risperidone) **may be helpful** in individual patients  with PTSD.  [III] |
| **Adopt** | **Anticonvulsant medications** (e.g., divalproex, carbamazepine, topiramate, lamotrigine), **α2-adrenergic**  **agonists, and β-adrenergic blockers** may also be helpful in treating specific symptom clusters in individual  patients.  [III] |
|  | **(b) Psychotherapeutic interventions** |
| **Adopt/**  **Refer** | Some evidence is available about the effectiveness of psychotherapeutic intervention immediately after  trauma in preventing development of ASD or PTSD. Studies of **cognitive behavior therapy** in motor  vehicle and industrial accident survivors as well as in victims of rape and interpersonal violence suggest  that cognitive behaviour therapies **may speed recovery and prevent PTSD** when therapy is given over  a few sessions beginning 2–3 weeks after trauma exposure.  [II] |
| **Adopt/**  **Refer** | **Early supportive interventions**, psychoeducation, and case management appear to be helpful in acutely  traumatized individuals, because these approaches promote engagement in ongoing care and may facilitate  entry into evidence-based psychotherapeutic and psychopharmacological treatments.  [II] |
| **Adopt/**  **Refer** | Encouraging acutely traumatized persons to first rely on their inherent strengths, their existing support  networks, and their own judgment may also reduce the need for further intervention.  [II] |
| **Adopt/**  **Refer** | Patients with **ASD** may be helped by **cognitive behaviour therapy** and other exposure-based therapies.  [II] |
| **Adopt/**  **Refer** | In addition, **cognitive behaviour therapy** is an effective treatment for core symptoms of acute and chronic  PTSD.  [I] |
| **Adopt/**  **Refer** | **EMDR** is also effective.  [II] |
| **Adopt/**  **Refer** | **Stress inoculation, imagery rehearsal, and prolonged exposure** techniques may also be indicated for  treatment of PTSD and PTSD-associated symptoms such as anxiety and avoidance. [II] |
| **Adopt/**  **Refer** | **Psychodynamic psychotherapy** may be useful in addressing developmental, interpersonal, or intrapersonal  issues that relate to the nature, severity, symptoms, or treatment of ASD and PTSD and that may be of  particular importance to social, occupational, and interpersonal functioning.  [II] |
| **Adopt/**  **Refer** | **Case management, psychoeducation, and other supportive interventions** may be useful in facilitating  entry into ongoing treatment, appear not to exacerbate PTSD symptoms, and in some pilot investigations  have been associated with PTSD symptom reduction.  [II]  Present centered and trauma-focused group therapies may also reduce PTSD symptom severity. [III] |

**ACRONYMS AND ABBREVIATIONS**

| ASD | Acute Stress Disorder | MAOIs | Monoamine Oxidase Inhibitors |
| --- | --- | --- | --- |
| BUN | Blood Urea Nitrogen | PTSD | Post-Traumatic Stress Disorder |
| Cr | Creatinine | SSRIs | Selective Serotonin Reuptake Inhibitors |
| CBC | Complete blood count | SGPT | Serum Glutamic Pyruvic Transaminase |
| EMDR | Eye Movement Desensitization and Re-processing | TSH | Thyroid Stimulating Hormone |

**SECTION 5: MAJOR DEPRESSIVE DISORDER**

**Major Depressive Disorder Management
Source Guideline:** Practice Guideline for the treatment of patients with major depressive
American Psychiatric Association. 2002.^6^

**Key to understanding level of evidence and strength of recommendation.**

| Grades of Recommendations | |
| --- | --- |
| [I] | Recommended with substantial clinical confidence |
| [II] | Recommended with moderate clinical confidence |
| [III] | May be recommended based on individual circumstances |

**Table of Recommendations**

|  | **Psychiatric management** |
| --- | --- |
| **Adopt** | Psychiatric management consists of a broad array of interventions and activities that psychiatrists should  initiate and continue to provide to patients with major depressive disorder through all phases of treatment.  [I] |
| **Adopt** | Establish and maintain a **therapeutic alliance** - it is important to collaborate with the patient in decision  making and attend to the **patient’s preferences and concerns** about treatment.  [I] |
| **Adopt** | Management of the therapeutic alliance should include **awareness of transference and counter-**  **transference issues**, even if these are not directly addressed in treatment.  [II] |
| **Adopt** | Severe or persistent problems of poor alliance or nonadherence to treatment may be caused by the  **depressive symptoms** themselves or may represent psychological conflicts or **psychopathology** for  which psychotherapy should be considered.  [II] |
|  | **Complete the psychiatric assessment** |
| **Adopt** | Patients should receive a thorough **diagnostic assessment** in order to establish the diagnosis of major  depressive disorder, identify other psychiatric or general medical conditions that may require attention,  and develop a comprehensive  plan for treatment.  [I] |
| **Adopt** | This evaluation generally includes a **history** of the present illness and current symptoms; a psychiatric  history, including identification of past symptoms of mania, hypomania, or mixed episodes and responses  to previous treatments; a general medical history; a personal history including information about  psychological development and responses to life transitions and major life events; a social, occupational,  and family history (including mood disorders and suicide); review of the patient’s prescribed and  over-the-counter medications; a review of systems; a mental status examination; a physical examination;  and appropriate diagnostic tests as indicated to rule out possible general medical causes of depressive  symptoms.  [I] |
| **Adopt** | **Assessment of substance use** should evaluate past and current use of illicit drugs and other substances  that may trigger or exacerbate depressive symptoms.  [I] |
|  | **Evaluate the safety of the patient** |
| **Adopt** | A careful and ongoing **evaluation of suicide risk** is necessary for all patients with major depressive disorder.  [I] |
| **Adopt** | Such an assessment includes **specific inquiry** about suicidal thoughts, intent, plans, means, and behaviors;  identification of specific psychiatric **symptoms** (e.g., psychosis, severe anxiety, substance use) or general  **medical conditions** that may increase the likelihood of acting on suicidal ideas, assessment of past and,  particularly, recent suicidal **behavior**; delineation of current **stressors** and potential protective factors  (e.g., positive reasons for living, strong social support); and identification of any **family history** of  suicide or mental illness.  [I] |
| **Adopt** | Assess the patient’s level of **self-care, hydration, and nutrition**, each of which can be compromised  by severe depressive symptoms.  [I] |
| **Adopt** | **Impulsivity and potential for risk to others** should also be evaluated, including any history of violence  or violent or homicidal ideas, plans, or intentions.  [I] |
| **Adopt** | An evaluation of the impact of the depression on the patient’s **ability to care for dependents** is an  important component of the safety evaluation.  [I] |
| **Adopt** | The patient’s **risk of harm to him- or herself** and to others should also be monitored as treatment  proceeds.  [I] |
|  | **Establish the appropriate setting for treatment** |
| **Adopt** | The physician should determine the **least restrictive setting** for treatment that will be most likely not  only to address the patient’s safety, but also to promote improvement in the patient’s condition.  [I] |
| **Adopt** | The determination of an appropriate setting for treatment should include consideration of the patient’s  symptom severity, co-occurring psychiatric or general medical conditions, available support system,  and level of functioning.  [I] |
| **Adopt** | The determination of a treatment setting should also include consideration of the patient’s ability to  adequately care for him- or herself, to provide reliable feedback to the psychiatrist, and to cooperate  with treatment of the major depressive disorder.  [I] |
| **Refer** | Measures such as **hospitalization** should be considered for patients who **pose a serious threat of harm**  **to themselves or others**.  [I] |
| **Adopt** | The **optimal treatment** setting and the patient’s likelihood of benefit from a different level of care should  be **reevaluated on an ongoing basis** throughout the course of treatment.  [I] |
|  | **Evaluate functional impairment and quality of life** |
| **Adopt** | Major depressive disorder can alter functioning in **numerous spheres of life** including work, school,  family, social relationships, leisure activities, or maintenance of health and hygiene. The psychiatrist  should evaluate the patient’s activity in each of these domains and determine the presence, type, severity,  and chronicity of any dysfunction.  [I] |
| **Adopt** | Interventions should be aimed at **maximizing the patient’s level of functioning** as well as helping the  patient to set specific goals appropriate to his or her functional impairments and symptom severity.  [I] |
|  | **Coordinate the patient’s care with other clinicians** |
| **Adopt** | If more than one clinician is involved in providing the care, all treating clinicians should have sufficient  ongoing contact with the patient and with each other to ensure that **care is coordinated**, relevant  information is available to guide treatment decisions, and treatments are synchronized.  [I] |
| **Adopt** | In ruling out general medical causes of depressive symptoms, it is important to ensure that **a general**  **medical evaluation** has been done  [I]  either by the psychiatrist or by another health care professional. |
| **Adopt** | Extensive or specialized testing for general medical causes of depressive symptoms may be conducted  based on individual characteristics of the patient.  [III] |
|  | **Monitor the patient’s psychiatric status** |
| **Adopt** | The patient’s **response to treatment** should be carefully monitored.  [I] |
| **Adopt** | **Continued monitoring** of co-occurring psychiatric and/or medical conditions is also essential to  developing and refining a treatment plan for an individual patient.  [I] |
|  | **Integrate measurements into psychiatric management** |
| **Adopt** | Tailoring the treatment plan to **match the needs** of the particular patient requires a careful and systematic  assessment of the type, frequency, and magnitude of psychiatric symptoms as well as ongoing determination  of the therapeutic benefits and side effects of treatment.  [I] |
| **Adopt** | Such assessments can be facilitated by integrating clinician- and/or patient-administered **rating scale**  **measurements** into initial and ongoing evaluation.  [II] |
|  | **Enhance treatment adherence** |
| **Adopt** | The physician should assess and acknowledge potential **barriers to treatment adherence** (e.g., lack of  motivation or excessive pessimism due to depression; side effects of treatment; problems in the therapeutic  relationship; logistical, economic, or cultural barriers to treatment) and collaborate with the patient (and if  possible, the family) to minimize the impact of these potential barriers.  [I] |
| **Adopt** | In addition, the physician should encourage patients to articulate any fears or concerns about treatment  or its side effects.  [I] |
| **Adopt** | Patients should be given a **realistic notion** of what can be expected during the different phases of  treatment, including the likely time course of symptom response and the importance of adherence for  successful treatment and prophylaxis.  [I] |
|  | **Provide education to the patient and the family** |
| **Adopt** | Education about the symptoms and treatment of major depressive disorder should be provided in  **language** that is readily **understandable** to the patient.  [I] |
| **Adopt** | With the patient’s permission, **family members and others** involved in the patient’s day-to-day life  may also benefit from education about the illness, its effects on functioning (including family and other  interpersonal relationships), and its treatment.  [I] |
| **Adopt** | **Common misperceptions** about antidepressants (e.g., they are addictive) should be clarified.  [I] |
| **Adopt** | In addition, education about major depressive disorder should address the need for a full acute course  of treatment, the risk of relapse, the early recognition of recurrent symptoms, and the need to seek  treatment as early as possible to reduce the risk of complications or a full-blown episode of major  depression.  [I] |
| **Adopt** | Patients should also be told about the need to **taper antidepressants**, rather than discontinuing them  precipitously, to minimize the risk of withdrawal symptoms or symptom recurrence.  [I] |
| **Adopt** | Patient education also includes general **promotion of healthy behaviors** such as exercise, good sleep  hygiene, good nutrition, and decreased use of tobacco, alcohol, and other potentially deleterious substances.  [I] |
| **Adopt** | **Educational tools** such as books, pamphlets, and trusted web sites can **augment** the face-to-face  education provided by the clinician.  [I] |
|  | **Acute Phase** |
|  | **Choice of an initial treatment modality** |
| **Adopt** | Treatment in the acute phase should be aimed at **inducing remission** of the major depressive episode  and achieving a full return to the patient’s baseline level of functioning.  [I] |
| **Adopt/**  **Refer** | Acute phase treatment may include pharmacotherapy, depression-focused psychotherapy, the combination  of medications and psychotherapy. [I] |
| **Adopt** | **Bright light therapy** might be used to treat seasonal affective disorder as well as nonseasonal depression [III] |
|  | **Pharmacotherapy** |
| **Adopt** | An **antidepressant medication** is recommended as an initial treatment choice for patients with  **mild to moderate** major depressive disorder. [I] |
| **Adopt** | Should be provided for those with **severe major depressive disorder** unless ECT is planned.  [I] |
| **Adopt** | Because the effectiveness of antidepressant medications is generally comparable between classes  and within classes of medications, the initial selection of an antidepressant medication will largely  be **based on the anticipated side effects**, the safety or tolerability of these side effects for the  individual patient, pharmacological properties of the medication (e.g., half-life, actions on cytochrome  P450 enzymes, other drug interactions), and additional factors such as medication response in prior  episodes, cost, and patient preference.  [I] |
| **Adopt** | For most patients, a selective serotonin reuptake inhibitor (SSRI), serotonin norepinephrine reuptake  inhibitor (SNRI), mirtazapine, or bupropion is optimal.  [I] |
| **Adopt** | In patients who prefer complementary and alternative therapies, S-adenosyl methionine (SAMe)  [III]  or St. John’s wort  [III]  might be considered |
| **Adopt** | **Evidence for their efficacy is modest** at best, and careful attention to drug-drug interactions is needed  with St. John’s wort.  [I] |
| **Adopt** | Once an antidepressant medication has been initiated, the **rate at which it is titrated** to a full therapeutic  dose should depend upon the patient’s age, the treatment setting, and the presence of co-occurring  illnesses, concomitant pharmacotherapy, or medication side effects.  [I] |
| **Adopt** | During the **acute phase** of treatment, patients should be carefully and systematically **monitored on a**  **regular basis** to assess their response to pharmacotherapy, identify the emergence of side effects  (e.g., gastrointestinal symptoms, sedation, insomnia, activation, changes in weight, and cardiovascular,  neurological, anticholinergic, or sexual side effects), and assess patient safety.  [I] |
| **Adopt** | The **frequency of patient monitoring** should be determined based upon the patient’s symptom severity  (including suicidal ideas), co-occurring disorders (including general medical conditions), cooperation  with treatment, availability of social supports, and the frequency and severity of side effects with the  chosen treatment.  [II] |
| **Adopt** | If antidepressant side effects do occur, an initial strategy is to **lower the dose** of the antidepressant  or to **change** to an antidepressant that is not associated with that side effect.  [I] |
|  | **Other somatic therapies** |
| **Refer** | **ECT** is recommended as a treatment of choice for patients with **severe major depressive disorder**  that is **not responsive** to psychotherapeutic and/or pharmacological interventions, particularly in those  who have significant functional impairment or have not responded to numerous medication trials.  [I] |
| **Refer** | ECT is also recommended for individuals with major depressive disorder who have **associated**  **psychotic or catatonic features**.  [I] |
| **Refer** | For those with an **urgent need for response** (e.g., patients who are suicidal or nutritionally compromised  due to refusal of food or fluids).  [I] |
| **Refer** | For those who **prefer ECT** or have had a previous positive response to ECT.  [II] |
| **Adopt/**  **Refer** | Factors that may suggest the use of **psychotherapeutic interventions** include the presence of significant  psychosocial stressors, intrapsychic conflict, interpersonal difficulties, a co-occurring axis II disorder,  treatment availability, or— most important—**patient preference**.  [II] |
| **Adopt/**  **Refer** | In women who are **pregnant**, wish to become pregnant, or are breastfeeding, a **depression-focused**  **psychotherapy** alone is recommended.  [II] |
| **Adopt/**  **Refer** | Depending on the severity of symptoms, psychotherapy should be considered as an **initial option**  [I] |
| **Adopt/**  **Refer** | As with patients who are receiving pharmacotherapy, patients receiving psychotherapy should be carefully  and systematically **monitored on a regular basis** to assess their response to treatment and assess patient  safety.  [I] |
| **Adopt/**  **Refer** | **Marital and family problems** are common in the course of major depressive disorder, and such  problems should be identified and addressed, using marital or family therapy when indicated.  [II] |
|  | **Psychotherapy plus antidepressant medication** |
| **Adopt/**  **Refer** | The **combination** of psychotherapy and antidepressant medication may be used as an initial treatment  for patients with **moderate to severe** major depressive disorder.  [I] |
| **Adopt/**  **Refer** | In addition, combining psychotherapy and medication may be a useful initial treatment even in milder  cases for patients with **psychosocial or interpersonal problems, intrapsychic conflict, or co-occurring**  **Axis II disorder**.  [II] |
| **Adopt/**  **Refer** | In general, when choosing an antidepressant or psychotherapeutic approach for combination treatment,  the same issues should be considered as when selecting a medication or psychotherapy for use alone.  [I] |
|  | **Assessing the adequacy of treatment response** |
| **Adopt** | In assessing the adequacy of a therapeutic intervention, it is important to establish that treatment has  been administered for a **sufficient duration** and at a **sufficient frequency** or, in the case of medication,  dose.  [I] |
| **Adopt** | Onset of benefit from psychotherapy tends to be a bit more gradual than that from medication, but no  treatment should continue unmodified if there has been no symptomatic improvement after **1 month**.  [I] |
| **Adopt** | Generally, **4–8 weeks of treatment** are needed before concluding that a patient is partially responsive  or unresponsive to a specific intervention.  [II] |
|  | **Strategies to address nonresponse** |
| **Adopt** | For individuals who have not responded fully to treatment, the acute phase of treatment should  not be concluded prematurely  [I]  as an incomplete response to treatment is often associated with **poor functional outcomes.** |
| **Adopt** | If at least a moderate improvement in symptoms is not observed within **4–8 weeks** of treatment  initiation, the diagnosis should be **reappraised**, side effects assessed, complicating co-occurring  conditions and psychosocial factors reviewed, and the treatment plan adjusted.  [I] |
| **Adopt** | It is also important to assess the **quality of the therapeutic alliance** and **treatment adherence.**  [I] |
| **Adopt** | If medications are prescribed, the psychiatrist should determine whether **pharmacokinetic**.  [I] |
| **Adopt** | Or **pharmacodynamic**  [III]  factors suggest a need to adjust medication doses. |
| **Adopt** | After an additional 4–8 weeks of treatment, if the patient continues to show minimal or no improvement  in symptoms, the psychiatrist should conduct another thorough review of possible contributory factors  and make additional changes in the treatment plan.  [I] |
| **Adopt** | **Consultation** should also be considered.  [II] |
| **Adopt** | A number of strategies are available when a change in the treatment plan seems necessary. For patients  treated with an antidepressant, **optimizing the medication dose** is a reasonable first step if the side  effect burden is tolerable and the upper limit of a medication dose has not been reached.  [II] |
| **Adopt** | Patients may be **changed** to an antidepressant from the **same pharmacological class** (e.g., from one  SSRI to another SSRI) or to one from a different class (e.g., from an SSRI to a tricyclic antidepressant  [TCA]).  [II] |
| **Adopt** | For patients who have not responded to trials of SSRIs, a trial of an **SNRI** may be helpful.  [II] |
| **Adopt** | Augmentation of antidepressant medications can utilize another **non-MAOI** antidepressant.  [II] |
| **Adopt** | Generally, from a **different pharmacological class**, or a non-antidepressant medication such as lithium.  [II] |
| **Adopt** | Thyroid hormone  [II] |
| **Adopt** | Or a second-generation antipsychotic.  [II] |
| **Adopt** | Additional strategies with less evidence for efficacy include **augmentation** using an anticonvulsant [III],  omega-3 fatty acids.  [III] |
| **Adopt** | Folate  [III] |
| **Adopt** | Psychostimulant medication.  [III] |
| **Adopt** | Including modafinil  [III] |
| **Adopt** | If anxiety or insomnia are prominent features, consideration can be given to anxiolytic and sedative-  hypnotic medications [III], including buspirone, benzodiazepines, and selective γ-aminobutyric acid  (GABA) agonist hypnotics (e.g., zolpidem, eszopiclone). |
| **Refer** | For patients whose symptoms have **not responded** adequately to medication, **ECT** remains the most  effective form of therapy and should be considered.  [I] |
|  | **Continuation phase** |
| **Adopt** | During the continuation phase of treatment, the patient should be carefully **monitored** for signs of  possible **relapse.**  [I] |
| **Adopt** | Systematic assessment of **symptoms, side effects, adherence, and functional status** is essential.  [I] |
| **Adopt** | May be facilitated through the use of **clinician**- and/or **patient-administered rating scales.**  [II] |
| **Adopt** | To reduce the **risk of relapse**, patients who have been treated successfully with antidepressant  medications in the acute phase should **continue treatment** with these agents for **4–9 months**.  [I] |
| **Adopt** | In general, the dose used in the acute phase should be used in the continuation phase.  [II] |
| **Adopt/**  **Refer** | To prevent a relapse of depression in the continuation phase, **depression-focused psychotherapy**  is recommended  [I]  with the best evidence available for **CBT**. |
| **Adopt** | Patients who respond to an acute course of ECT should receive continuation pharmacotherapy.  [I] |
| **Refer** | The best evidence available for the combination of lithium and nortriptyline. Alternatively, patients  who have responded to an acute course of ECT may be given **continuation ECT**, particularly **if**  **medication or psychotherapy has been ineffective** in maintaining remission.  [II] |
|  | **Maintenance phase** |
| **Adopt** | In order to reduce the risk of a recurrent depressive episode, patients who have had **three or more**  prior major depressive episodes or who have **chronic major depressive disorder** should proceed to  the maintenance phase of treatment after completing the continuation phase  [I] |
| **Adopt** | Maintenance therapy should also be considered for **patients with additional risk factors** for recurrence,  such as the presence of residual symptoms, ongoing psychosocial stressors, early age at onset, and family  history of mood disorders.  [II] |
| **Adopt** | **Additional considerations** that may play a role in the decision to use maintenance therapy include patient  preference, the type of treatment received, the presence of side effects during continuation therapy, the  probability of recurrence, the frequency and severity of prior depressive episodes (including factors such  as psychosis or suicide risk), the persistence of depressive symptoms after recovery, and the presence of  co-occurring disorders.  [II] |
| **Adopt** | Such factors also contribute to decisions about the duration of the maintenance phase.  [II]  For many patients, particularly for those with **chronic and recurrent** major depressive disorder or  **co-occurring medical and/or psychiatric disorders**, some form of maintenance treatment will be  required indefinitely.  [I] |
| **Adopt** | During the maintenance phase, an antidepressant medication that produced symptom remission during  the acute phase and maintained remission during the continuation phase should be continued at a **full**  **therapeutic dose**.  [II] |
| **Adopt/**  **Refer** | If a **depression-focused psychotherapy** has been used during the acute and continuation phases of  treatment, maintenance treatment should be considered, with a **reduced** frequency of sessions.  [II] |
| **Refer** | For patients whose depressive episodes have not previously responded to acute or continuation treatment  with medications or a depression focused psychotherapy but who have shown a response to ECT,  **maintenance ECT** may be considered.  [III] |
| **Adopt** | Due to the risk of recurrence, patients should be monitored systematically and at regular intervals  during the maintenance phase.  [I]  Use of **standardized measurement aids** in the early detection of recurrent symptoms.  [II] |
|  | **Discontinuation of treatment** |
| **Adopt** | When pharmacotherapy is being discontinued, it is best to **taper** the medication over the course of  at least **several weeks.**  [I] |
| **Adopt** | To minimize the likelihood of discontinuation symptoms, patients should be advised **not to stop**  **medications abruptly** and to take medications with them when they travel or are away from home.  [I] |
| **Adopt** | A **slow taper** or temporary change to a **longer half-life antidepressant** may reduce the risk of  **discontinuation syndrome**  [II]  when discontinuing antidepressants or reducing antidepressant doses |
| **Adopt** | Before the discontinuation of active treatment, patients should be informed of the potential for a  **depressive relapse** and a **plan** should be established for seeking treatment in the event of recurrent  symptoms.  [I] |
| **Adopt** | After discontinuation of medications, patients should continue to be **monitored** over the next **several**  **months** and should receive another course of adequate acute phase treatment if symptoms recur.  [I] |
| **Adopt/**  **Refer** | For patients receiving **psychotherapy**, it is important to **raise the issue of treatment discontinuation**  well in advance of the final session  [I]  although the exact process by which this occurs will vary with the type of therapy. |
|  | **Clinical factors influencing treatment** |
|  | **Psychiatric factors** |
| **Refer** | For **suicidal patients**, psychiatrists should consider an increased intensity of treatment, including  **hospitalization** when warranted  [I] |
| **Adopt/**  **Refer** | **Combined treatment** with pharmacotherapy and psychotherapy.  [II] |
| **Adopt** | **Factors to consider** in determining the nature and intensity of treatment include (but are not limited  to) the nature of the doctor-patient alliance, the availability and adequacy of social supports, access to  and lethality of suicide means, the presence of a co-occurring substance use disorder, and past and  family history of suicidal behavior.  [I] |
| **Adopt/**  **Refer** | For patients who exhibit **psychotic symptoms** during an episode of major depressive disorder, treatment  should include a combination of **antipsychotic and antidepressant** medications or ECT.  [I] |
| **Adopt/**  **Refer** | When patients exhibit **cognitive dysfunction** during a major depressive episode, they may have an  increased likelihood of future dementia, making it important to assess cognition in a systematic fashion  over the course of treatment.  [I] |
| **Adopt** | **Catatonic features** that occur as part of a major depressive episode should be treated with a  **benzodiazepine**.  [I] |
| **Adopt** | Typically in conjunction with an **antidepressant**.  [II] |
| **Refer** | If catatonic symptoms persist, **ECT** is recommended.  [I] |
| **Adopt** | To reduce the likelihood of general medical complications, patients with catatonia may also require  **supportive medical interventions**, such as hydration, nutritional support, prophylaxis against deep  vein thrombosis, turning to reduce risks of decubitus ulcers, and passive range of motion to reduce  risk of contractures.  [I] |
| **Adopt** | If antipsychotic medication is needed, it is important to **monitor for signs of neuroleptic malignant**  **syndrome**, to which patients with catatonia may have a heightened sensitivity.  [II] |
| **Adopt** | When patients with a major depressive disorder also have a **co-occurring psychiatric illness**, the  clinician should address each disorder as part of the treatment plan.  [I] |
| **Adopt** | **Benzodiazepines** may be used adjunctively in individuals with major depressive disorder and  co-occurring **anxiety**.  [II] |
| **Adopt** | Although these agents do not treat depressive symptoms, and careful selection and monitoring is needed  in individuals with co-occurring **substance use disorders**.  [I] |
| **Adopt** | In patients who **smoke**, **bupropion**.  [I] |
| **Adopt** | Or **nortriptyline**  [II]  may be options to simultaneously treat depression and assist with **smoking cessation**. |
| **Adopt** | For patients who have a **personality disorder** as well as major depressive disorder, psychiatrists should  institute treatment for the major depressive disorder.  [I] |
| **Adopt**/  **Refer** | Consider **psychotherapeutic and adjunctive pharmacotherapeutic** treatment for personality disorder  symptoms.  [II] |
|  | **Demographic and psychosocial factors** |
| **Adopt** | Several aspects of assessment and treatment differ between women and men. Because the symptoms of  some **women** may fluctuate with **gonadal hormone levels**, the evaluation should include a detailed  assessment of **mood changes** across the **reproductive life history** (e.g., menstruation, pregnancy, birth  control including oral contraception use, abortions, menopause).  [I] |
| **Adopt** | When prescribing medications to women who are taking **oral contraceptives**, the potential effects of  **drug-drug interactions** must be considered.  [I] |
| **Adopt** | For women in the perimenopausal period, **SSRI and SNRI antidepressants** are useful in ameliorating  depression as well as in reducing **somatic symptoms** such as **hot flashes**.  [II] |
| **Adopt** | Both **men and women** who are taking antidepressants should be asked whether **sexual side effects**  are occurring with these medications.  [I] |
| **Adopt** | Men for whom **trazodone** is prescribed should be warned of the risk of **priapism.**  [I] |
| **Adopt** | The treatment of major depressive disorder in women who are **pregnant** or planning to become pregnant  requires a careful **consideration of the benefits and risks** of available treatment options for the patient  and the fetus.  [I] |
| **Adopt** | For women who are currently receiving treatment for depression, **pregnancy should be planned**,  whenever possible, in consultation with the treating psychiatrist, who may wish to consult with a  specialist in perinatal psychiatry.  [I] |
| **Adopt/**  **Refer** | In women who are pregnant, planning to become pregnant, or breast-feeding, **depression-focused**  **psychotherapy** alone is recommended.  [II] |
| **Adopt/**  **Refer** | Should always be **considered as an initial option**, particularly for mild to moderate depression, for  patients who prefer psychotherapy, or for those with a prior positive response to psychotherapy.  [I] |
| **Adopt** | **Antidepressant medication** should be considered for **pregnant women** who have **moderate to severe**  major depressive disorder as well as for those who are in remission from major depressive disorder, are  receiving maintenance medication, and are deemed to be at **high risk for a recurrence** if the medication  is discontinued.  [II] |
| **Adopt** | When antidepressants are prescribed to a pregnant woman, changes in pharmacokinetics during pregnancy  may require **adjustments in medication doses**.  [I] |
| **Refer** | **Electroconvulsive therapy** may be considered for the treatment of depression during pregnancy in  patients who have **psychotic or catatonic features**, whose symptoms are severe or have not responded  to medications, or who prefer treatment with ECT.  [II] |
| **Adopt** | When a woman decides to **nurse**, the **potential benefits** of antidepressant medications for the mother  should be **balanced against the potential risks** to the newborn from receiving antidepressants through  the mother’s milk.  [I] |
| **Adopt** | For women who are depressed during the postpartum period, it is important to **evaluate** for the presence  of **suicidal ideas, homicidal ideas, and psychotic symptoms**.  [I] |
| **Adopt** | The evaluation should also assess **parenting skills** for the newborn and for other children in the patient’s  care.  [I] |
| **Adopt** | In individuals with **late-life depression**, identification of **co-occurring general medical conditions** is  essential, as these disorders may mimic depression or affect choice or dosing of medications.  [I] |
| **Adopt** | **Older individuals** may also be particularly **sensitive** to medication **side effects** (e.g., hypotension,  anticholinergic effects) and require adjustment of medication doses for **hepatic or renal dysfunction**.  [I] |
| **Adopt** | In other respects, treatment for depression should parallel that used in younger age groups.  [I] |
| **Adopt** | The assessment and treatment of major depressive disorder should consider the impact of **language**  **barriers**, as well as **cultural variables** that may influence symptom presentation, treatment preferences,  and the degree to which psychiatric illness is stigmatized.  [I] |
| **Adopt** | When antidepressants are prescribed, the psychiatrist should recognize that **ethnic groups may differ**  in their **metabolism** and **response to medications**.  [II] |
| **Adopt** | Issues relating to the **family situation and family history**, including mood disorders and suicide, can  also affect treatment planning and are an important element of the initial evaluation.  [I] |
| **Adopt** | A **family history of bipolar disorder** or acute psychosis suggests a need for increased attention to  possible signs of bipolar illness in the patient (e.g., with antidepressant treatment).  [I] |
| **Adopt** | A **family history of recurrent major depressive disorder** increases the likelihood of recurrent episodes  in the patient and supports a need for maintenance treatment.  [II] |
| **Adopt** | **Family history of a response to a particular antidepressant** may sometimes help in choosing a  specific antidepressant for the patient  [III] |
| **Adopt**/  **Refer** | **Problems within the family** may become an ongoing stressor that **hampers** the patient’s response  to treatment, and because depression in a family is a major stress in itself, such factors should be  **identified** and strong consideration given to **educating the family** about the nature of the illness,  enlisting the family’s support, and providing **family therapy**, when indicated.  [II] |
| **Adopt**/  **Refer** | For patients who have experienced a recent **bereavement**, **psychotherapy or antidepressant** treatment  should be used when the reaction to a loss is particularly prolonged or accompanied by significant  psychopathology and functional impairment.  [I] |
| **Refer** | **Support groups** may be helpful for some bereaved individuals.  [III] |
|  | **Co-occurring general medical conditions** |
| **Adopt** | In patients with major depressive disorder, it is important to recognize and address the **potential**  **interplay** between major depressive disorder and any **co-occurring general medical conditions.**  [I] |
| **Adopt** | Communication with **other clinicians** who are providing treatment for general medical conditions is  recommended.  [I] |
| **Adopt** | The clinical assessment should include identifying any **potential interactions** between medications  used to treat depression and those used to treat general medical conditions.  [I] |
| **Adopt** | **Assessment of pain** is also important as it can contribute to and co-occur with depression.  [I] |
| **Adopt** | In addition, the psychiatrist should consider the **effects of prescribed psychotropic medications** on  the patient’s general medical conditions, as well as the effects of interventions for such disorders on  the patient’s psychiatric condition  [I] |
| **Adopt** | In patients with **preexisting hypertension or cardiac conditions**, treatment with specific antidepressant  agents may suggest a need for monitoring of vital signs or cardiac rhythm (e.g., electrocardiogram [ECG]  with TCA treatment; heart rate and blood pressure assessment with SNRIs and TCAs)  [I] |
| **Adopt** | When using antidepressant medications with **anticholinergic side effects**, it is important to consider  the potential for increases in heart rate in individuals with cardiac disease, worsening cognition in  individuals with dementia, development of bladder outlet obstruction in men with prostatic hypertrophy,  and precipitation or worsening of narrow-angle glaucoma.  [I] |
| **Adopt** | Some antidepressant drugs (e.g., bupropion, clomipramine, maprotiline) reduce the **seizure threshold**  and should be used with caution in individuals with preexisting seizure disorders.  [II] |
| **Adopt** | In individuals with **Parkinson’s disease**, the choice of an antidepressant should consider that **serotonergic**  **agents may worsen symptoms** of the disease.  [II] |
| **Adopt** | **Bupropion** has potential **dopamine agonist effects** (benefitting symptoms of Parkinson’s disease but  potentially worsening psychosis)  [II] |
| **Adopt** | In treating the depressive syndrome that commonly occurs following a stroke, consideration should be  given to the potential for **interactions** between **antidepressants** and **anticoagulating** (including antiplatelet)  **medications**.  [I] |
| **Adopt** | Given the health risks associated with obesity and the tendency of some antidepressant medications to  contribute to weight gain, longitudinal **monitoring of weight** (either by direct measurement or patient  report) is recommended.  [I] |
| **Adopt** | Calculation of **body mass index** (BMI)  [II] |
| **Adopt** | If significant increases are noted in the patient’s weight or BMI, the clinician and patient should discuss  potential approaches to **weight control** such as diet, exercise, change in medication, nutrition consultation,  or collaboration with the patient’s primary care physician.  [I] |
| **Adopt** | In patients who have undergone **bariatric surgery** to treat obesity, adjustment of medication formulations  or doses may be required because of **altered medication absorption.**  [I] |
| **Adopt** | For **diabetic patients**, it is useful to collaborate with the **patient’s primary care physician** in monitoring  diabetic control when initiating antidepressant therapy or making significant dosing adjustments.  [II] |
| **Adopt** | Clinicians should be alert to the possibility of **sleep apnea** in patients with depression, particularly those  who present with daytime sleepiness, fatigue, or treatment-resistant symptoms.  [II] |
| **Adopt** | In patients with **known sleep apnea**, treatment choice should **consider the sedative side effects of**  **medication**, with minimally sedating options chosen whenever possible.  [I] |
| **Adopt** | Given the significant numbers of individuals with unrecognized human immunodeficiency virus (HIV)  infection and the availability of effective treatment, consideration should be given to **HIV risk assessment**  **and screening**.  [I] |
| **Adopt** | For patients with HIV infection who are receiving **antiretroviral therapy**, the potential for **drug-drug**  **interactions** needs to be assessed before initiating any psychotropic medications.  [I] |
| **Adopt** | Patients who are being treated with **antiretroviral medications** should be cautioned about **drug-drug**  **interactions with St. John’s wort** that can reduce the effectiveness of HIV treatments.  [I] |
| **Adopt** | In patients with **hepatitis C infection**, **interferon can exacerbate depressive symptoms**, making it  important to monitor patients carefully for worsening depressive symptoms during the course of interferon  treatment.  [I] |
| **Adopt** | Because **tamoxifen** requires active 2D6 enzyme function to be clinically efficacious, patients who receive  tamoxifen for breast cancer or other indications should generally be treated with an **antidepressant** (e.g.,  citalopram, escitalopram, venlafaxine, desvenlafaxine) that has minimal effect on metabolism through the  cytochrome P450 2D6 isoenzyme.  [I] |
| **Adopt** | When depression occurs in the context of **chronic pain**, **SNRIs and TCAs** may be preferable to other  antidepressive agents.  [II] |
| **Refer** | When **ECT** is used to treat major depressive disorder in an individual with a co-occurring general  medical condition, the evaluation should identify **conditions that could require modifications** in ECT  technique (e.g., cardiac conditions, hypertension, central nervous system lesions).  [I] |
| **Adopt** | These should be addressed insofar as possible and discussed with the patient as part of the informed  consent process.  [I] |

**ACRONYMS AND ABBREVIATIONS**

| ECT | Electro convulsive Therapy |
| --- | --- |
| SSRI | Selective Serotonin Reuptake inhibitor |
| SNRI | Selective Norepinephrine Inhibitor |
| TCA | Tricyclic Antidepressant |

**SECTION 6: PANIC DISORDER MANAGEMENT GUIDELINES**

**Panic Disorders Management Guidelines**

**Source Guideline:** Practice Guidelines for the Treatment of Patients with Panic Disorders, 2010, American Psychiatric Association.^7^

**Key to understanding strength of recommendation.**

| Strength of Recommendation | |
| --- | --- |
| [I] | Recommended with substantial clinical confidence |
| [II] | Recommended with moderate clinical confidence |
| [III] | May be recommended on the basis of individual circumstances |

**Table of Recommendations**

|  | **Psychiatric Management** |
| --- | --- |
| **Adopt** | Panic disorder is a common and often disabling mental disorder. Treatment is indicated when symptoms of the disorder interfere with **functioning** or cause **significant distress**.  [I] |
| **Adopt** | Effective treatment for panic disorder should lead not only to a **reduction in frequency and intensity** of panic attacks but also **reductions in anticipatory anxiety and agoraphobic avoidance**, optimally with full remission of symptoms and return to a premorbid level of functioning.  [I]. |
| **Adopt** | Psychiatric management consists of a **comprehensive array of activities and interventions** that should be instituted for all patients with panic disorder, in combination with specific modalities that have demonstrated efficacy.  [I] |
|  | **Establishing a therapeutic alliance** |
| **Adopt** | Psychiatrists should work to establish and maintain a **therapeutic alliance** so that the patient’s care is a collaborative endeavor.  [I] |
| **Adopt** | Careful attention to the **patient’s preferences and concerns** with regard to treatment is essential to fostering a strong alliance.  [I] |
| **Adopt** | In addition, **education** about panic disorder and its treatment should be provided in a language that is readily understandable to the patient.  [I] |
| **Adopt** | Many patients with panic disorder are fearful of certain aspects of treatment (e.g., medication side effects, confronting agoraphobic situations). A **strong therapeutic alliance** is important in supporting the patient through phases of treatment that may be anxiety-provoking.  [I] |
|  | **Performing the psychiatric assessment** |
| **Adopt** | Patients should receive a **thorough diagnostic evaluation** both to establish the diagnosis of panic disorder and to identify other psychiatric or general medical conditions.  [I] |
| **Adopt** | This evaluation generally includes a **history** of the present illness and current symptoms; past psychiatric history; general medical history; history of substance use; personal history (e.g., major life events); social, occupational, and family history; review of the patient’s medications; previous treatments; review of systems; mental status examination; physical examination; and appropriate diagnostic tests (to rule out possible medical causes of panic symptoms) as indicated.  [I] |
| **Adopt** | **Assessment of substance use** should include illicit drugs, prescribed and over-the counter medications, and other substances (e.g., caffeine) that may produce physiological effects that can trigger or exacerbate panic symptoms.  [I] |
| **Adopt** | Delineating the **specific features of panic disorder** that characterize a given patient is an essential element of assessment and treatment planning.  [I] |
| **Adopt** | It is crucial to determine if **agoraphobia** is present and to establish the extent of situational fear and avoidance.  [I] |
| **Adopt** | The psychiatrist also should evaluate **other psychiatric disorders**, as co-occurring conditions may affect the course, treatment, and prognosis of panic disorder.  [I] |
| **Adopt** | It must be determined that panic attacks do not occur solely as a result of a general medical condition or substance use and that they are not better conceptualized as a feature of another diagnosis.  [I] |
| **Adopt** | The presence of medical disorders, substance use, and other psychiatric disorders does not preclude a concomitant diagnosis of panic disorder. If the symptoms of panic disorder are not deemed solely attributable to these factors, then **diagnosing (and treating) both panic disorder and another condition** may be warranted.  [I] |
|  | **Tailoring the treatment plan for the individual patient** |
| **Adopt** | Tailoring the treatment plan to match the **needs** of the particular patient requires a careful assessment of the **frequency and nature** of the patient’s symptoms.  [I] |
| **Adopt** | It may be helpful, in some circumstances, for patients to **monitor their panic symptoms** using techniques such as keeping a daily diary.  [I] |
| **Adopt** | Such monitoring can aid in the **identification of triggers** for panic symptoms, which may become a focus of subsequent intervention. Continuing **evaluation and management** of co-occurring psychiatric and/or medical conditions is also essential to developing a treatment plan for an individual patient.  [I] |
| **Adopt** | Co-occurring conditions may influence both the **selection and implementation of pharmacological and psychosocial treatments** for panic disorder.  [I] |
|  | **Evaluating the safety of the patient** |
| **Adopt** | A careful **assessment of suicide risk** is necessary for all patients with panic disorder.  [I] |
| **Adopt** | Panic disorder has been shown to be associated with an **elevated risk of suicidal ideation and behavior**, even in the absence of co-occurring conditions such as major depression. An **assessment of suicidality** includes the identification of specific psychiatric symptoms known to be associated with suicide attempts or suicide; assessment of past suicidal behavior, family history of suicide and mental illness, current stressors, and potential protective factors such as positive reasons for living; and specific inquiry about suicidal thoughts, intent, plans, means, and behaviors.  [I] |
|  | **Evaluating types and severity of functional impairment** |
| **Adopt** | Panic disorder can impact **numerous spheres of life** including work, school, family, social relationships, and leisure activities. The psychiatrist should develop an understanding of how the panic disorder affects the patient’s functioning in these domains.  [I] |
| **Adopt** | with the aim of developing a treatment plan intended to **minimize impairment**.  [I] |
|  | **Establishing goals for treatment** |
| **Adopt** | All treatments for panic disorder **aim** to reduce the frequency and intensity of panic attacks, anticipatory anxiety, and agoraphobic avoidance, optimally with full remission of symptoms and return to a premorbid level of functioning.  [I] |
| **Adopt** | Treatment of **co-occurring psychiatric disorders** when they are present is an additional goal.  [I] |
| **Adopt** | The **intermediate objectives** that will help achieve these goals will depend on the chosen modality or modalities.  [I] |
|  | **Monitoring the patient’s psychiatric status** |
| **Adopt** | The different elements of panic disorder may resolve at different points during the course of treatment (e.g., panic attacks may remit before agoraphobic avoidance is eliminated). The psychiatrist should continue to **monitor the status of all symptoms** originally presented by the patient.  [I] |
| **Adopt** | Psychiatrists may consider using **rating scales** to help monitor the patient’s status at each session.  [I] |
| **Adopt** | Patients also can be asked to **keep a daily diary** of panic symptoms to aid in ongoing assessment.  [I] |
|  | **Providing education to the patient and, when appropriate, to the family** |
| **Adopt** | Education alone may **relieve some of the symptoms** of panic disorder by helping the patient realize that his or her symptoms are neither life-threatening nor uncommon. Thus, once a diagnosis of panic disorder is made, the patient should be informed of the diagnosis and educated about panic disorder and treatment options.  [I] |
| **Adopt** | Regardless of the treatment modality selected, it is important to inform the patient that in almost all cases the physical sensations that characterize panic attacks are **not acutely dangerous and will abate**.  [I] |
| **Adopt** | **Educational tools** such as books, pamphlets, and trusted web sites can augment the face-to face education provided by the psychiatrist.  [I] |
| **Adopt** | Providing the family with **accurate information** about panic disorder and its treatment is also important for many patients.  [I] |
| **Adopt** | Education sometimes includes a discussion of how changes in the patient’s status affect the **family system** and of how responses of family members can help or hinder treatment of the patient’s panic disorder.  [II] |
| **Adopt** | Patient education also includes the **general promotion of healthy behaviors** such as exercise, good sleep hygiene, and decreased use of caffeine, tobacco, alcohol, and other potentially deleterious substances.  [I] |
|  | **Coordinating the patient’s care with other clinicians** |
| **Adopt** | Many patients with panic disorder will be evaluated by or receive treatment from **other health care professionals** in addition to the psychiatrist. Under such circumstances, the clinicians should communicate periodically to ensure that **care is coordinated** and that treatments are working in synchrony. [I] |
| **Adopt** | It is important to ensure that a **general medical evaluation** has been done (either by the psychiatrist or by another health care professional) to rule out medical causes of panic symptoms.  [I] |
| **Adopt** | **Extensive or specialized testing for medical causes** of panic symptoms is usually not indicated but may be conducted based on the individual characteristics of the patient.  [III] |
|  | **Enhancing treatment adherence** |
| **Adopt** | Problems with treatment adherence can result from a **variety of factors** (e.g., avoidance that is a manifestation of panic disorder, logistical barriers, cultural or language barriers, and problems in the therapeutic relationship). Whenever possible, the psychiatrist should **assess and acknowledge potential barriers to treatment adherence** and should work collaboratively with the patient to minimize their influence.  [I] |
| **Adopt** | Many standard **pharmacological and psychosocial treatments** for panic disorder can be associated with short-term intensification of anxiety (e.g., because of medication side effects or exposure to fear cues during therapy). These temporary increases in anxiety may contribute to decreased treatment adherence. The psychiatrist should adopt a stance that encourages patients to articulate their fears about treatment and should provide patients with a **realistic notion** of what they can expect at different points in treatment.  [I] |
| **Adopt** | In particular, patients should be informed about when a positive response to treatment can be expected so that they do not prematurely abandon treatment due to misconceptions about the **time frame for response**.  [I] |
| **Adopt** | Patients should also be encouraged to **contact the psychiatrist** (e.g., by telephone if between visits) if they have concerns or questions, as these can often be readily addressed and lead to **enhanced treatment adherence**.  [I] |
|  | **Working with the patient to address early signs of relapse** |
| **Adopt** | Although standard treatments effectively reduce the burden of panic disorder for the majority of patients, even some patients with a good treatment response may continue to have **lingering symptoms** (e.g., occasional panic attacks) or have a **recurrence of symptoms** after remission. Patients should be reassured that fluctuations in symptoms can occur during the course of treatment before an acceptable level of remission is reached.  [I] |
| **Adopt** | Patients should also be informed that **symptoms of panic disorder may recur** even after remission and be provided with a plan for how to respond [I]. |
|  | **FORMULATION AND IMPLEMENTATION OF A TREATMENT PLAN**  **1. Choosing a treatment setting** |
| **Adopt** | The treatment of the panic disorder is generally conducted entirely on an **outpatient basis**, as the condition by itself rarely warrants hospitalization.  [I] |
| **Refer** | However, it may be necessary to **hospitalize a patient** with the panic disorder because of symptoms of co-occurring disorders (e.g., when acute suicidality associated with a mood disorder is present or when inpatient detoxification is required for a substance use disorder).  [I] |
| **Refer** | Under such circumstances, the **treatment of panic disorder can be initiated in the hospital** along with treatment of the disorder that prompted hospitalization.  [I] |
| **Refer** | **Rarely**, **hospitalization or partial hospitalization** is required in very severe cases of panic disorder with agoraphobia when the administration of outpatient treatment has been ineffective or is impractical.  [I] |
| **Adopt** | **Home visits** are another treatment option for patients with severe agoraphobia who are limited in their ability to travel or leave the house.  [II] |
| **Adopt** | When accessibility to mental health care is limited (e.g., in remote or underserved areas), **telephone- or Internet-based treatments** may be considered.  [II] |
|  | **Choosing an initial treatment modality** |
| **Adopt/**  **Refer** | A range of specific **psychosocial and pharmacological interventions** have proven benefits in treating panic disorder. The use of a selective serotonin reuptake inhibitor (SSRI), c (SNRI), tricyclic antidepressant (TCA), benzodiazepine (appropriate as monotherapy only in the absence of a co-occurring mood disorder), or cognitive-behavioral therapy (CBT) as the initial treatment for panic disorder is strongly supported by demonstrated efficacy in numerous randomized controlled trials.  [I] |
| **Adopt/Refer** | A particular form of **psychodynamic psychotherapy, panic-focused psychodynamic psychotherapy** (PFPP), was effective and could be offered as an initial treatment under certain circumstances.  [II] |
| **Adopt** | There is insufficient evidence to recommend any of these pharmacological or psychosocial interventions as superior to the others, or to routinely recommend a combination of treatments over monotherapy.  [II] |
| **Adopt** | Although **combination treatment does not appear to be significantly superior** to standard monotherapy as initial treatment for most individuals with panic disorder, psychiatrists and patients may choose this option based on individual circumstances (e.g., patient preference).  [II] |
| **Adopt** | **Considerations that guide the choice** of an initial treatment modality include patient preference, the risks and benefits for the particular patient, the patient’s past treatment history, the presence of co-occurring general medical and other psychiatric conditions, cost, and treatment availability.  [I] |
| **Adopt/Refer** | **Psychosocial treatment** (with the strongest evidence available for CBT) is recommended for patients who prefer non-medication treatment and can invest the time and effort required to attend weekly sessions and complete between-session practices.  [I] |
| **Adopt** | One caveat is that CBT and other specialized psychosocial treatments are **not readily available** in some geographic areas. Pharmacotherapy (usually with an SSRI or SNRI) is recommended for patients who prefer this modality or who do not have sufficient time or other resources to engage in psychosocial treatment.  [I] |
| **Adopt/Refer** | **Combined treatment** should be considered for patients who have failed to respond to standard monotherapies and may also be used under certain clinical circumstances (e.g., using pharmacotherapy for temporary control of severe symptoms that are impeding the patient’s ability to engage in psychosocial treatment).  [II] |
| **Adopt/Refer** | Adding psychosocial treatment to pharmacotherapy either from the start, or at some later point in treatment, may **enhance long-term outcomes** by reducing the likelihood of relapse when pharmacological treatment is stopped.  [II] |
|  | **Evaluating whether the treatment is working** |
| **Adopt** | After treatment is initiated, it is **important to monitor changes** in key symptoms such as frequency and intensity of panic attacks, level of anticipatory anxiety, degree of agoraphobic avoidance, and severity of interference and distress related to panic disorder.  [I] |
| **Adopt** | Effective treatment should produce a decrease in each of these domains, although some may change more quickly than others. The severity of co-occurring conditions also should be assessed at regular intervals, as treatment of panic disorder can influence co-occurring conditions (e.g., major depression; other anxiety disorders)  [I] |
| **Adopt** | **Rating scales** are a useful adjunct to ongoing clinical assessment for the purpose of evaluating treatment outcome.  [I] |
|  | **Determining if and when to change treatment** |
| **Adopt** | Some individuals do not respond, or respond incompletely, to first-line treatments for panic disorder. Whenever treatment response is unsatisfactory, the psychiatrist should first consider the possible contribution of **fundamental clinical factors** such as an underlying untreated medical illness that  accounts for the symptoms, interference by co-occurring general medical or psychiatric conditions (including depression and substance use), inadequate treatment adherence, problems in the therapeutic alliance, the presence of psychosocial stressors, motivational factors, and inability to tolerate a particular treatment.  [I] |
| **Adopt** | These **potential impediments to successful treatment** should be addressed as early as possible in treatment.  [I] |
| **Adopt** | In addition, if **panic-related concerns** are leading the patient to minimize the impact of avoidance or accept functional limitations, the patient should be encouraged to think through the costs and benefits of accepting versus treating functional limitations.  [I] |
| **Adopt** | Clinicians should be reluctant to accept **partial improvement** as a satisfactory outcome and should **aim for remission** whenever feasible.  [I] |
| **Adopt** | If response to treatment remains unsatisfactory, and if an adequate trial has been attempted, it is appropriate for the psychiatrist and the patient to **consider a change**.  [I] |
| **Adopt** | Decisions about whether and how to make changes will depend on the **level of response** to the initial treatment (i.e., none versus partial), the palatability and feasibility of other treatment options for a given patient, and the level of symptoms and impairment that remain.  [I] |
| **Adopt** | **Persistent significant symptoms** of panic disorder despite a lengthy course of a particular treatment should **trigger a reassessment** of the treatment plan, including possible consultation with another qualified professional.  [I] |
|  | **Approaches to try when first-line treatment is unsuccessful** |
| **Adopt/**  **Refer** | If fundamental clinical issues have been addressed and it is determined that a change is desirable, the psychiatrist and patient can either augment the current treatment by **adding another agent** (in the case of pharmacotherapy) or **another modality** (i.e., add CBT if the patient is already receiving pharmacotherapy, or add pharmacotherapy if the patient is already receiving CBT).  [I] |
| **Adopt/Refer** | or they can decide to **switch to a different medication or therapeutic modality**.  [I] |
| **Adopt** | Decisions about how to address treatment resistance are usually **highly individualized and based on clinical judgment**, since few studies have tested the effects of specific switching or augmentation strategies. However, augmentation is generally a reasonable approach if some significant benefits were observed with the original treatment.  [II] |
| **Adopt** | On the other hand, if the original treatment failed to provide any significant alleviation of the patient’s symptoms, a **switch in treatment** may be more useful.  [II] |
| **Adopt** | If one first-line treatment (e.g., CBT, an SSRI, an (SNRI) has failed, **adding or switching to another first-line treatment** is recommended.  [I] |
| **Adopt** | Adding a **benzodiazepine to an antidepressant** is a common augmentation strategy to target residual symptoms.  [II] |
| **Adopt** | If the treatment options with the most robust evidence have been unsuccessful, other options with some **empirical support** can be considered (e.g., a monoamine oxidase inhibitor [MAOI], PFPP).  [II] |
| **Adopt** | After first- and second-line treatments and augmentation strategies have been exhausted (either due to lack of efficacy or intolerance of the treatment by the patient), **less well-supported treatment strategies** may be considered.  [III] |
| **Adopt/**  **Refer** | These include **monotherapy or augmentation with gabapentin or a second-generation antipsychotic or with a psychotherapeutic intervention** other than CBT or PFPP.  [III] |
| **Adopt** | Psychiatrists are encouraged to **seek consultation from experienced colleagues** when developing treatment plans for patients whose symptoms have been resistant to standard treatments for panic disorder.  [I] |
|  | **Specific psychosocial interventions** |
| **Adopt/**  **Refer** | Psychosocial treatments for panic disorder should be **conducted by professionals** with an appropriate level of training and experience in the relevant approach.  [I] |
| **Adopt/**  **Refer** | Based on the current available evidence, **CBT** is the psychosocial treatment that would be indicated most often for patients presenting with panic disorder.  [I] |
| **Adopt/**  **Refer** | **Cognitive-behavioral therapy is a time-limited treatment** (generally 10–15 weekly sessions) with durable effects. It can be successfully administered individually or in a group format.  [I] |
| **Adopt/**  **Refer** | Self-directed forms of CBT may be useful for patients who do not have ready access to a trained CBT therapist.  [II] |
| **Adopt/**  **Refer** | Cognitive-behavioral therapy for panic disorder generally includes psychoeducation, self-monitoring, countering anxious beliefs, exposure to fear cues, modification of anxiety-maintaining behaviors, and relapse prevention.  [I] |
| **Adopt/**  **Refer** | **Exposure therapy**, which focuses almost exclusively on systematic exposure to fear cues, is also effective. [I] |
| **Adopt/**  **Refer** | **Panic-focused psychodynamic psychotherapy** also has demonstrated efficacy for panic disorder, although its evidence base is more limited. Panic-focused psychodynamic psychotherapy may be indicated as an initial psychosocial treatment in some cases (e.g., patient preference).  [II] |
| **Adopt/**  **Refer** | Panic-focused psychodynamic psychotherapy is a **time-limited treatment** (twice weekly for 12 weeks) that is administered on an individual basis. Panic-focused psychodynamic psychotherapy utilizes the general principles of psychodynamic psychotherapy, with a **special focus on the transference** as the therapeutic agent promoting change, and encourages patients to confront the emotional significance of their panic symptoms with the aim of promoting greater autonomy, symptom relief, and improved functioning. Although psychodynamic psychotherapies (other than PFPP) that focus more broadly on emotional and interpersonal issues have not been formally tested for panic disorder, some case report data and clinical experience suggest this approach may be useful for some patients.  [III] |
| **Adopt/**  **Refer** | Other psychosocial treatments have not been formally tested for panic disorder or have proven ineffective (e.g.eye movement desensitization and reprocessing [EMDR]) or inferior to standard treatments such as CBT (e.g., supportive psychotherapy). **Group CBT** is effective and can be recommended for the treatment of panic disorder.  [I] |
| **Refer** | **Other group therapies** (including patient support groups) are not recommended as monotherapies for panic disorder, although they may be **useful adjuncts** to other effective treatments for some patients.  [III] |
| **Adopt** | **Couples or family therapy alone is not recommended** as a treatment for panic disorder, although it may be helpful in addressing co-occurring relationship dysfunction.  [III] |
| **Adopt/**  **Refer** | It can be **beneficial to include significant others in CBT** (e.g., partner-assisted exposure therapy for agoraphobia), especially if they are educated in the cognitive-behavioral model of panic disorder and enlisted to help with between-session practices.  [II] |
| **Adopt** | When pursuing other treatments for panic disorder (e.g., pharmacotherapy), **education of significant others** about the nature of the disorder and enlisting significant others to improve treatment adherence may also be helpful.  [III] |
|  | **Specific pharmacological interventions** |
| **Adopt** | **Selective serotonin reuptake inhibitors, SNRIs, TCAs, and benzodiazepines** have demonstrated efficacy in numerous controlled trials and are recommended for the treatment of panic disorder.  [I] |
| **Adopt** | **Monoamine oxidase inhibitors** appear effective for panic disorder but, because of their safety profile, they are generally **reserved for patients who have failed to respond** to several first-line treatments.  [II] |
| **Adopt** | **Other medications** with less empirical support (e.g., mirtazapine, anticonvulsants such as gabapentin) may be **considered as monotherapy** or **adjunctive treatments** for panic disorder when patients have **failed to respond** to several standard treatments or based on other individual circumstances.  [III] |
| **Adopt** | Because SSRIs, SNRIs, TCAs, and benzodiazepines **appear roughly comparable in their efficacy** for panic disorder, selecting a medication for a particular patient mainly involves considerations of side effects (including any applicable warnings from the U.S. Food and Drug Administration [FDA]), cost, pharmacological properties, potential drug interactions, prior treatment history, co-occurring general medical and psychiatric conditions, and the strength of the evidence base for the particular medication  in the treatment of panic disorder.  [I] |
| **Adopt** | The relatively favorable safety and side effect profile of **SSRIs and SNRIs** makes them the **best initial choice** for many patients with panic disorder.  [I] |
| **Adopt** | Although **TCAs are effective**, the **side effects and greater toxicity** in overdose associated with them often limit their acceptability to patients and their clinical utility. Selective serotonin reuptake inhibitors, SNRIs, and TCAs are all preferable to benzodiazepines as monotherapies for patients with co-occurring depression or substance use disorders.  [I] |
| **Adopt** | **Benzodiazepines** may be especially **useful adjunctively with antidepressants** to treat residual anxiety symptoms.  [II] |
| **Adopt** | **Benzodiazepines** may be preferred (as monotherapies or in combination with antidepressants) for patients with **very distressing or impairing symptoms** in whom rapid symptom control is critical.  [II] |
| **Adopt** | The benefit of **more rapid response to benzodiazepines** must be balanced against the possibilities of **troublesome side effects** (e.g., sedation) and **physiological dependence** that may lead to difficulty discontinuing the medication.  [I] |
| **Adopt** | Patients should be educated about the **likely time course of treatment effects** associated with a particular medication.  [I] |
| **Adopt** | Because patients with panic disorder can be sensitive to medication side effects, **low starting doses** of SSRIs, SNRIs, and TCAs (approximately half of the starting doses given to depressed patients) are recommended.  [I] |
| **Adopt** | The low dose is maintained for several days then **gradually increased to a full therapeutic dose** over subsequent days and as tolerated by the patient.  [I] |
| **Adopt** | **Underdosing of antidepressants** (i.e., starting low and then not increasing gradually to full therapeutic dosages as needed) is common in treatment of panic disorder and is a frequent source of partial response or nonresponse.  [II] |
| **Adopt** | A **regular dosing schedule** rather than a p.r.n. (“as needed”) schedule is preferred for patients with panic disorder who are taking benzodiazepines,  [II] |
| **Adopt** | where the goal is to prevent panic attacks rather than reduce symptoms once an attack has already occurred. Once an initial pharmacotherapy has been selected, patients are **typically seen every 1–2 weeks** when first starting a new medication, **then every 2–4 weeks** until the dose is stabilized.  [I] |
| **Adopt** | After the dose is stabilized and symptoms have decreased, patients will most likely require **less frequent visits.**  [I] |
| **Adopt** | When considering any specific medication, the psychiatrist must **balance the risks** associated with the medication **against the clinical need** for pharmacotherapy.  [I] |
| **Adopt** | The FDA has warned of the possibility that **antidepressants may increase the risk of suicidal ideation** and behavior in patients age 25 years and younger; this is an important factor to consider before using an SSRI, an SNRI, or a TCA for panic disorder. Other important safety considerations for SSRIs include the possible increased likelihood of upper gastrointestinal bleeding (particularly when taken in combination with non-steroid anti-inflammatory drugs [NSAIDs] or with aspirin) and increased risk of falls and osteoporotic fractures in patients age 50 years and older. With venlafaxine extended-release (ER), a small proportion of patients may develop sustained hypertension. It is recommended that psychiatrists  assess blood pressure during treatment, particularly when venlafaxine ER is titrated to higher doses.  [I] |
| **Adopt** | **Tricyclic antidepressants** should not be prescribed for patients with panic disorder who also have acute **narrow-angle glaucoma** or clinically significant **prostatic hypertrophy**. Tricyclic antidepressants may increase the likelihood of **falls**, particularly among elderly patients. A **baseline electrocardiogram** should be considered before initiating a TCA, because patients with preexisting cardiac conduction abnormalities may experience significant or fatal arrhythmia with TCA treatment. Overdoses with TCAs can lead to **significant cardiac toxicity and fatality**, and therefore TCAs should be used judiciously in suicidal patients. **Benzodiazepines** may produce **sedation, fatigue, ataxia, slurred speech, memory impairment, and weakness**. Geriatric patients taking benzodiazepines may be at **higher risk for falls and fractures**. Because of an **increased risk of motor vehicle accidents** with benzodiazepine use, patients should be warned about driving or operating heavy machinery while taking benzodiazepines.  [I] |
| **Adopt** | Patients should also be advised about the **additive effects of benzodiazepines and alcohol**.  [I] |
| **Adopt** | **Caution and careful monitoring** are indicated when prescribing benzodiazepines to elderly patients, those with preexisting cognitive impairment, or those with a history of substance use disorder.  [I] |
| **Adopt** | For women with panic disorder who are **pregnant, nursing, or planning to become pregnant, psychosocial interventions** should be considered in lieu of pharmacotherapy.  [II] |
| **Adopt** | **Pharmacotherapy** may also be indicated.  [III] |
| **Adopt** | But requires weighing and discussion of the potential benefits and risks with the patient, her **obstetrician**, and, whenever possible, her partner.  [I] |
| **Adopt** | Such discussions should also consider the **potential risks to the patient and the child** of untreated psychiatric illness, including panic disorder and any co-occurring psychiatric conditions.  [I] |
|  | **MAINTAINING OR DISCONTINUING TREATMENT AFTER RESPONSE** |
| **Adopt** | Pharmacotherapy should generally be continued for **1 year or more after acute response** to promote further symptom reduction and decrease risk of recurrence.  [I] |
| **Adopt** | Incorporating **maintenance treatment** (e.g., monthly “booster” sessions focused on relapse prevention) into psychosocial treatments for panic disorder also may help maintain positive response,  [II] |
| **Adopt** | although more systematic investigation of this issue is needed. Before **advising a taper of effective pharmacotherapy**, the psychiatrist should consider several factors, including the duration of the patient’s symptom stability, the presence of current or impending psychosocial stressors in the patient’s life, and the extent to which the patient is motivated to discontinue the medication.  [II] |
| **Adopt** | **Discussion of medication taper** should also include the possible outcomes of taper, which could include discontinuation symptoms and recurrence of panic symptoms.  [I] |
| **Adopt** | If medication is tapered, it should be done in a **collaborative manner** with continual assessment of the effects of the taper and the patient’s responses to any changes that emerge.  [I] |
| **Adopt** | If a decision is made to discontinue successful treatment with an SSRI, an SNRI, or a TCA, the medication should be **gradually tapered** (e.g., one dosage step down every month or two), thereby providing the opportunity to watch for recurrence and, if desired, to reinitiate treatment at a previously effective dose.  [II] |
| **Adopt** | However, under **more urgent conditions** (e.g., the patient is pregnant, and the decision is made to discontinue medications immediately), these medications **can be discontinued much more quickly**  [I] |
| **Adopt** | The approach to **benzodiazepine discontinuation** also involves a **slow and gradual tapering of dose**.  [I] |
| **Adopt** | **Withdrawal symptoms and symptomatic rebound** are commonly seen with benzodiazepine discontinuation, can occur throughout the taper, and may be especially severe toward the end of the taper. This argues for tapering benzodiazepines very slowly for patients with panic disorder, probably **over 2–4 months** and at rates no higher than 10% of the dose per week.  [I] |
| **Adopt**/  **Refer** | **Cognitive-behavioral therapy** may be added to **facilitate withdrawal** from benzodiazepines.  [I] |

**ACRONYMS & ABBREVIATIONS**

| CBC | Complete Blood Count | NSAIDs | Non-steroidal Anti-inflammatory drugs |
| --- | --- | --- | --- |
| CBT | Cognitive Behavioral Therapy | p.r.n | As needed |
| ECG | Electrocardiogram | PFPP | Planned Focused Psychodynamic Psychotherapy |
| ER | Extended-Release | SSRI | selective serotonin reuptake inhibitor |
| EMDR | Eye Movement Desensitization and Reprocessing | SNRI | selective serotonin reuptake inhibitor |
| FDA | Food & Drug Administration | TCA | tricyclic antidepressant |
| MAOI | monoamine oxidase inhibitor | TSH | Thyroid Stimulating Hormone |

**SECTION 7: DELIRIUM**

**DELIRIUM MANAGEMENT**

**Source guideline:** Practice Guideline for the Treatment of Patients with Delirium, The American Psychiatric Association (APA) Practice Guidelines 2010.^8^

**Key to understanding strength of recommendation.**

| Strength of Recommendation | |
| --- | --- |
| [I] | Recommended with substantial clinical confidence |
| [II] | Recommended with moderate clinical confidence |
| [III] | May be recommended based on individual circumstances |

**Table of Recommendations**

|  | **Recommendations** |
| --- | --- |
| **Adopt** | Delirium is primarily a disturbance of **consciousness, attention, cognition, and perception** but can also affect sleep, psychomotor activity, and emotions. |
|  | **Psychiatric Management** |
| **Adopt** | Psychiatric management is an **essential feature of treatment** for delirium and should be implemented for all patients with delirium.  [I] |
| **Adopt** | The **specific tasks** that constitute psychiatric management include the following: **coordinating the care** of the patient with other clinicians; identifying the **underlying cause**(s) of the delirium; initiating immediate **interventions** for urgent general medical conditions; providing **treatments** that address the underlying etiology of the delirium; assessing and ensuring the **safety** of the patient and others; assessing the patient’s psychiatric status and **monitoring** it on an ongoing basis; assessing individual and family **psychological and social**  characteristics; establishing and maintaining a **supportive therapeutic stance** with the patient, the family, and other clinicians; **educating** the patient, family, and other clinicians regarding the illness; and providing **post-delirium management** to support the patient and family and providing education regarding risk factors for future episodes. |
|  | **Environmental and supportive interventions** |
| **Adopt** | These interventions are generally recommended for all patients with delirium.  [I]. |
| **Adopt** | **Environmental interventions** are designed to reduce or eliminate environmental factors that exacerbate delirium. They include providing an optimal level of environmental stimulation, reducing sensory impairments, making environments more familiar, and providing environmental cues that facilitate orientation. Cognitive-emotional supportive measures include providing patients with reorientation, reassurance, and information concerning delirium that may reduce fear or demoralization. In addition to providing such supportive interventions themselves,it may be helpful for psychiatrists to inform nursing staff, general medical physicians, and family members of  their importance. |
|  | **Somatic interventions** |
| **Adopt** | The choice of somatic interventions for delirium will depend on the **specific features** of a patient’s clinical condition, the **underlying etiology** of the delirium, and any **associated comorbid conditions**.  [I] |
| **Adopt** | **Antipsychotic medications** are often the pharmacologic treatment of choice.  [I] |
| **Adopt** | **Benzodiazepine treatment** as a monotherapy is generally reserved for delirium caused by withdrawal of alcohol or sedative-hypnotics.  [I] |
| **Adopt** | Patients with delirium who can tolerate only lower doses of antipsychotic medications may benefit from the **combination of a benzodiazepine and antipsychotic medication**.  [III] |
| **Adopt** | Other **somatic interventions** may be considered for patients with delirium who have particular clinical conditions or specific underlying etiologies. Cholinergics such as physostigmine may be useful in delirium known to be caused specifically by anticholinergic medications.  [II] |
| **Refer** | **Paralysis, sedation, and mechanical ventilation** may be required for agitated patients with delirium and hypercatabolic conditions.  [III] |
| **Adopt** | **Palliative treatment** with opiates may be needed by patients with delirium for whom pain is an aggravating factor.  [III] |
| **Adopt** | **Multivitamin replacement** should be given to patients with delirium for whom there is the possibility of B vitamin deficiencies (e.g., those who are alcoholic or malnourished).  [II] |

**ACRONYMS & ABBREVIATIONS**

| BUN | Blood Urea Nitrogen |
| --- | --- |
| CBC | Complete Blood Count |
| CT | Computed Tomography |
| MRI | Magnetic Resonance Imaging |
| ECG | Electrocardiogram |

**SECTION 8: DEMENTIA MANAGEMENT**

**TREATMENT OF PATIENTS WITH** **DEMENTIA & ALZHEIMER DISEASE**

**Source guidelines:**

**-**Practice Guideline for the Treatment of Patients With Alzheimer’s Disease and Other Dementias Second Edition, 2007.^9^

-EFNS guidelines for the diagnosis and management of Alzheimer’s Disease. European Journal of Neurology 2010.^10^

**Key to understanding level of evidence and strength of recommendation.**

| Grades of Recommendation | |
| --- | --- |
| [I] | Recommended with substantial clinical confidence |
| [II] | Recommended with moderate clinical confidence |
| [III] | May be recommended on the basis of individual circumstances |

| Strength of evidence | |
| --- | --- |
| Level A | Established as effective, ineffective, or harmful requires at least one convincing class I study or at least two consistent, convincing class II studies |
| Level B | Established as probably useful/predictive or not useful/predictive requires at least one convincing class II study or overwhelming class III evidence |
| Level C | Established as possibly useful/predictive or not useful/predictive requires at least two convincing class III studies |
| Good practice points | |

**Table of Recommendations**

|  | **Diagnosis and Assessment** |
| --- | --- |
| **Adopt** | Patients with dementia display a broad range of **cognitive impairments** and **neuropsychiatric symptoms** that can cause significant distress to themselves and caregivers. As a result, **individualized and multimodal treatment** plans are required.  [I] |
| **Adopt** | Dementia is usually **progressive**, and **treatment must evolve** with time in order to address newly emerging issues.  [I] |
| **Adopt** | Clinical history should be supplemented by an informant.  [Level A]. |
| **Adopt** | A neurological and physical examination should be performed in all patients with dementia  [good practice point]. |
| **Adopt** | ADL impairment due to cognitive decline is an essential part of the diagnostic criteria for dementia and should be assessed in the diagnostic evaluation  [Level A]. |
| **Adopt** | Cognitive assessment should be performed in all patients  [Level A]. |
| **Adopt** | Quantitative neuropsychological testing should be made in patients with questionable or  very early AD  [Level B]. |
| **Adopt** | The assessment of cognitive functions should include a general cognitive measure and more detailed testing of the main cognitive domains, and in particular an assessment of delayed  Recall  [Level A]. |
| **Adopt** | Memory, especially episodic memory, should be systematically assessed.  [I]. |
| **Adopt** | In patients with moderate memory impairment cued recall could be more appropriate than  free recall  [Level B]. |
| **Adopt** | CT and MRI may be used to exclude treatable causes of dementia. Multislice CT and coronal MRI may be used to assess hippocampal atrophy to support a clinical diagnosis of AD  [Level B]. |
| **Adopt** | FDG PET and perfusion SPECT are useful adjuncts when diagnosis remains in doubt  [Level B]. |
| **Adopt** | Dopaminergic SPECT is useful to differentiate AD from DLB  [Level A]. |
| **Adopt** | Follow up with serial MRI is useful in a clinical setting to document disease progression  (good practice point). |
| **Adopt** | The Rey Auditory Verbal Learning Test (RAVLT) can distinguish between patients with AD  and those without dementia or between AD and other forms of dementia with a diagnostic accuracy of 83–86%. In particular, a very severe impairment (0 score) on RAVLT delayed free recall has a very high (97%) specificity for AD  [I]. |
| **Adopt** | At each stage the **psychiatrist should be vigilant** for symptoms likely to be present, should identify and treat co-occurring psychiatric and medical conditions, and should help patients and families anticipate future symptoms and the care likely to be required.  [I] |
|  | **Treatment of Cognitive Symptoms** |
| **Adopt** | **Three cholinesterase inhibitors**—donepezil, rivastigmine, and galantamine—are approved by the U.S. Food and Drug Administration (FDA) for treatment of mild to moderate Alzheimer’s disease, and donepezil has been approved by the FDA for severe Alzheimer’s disease. These medications have similar rates of adverse effects and have been shown to lead to modest benefits in a substantial minority of patients (i.e., 30%–40% in clinical trials). These medications should be offered to patients with mild to moderate Alzheimer’s disease after a thorough discussion of their potential risks and benefits,  [I] |
| **Adopt** | they may be helpful for patients with severe Alzheimer’s disease.  [II] |
| **Adopt** | **Cholinesterase inhibitors** should be considered for patients with **mild to moderate dementia** associated with Parkinson’s disease.  [I] |
| **Adopt** | **Cholinesterase inhibitors** can be considered for patients with **dementia with Lewy bodies.**  [II] |
| **Adopt** | The constructs of **mild cognitive impairment and vascular dementia** are evolving and have **ambiguous boundaries with Alzheimer’s disease**. The efficacy and safety of cholinesterase inhibitors for patients with these disorders are uncertain; therefore, no specific recommendation can be made at this time, although individual patients may benefit from these agents.  [II] |
| **Adopt** | **Memantine**, a non-competitive N-methyl-D-aspartate (NMDA) antagonist, which has been approved by the FDA for use in patients with moderate and severe Alzheimer’s disease, may provide **modest benefits and has few adverse effects**; thus, it may be considered for such patients.  [I] |
| **Adopt** | There is some evidence of its benefit in mild Alzheimer’s disease.  [III] |
| **Adopt** | very limited evidence of its benefit in vascular dementia.  [I] |
| **Adopt** | **Vitamin E (α-tocopherol) is no longer recommended** for the treatment of cognitive symptoms of dementia because of limited evidence for its efficacy as well as safety concerns.  [II] |
| **Adopt** | **Nonsteroidal anti-inflammatory agents (NSAIDs), statin medications, and estrogen supplementatio**n (with conjugated equine estrogens) have shown a **lack of efficacy and safety** in placebo-controlled trials in patients with Alzheimer’s disease and therefore are not recommended. [I] |
|  | **Psychiatry Management** |
| **Adopt** | The treatment of patients with dementia should be based on a **thorough psychiatric, neurological, and general medical evaluation** of the nature and cause of the cognitive deficits and associated noncognitive symptoms, in the context of a solid alliance with the patient and family.  [I] |
| **Adopt** | It is particularly critical to **identify and treat general medical conditions**, most notably delirium, that may be responsible for or contribute to the dementia or associated neuropsychiatric symptoms.  [I] |
| **Adopt** | Ongoing assessment includes **periodic monitoring** of the development and evolution of cognitive and noncognitive psychiatric symptoms and their response to intervention.  [I] |
| **Adopt** | In order to offer prompt treatment, enhance safety, and provide timely advice to the patient and family, it is generally necessary to see patients in **routine follow-up** at least **every 3–6 months.**  [II] |
| **Adopt/**  **Refer** | More **frequent visits** (e.g., up to once or twice a week) or even psychiatric hospitalization may be required for patients with **acute, complex, or potentially dangerous symptoms** or for the administration of specific therapies.  [I] |
| **Adopt** | **Recommended assessments** include evaluation of suicidality, dangerousness to self and others, and the potential for aggression, as well as evaluation of living conditions, safety of the environment, adequacy of supervision, and evidence of neglect or abuse.  [I] |
| **Adopt** | All patients and families should be informed that even **mild dementia** increases the risk of **vehicular accidents.**  [I] |
| **Adopt** | **Mildly impaired patients** should be advised to limit their driving to **safer situations or to stop driving.**  [I] |
| **Adopt** | **Moderately impaired patients** should be instructed **not to drive.**  [I] |
| **Adopt** | Advice about driving cessation should also be **communicated to family members**, as the implementation of the recommendation often falls on them.  [I] |
| **Adopt** | Relevant state laws regarding notification should be followed.  [I] |
| **Adopt** | Important aspects of psychiatric management include **educating patients and families** about the illness, its treatment, and sources of additional care and support (e.g., support groups, respite care, nursing homes, and other long-term-care facilities) and advising patients and their families of the need for financial and legal planning due to the patient’s eventual incapacity (e.g., power of attorney for medical and financial decisions, an up-to-date will, and the cost of long-term care).  [I] |
|  | **Specific Psychotherapies and Other Psychosocial Treatments** |
| **Adopt/**  **Refer** | In addition to the general psychosocial interventions subsumed under psychiatric management, a number of specific interventions are appropriate for some patients.  **Behaviour oriented treatments** are used to identify the antecedents and consequences of problem behaviours and attempt to reduce the frequency of behaviours by directing changes in the environment that alter these antecedents and consequences.  [II] |
| **Adopt/**  **Refer** | **Stimulation-oriented treatments**, such as recreational activity, art therapy, music therapy, and pet therapy, along with other formal and informal means of maximizing pleasurable activities for patients, have modest support from clinical trials for improving behaviour, mood, and, to a lesser extent, function, and common sense supports their use as part of the humane care of patients.  [II] |
| **Adopt/**  **Refer** | Among the **emotion-oriented treatments**, supportive psychotherapy can be employed to address issues of loss in the early stages of dementia.  [II] |
| **Adopt/**  **Refer** | **Reminiscence therapy** has some modest research support for improvement of mood and behaviour.  [III] |
| **Adopt/**  **Refer** | **validation therapy** and sensory integration have less research support.  [III] |
|  | **Special Concerns Regarding Somatic Treatments for Elderly Patients and Patients With Dementia** |
| **Adopt** | Medications are effective in the management of some symptoms associated with dementia, but they must be used with caution in this patient population.  [I] |
| **Adopt** | Because **age** may **alter the absorption, distribution, metabolism, and elimination** of many medications, elderly individuals may be more sensitive to their effects. General medical conditions and use of more than one medication may further affect the pharmacokinetics of many medications. In addition, patients with dementia may be more likely to experience certain medication adverse effects, including anticholinergic effects, orthostasis, sedation, and parkinsonism. Finally, symptoms of dementia **may alter medication adherence** in ways that are unsafe. Consequently, when using pharmacotherapy in patients with dementia, **low starting doses**, **small increases in dose**, and **long intervals** between dose increments may be needed, in addition to ensuring that a system is in place that can enhance proper medication adherence.  [I] |
|  | **Treatment of Psychosis and Agitation** |
| **Adopt** | Psychosis, aggression, and agitation are **common in patients** with dementia and may respond to similar therapies. When deciding if treatment is indicated, it is critical to consider the safety of the patient and those around him or her.  [I] |
| **Adopt** | A careful **evaluation** for **general medical, psychiatric, environmental, or psychosocial problems** that may underlie the disturbance should be undertaken.  [I] |
| **Adopt** | If possible and safe, such **underlying causes** should be **treated first.**  [I] |
| **Adopt** | If this does not resolve the symptoms, and if they do not cause significant danger or distress to the patient or others, such symptoms are best treated with **environmental measures**, including **reassurance and redirection.**  [I] |
| **Adopt** | For **agitation**, some of the **behavioural measures** (stimulation-oriented treatment, emotion-oriented treatment) may also be **helpful.**  [II] |
| **Adopt** | If these measures are unsuccessful or the behaviours are particularly dangerous or distressing, then the symptoms may be treated judiciously with one of the agents discussed in the following paragraphs.  [II] |
| **Adopt** | The use of such agents should be re-evaluated and their benefit documented on an ongoing basis. [I] |
| **Adopt** | On the basis of good evidence, **antipsychotic medications** are recommended for the treatment of **psychosis** in patients with dementia.  [II] |
| **Adopt** | And for the treatment of **agitation.**  [II] |
| **Adopt** | These medications have also been shown to provide **modest improvement in behavioural symptoms** in general.  [I] |
| **Adopt** | Evidence for the efficacy of these agents is based mostly on **6–12-week trials** in nursing home residents and outpatients. There is limited research on their use beyond 12 weeks, but considerable clinical experience supports this practice.  [II] |
| **Adopt** | Evidence for a difference in efficacy and safety among antipsychotic medications is limited. Antipsychotic medications as a group are associated with **a number of severe adverse events**, including increased risks for death, cerebrovascular accidents, tardive dyskinesia, neuroleptic malignant syndrome, hyperlipidemia, weight gain, diabetes mellitus, sedation, parkinsonism, and worsening of cognition. Thus, they must be **used with caution and at the lowest effective dosage.**  [I] |
| **Adopt** | after considering the **risks of not treating** the psychiatric symptoms.  [I] |
| **Adopt** | **Patients and families** should be **advised** about **potential benefits and risks** of antipsychotic agents, particularly the risk of mortality.  [I] |
| **Adopt** | **Second-generation (atypical) antipsychotics** currently have a **black box warning** for increased risk of mortality in elderly patients; recent data suggest that first-generation (typical) agents carry at least a similar risk. **High-potency agents** tend to cause **akathisia and parkinsonian symptoms**; **low-potency agents** tend to cause **sedation, confusion, delirium, postural hypotension, and peripheral anticholinergic effects**. The decision of which antipsychotic to use is based on the relationship between the side-effect profile and the characteristics of the individual patient.  [I] |
| **Adopt** | Data demonstrating benefit from **benzodiazepines** are modest, but benzodiazepines occasionally have a role in treating patients with prominent **anxiety.**  [III] |
| **Adopt** | or on an as-needed basis for patients with infrequent episodes of agitation or for those who require sedation for a procedure such as a tooth extraction or a diagnostic examination.  [II] |
| **Adopt** | Adverse effects of benzodiazepines include sedation, worsening cognition, delirium, increased risk of falls, and worsening of breathing disorders. Lorazepam and oxazepam, which have no active metabolites, are preferable to agents with a longer half-life such as diazepam or clonazepam.  [III] |
| **Adopt** | There is **minimal evidence** for the **efficacy** of **anticonvulsants**, **lithium**, and **beta-blockers** for the treatment of psychosis or agitation in dementia, and these medications have significant adverse effects; therefore, they are generally not recommended except for patients for whom other treatments have failed.  [III] |
| **Adopt** | The **antidepressant** **trazodone** and the **selective serotonin reuptake inhibitors (SSRIs)** are also not well studied for symptoms other than depression but may be appropriate for nonpsychotic patients with agitation, especially for patients with mild agitation or prior sensitivity to antipsychotic medications.  [III] |
|  | **Treatment of Depression** |
| **Adopt** | **Depression is common** in patients with dementia. Patients with depression should be evaluated for suicide risk.  [I] |
| **Adopt** | Depressed mood may respond to improvements in the patient’s living situation or to **stimulation-oriented treatments.**  [II] |
| **Adopt** | Although evidence for antidepressant efficacy in patients with dementia and depression is mixed, clinical consensus supports a **trial of an antidepressant** to treat clinically significant, persistent depressed mood.  [II] |
| **Adopt** | The choice among agents is based on the side-effect profile of specific medications and the characteristics of the individual patient.  [I] |
| **Adopt** | **SSRIs may be preferred** because they appear to be **better tolerated** than other antidepressants. [II] |
| **Adopt** | **Bupropion, venlafaxine, and mirtazapine** may also be effective.  [II] |
| **Adopt** | Agents with substantial anticholinergic effects (e.g., amitriptyline, imipramine) should be avoided. [I] |
| **Refer** | Despite the lack of research data, clinical experience suggests that **unilateral electroconvulsive therapy (ECT)** may be **effective** for patients who do not respond to pharmacological agents.  [II] |
| **Adopt** | Treatments for **apathy** are not well supported, but **psychostimulants, bupropion, bromocriptine, and amantadine** may be helpful [III]. Psychostimulants are also sometimes useful in the treatment of depression in patients with significant general medical illness.  [III] |
|  | **Treatment of Sleep Disturbances** |
| **Adopt** | Sleep disturbances are common in patients with dementia. Interventions include maintaining **daytime activities** and giving **careful attention to sleep hygiene.**  [II] |
| **Adopt** | **Pharmacological intervention** could be considered when other approaches have failed.  [II] |
| **Adopt** | If a patient also requires medication for another psychiatric condition, an agent with **sedating properties**, given at bedtime, could be selected.  [I] |
| **Adopt** | For primarily treating the sleep disturbance, medications with possible effectiveness include **trazodone, zolpidem, or zaleplon,**  [III] |
| **Adopt** | but there are few data on the efficacy of specific agents. **Benzodiazepines are not recommended** for other than brief use because of risks of daytime sedation, tolerance, rebound insomnia, worsening cognition, falls, disinhibition, and delirium.  [II] |
| **Adopt** | **Diphenhydramine** is **not recommended** because of its anticholinergic properties.  [II] |
| **Adopt** | **Antipsychotic medications should not be used** solely for the purpose of treating sleep disturbances.  [I] |
|  | **Special Issues for Long-Term Care** |
| **Adopt** | Many patients eventually require **long-term-care placement**; approximately two-thirds of nursing home patients have dementia. Care should be organized to meet the needs of patients, including those with behavioural problems.  [I] |
| **Adopt** | Employing **staff with knowledge and experience** concerning dementia and the management of difficult behaviour is important.  [II] |
| **Adopt** | Special care units may offer more optimal care, although there is limited evidence that they achieve better outcomes than traditional units.  [III] |
| **Adopt** | A particular concern is the use of **physical restraints and medications to control disruptive behaviour**. Appropriate use of antipsychotic medications can relieve symptoms and reduce distress and can increase safety for patients, other residents, and staff.  [I] |
| **Adopt** | However, their use may be associated with worsening cognitive impairment, oversedation, falls, tardive dyskinesia, and neuroleptic malignant syndrome, as well as with hyperlipidemia, weight gain, diabetes mellitus, cerebrovascular accidents, and death.  [I] |
| **Adopt** | Thus, good clinical practice requires **careful consideration and documentation of the indications and available alternatives**, both initially and on a regular ongoing basis.  [I] |
| **Adopt** | A **dose decrease or discontinuation** should be considered periodically for all patients who receive antipsychotic medications.  [I] |
| **Adopt** | A **structured education program** for staff may help to both manage patients’ behaviour and decrease the use of these medications in nursing homes.  [II] |
| **Adopt** | Physical restraints are rarely indicated and should be used only for patients who pose an imminent risk of physical harm to themselves or others.  [I] |
| **Adopt** | Reasons for the use of physical restraints should be carefully documented.  [I] |
| **Adopt** | The need for restraints can be decreased by **environmental changes** that decrease the risk of falls or wandering and by careful assessment and treatment of possible causes of agitation.  [II] |

**ACRONYMS AND ABBREVIATIONS**

| ADLs | Activities of Daily Livings | LFT | Liver Function Test |
| --- | --- | --- | --- |
| CT | Computed Tomography | MoCA | Montreal Cognitive Assessment |
| CBC | Complete blood count | MRI | Magnetic Resonance Imaging |
| DLB | Dementia with Lewy Bodies | NSAIDs | Non-Steroidal Anti-Inflammatory Drugs |
| EEG | Electroencephalogram | NMDA | N-Methyl D-Aspartate |
| ECT | Electroconvulsive Therapy | SPECT | Single Photon Emission Computed Tomography |
| FDA | Food & Drug Administration | SSRIs | Selective Serotonin Reuptake Inhibitors |
| FDG-PET | Fluorodeoxyglucose -Positron Emission Tomography | TSH | Thyroid- Stimulating Hormone |
| HIV | Human Deficiency Virus |  |  |

​​​​

**SECTION 9: EATING DISORDERS MANAGEMENT GUIDELINES**

**Eating Disorders Management Guidelines**

**Source guideline:** Practice Guidelines for the Treatment of Patients with Eating Disorders, 2006, American Psychiatric Association.^11^

**Key to understanding strength of recommendation.**

| Strength of Recommendation | |
| --- | --- |
| [I] | Recommended with substantial clinical confidence |
| [II] | Recommended with moderate clinical confidence |
| [III] | May be recommended on the basis of individual circumstances |

**Table of Recommendation**

| **Psychiatric Management** | |
| --- | --- |
| **Adopt** | Psychiatric management begins with the establishment of a **therapeutic alliance**, which is enhanced by empathic comments and behaviors, positive regard, reassurance, and support.  [I] |
| **Adopt** | Basic psychiatric management includes **support** through the provision of educational materials, including self-help workbooks; information on community-based and Internet resources; and direct advice to patients and their families (if they are involved).  [I] |
| **Adopt** | A **team approach** is the recommended model of care.  [I] |
| **Coordinating care and collaborating with other clinicians** | |
| **Adopt** | In treating adults with eating disorders, the psychiatrist may assume the **leadership role** within a program or team that includes other physicians, psychologists, registered dietitians, and social workers or may work collaboratively on a team led by others. For the management of acute and ongoing medical and dental complications, it is important that psychiatrists **consult** other physician specialists and dentists.  [I] |
| **Adopt** | When a patient is managed by an interdisciplinary team in an outpatient setting, **communication** among the professionals is essential to monitoring the patient’s progress, making necessary adjustments to the treatment plan, and delineating the specific roles and tasks of each team member.  [I] |
| **Assessing and monitoring eating disorder symptoms and behaviors** | |
| **Adopt** | A **careful assessmen**t of the patient’s history, symptoms, behaviors, and mental status is the first step in making a diagnosis of an eating disorder.  [I] |
| **Adopt** | The complete assessment usually requires at least **several hours** and includes a thorough review of the patient’s height and weight history; restrictive and binge eating and exercise patterns and their changes; purging and other compensatory behaviors; core attitudes regarding weight, shape, and eating, and associated psychiatric conditions.  [I] |
| **Adopt** | A **family history** of eating disorders or other psychiatric disorders, including alcohol and other substance use disorders; a family history of obesity; family interactions in relation to the patient’s disorder; and family attitudes toward eating, exercise, and appearance are all relevant to the assessment.  [I] |
| **Adopt** | A **clinician’s articulation of theories** that imply blame or permit family members to blame one another or themselves can alienate family members from involvement in the treatment and therefore be detrimental to the patient’s care and recovery.  [I] |
| **Adopt** | It is important to **identify family stressors** whose amelioration may facilitate recovery.  [I] |
| **Assessing and monitoring the patient’s general medical condition** | |
| **Adopt** | A **full physical examination** of the patient is strongly recommended and may be performed by a physician familiar with common findings in patients with eating disorders. The examination should give particular attention to vital signs, physical status (including height and weight), cardiovascular and peripheral vascular function, dermatological manifestations, and evidence of self-injurious behaviors.  [I] |
| **Adopt** | Calculation of the patient’s **body mass index** (BMI) is also useful.  [I] |
| **Adopt** | **Early recognition** of eating disorder symptoms and early intervention may prevent an eating disorder from becoming chronic.  [I] |
| **Adopt** | During treatment, it is important to **monitor the patient** for shifts in weight, blood pressure, pulse, other cardiovascular parameters, and behaviors likely to provoke physiological decline and collapse.  [I] |
| **Adopt** | Patients with a history of **purging behaviors** should also be referred for a **dental examination**.  [I] |
| **Adopt** | **Bone density examinations** should be obtained for patients who have been amenorrheic for 6 months or more.  [I] |
| **Adopt** | In **younger patients, examination** should include growth pattern, sexual development (including sexual maturity rating), and general physical development.  [I] |
| **Adopt** | The need for **laboratory analyses** should be determined on an **individual basis** depending on the patient’s condition or the laboratory tests’ relevance to making treatment decisions.  [I] |
| **Assessing and monitoring the patient’s safety and psychiatric status** | |
| **Adopt** | The **patient’s safety** will be enhanced when **particular attention** is given to suicidal ideation, plans, intentions, and attempts as well as to impulsive and compulsive self-harm behaviors.  [I] |
| **Adopt** | **Other aspects** of the patient’s psychiatric status that greatly influence clinical course and outcome and that are important to assess include mood, anxiety, and substance use disorders, as well as motivational status, personality traits, and personality disorders.  [I] |
| **Adopt** | **Assessment for suicidality** is of particular importance in patients with co-occurring alcohol and other substance use disorders.  [I] |
| **Providing family assessment and treatment** | |
| **Adopt** | For **older patients**, family assessment and involvement may be useful and should be considered on a case-by-case basis.  [II] |
| **Adopt** | **Involving spouses and partners** in treatment may be highly desirable [II]. |
| **Choosing a treatment site** | |
| **Adopt/**  **Refer** | Services available for treating eating disorders can range from **intensive inpatient programs** (in which general medical care is readily available) to **residential and partial hospitalization programs** to varying levels of **outpatient care** (in which the patient receives general medical treatment, nutritional counseling, and/or individual, group, and family psychotherapy). Because specialized programs are not available in all geographic areas and their financial requirements are often significant, access to these programs may be limited; petition, explanation, and follow-up by the psychiatrist on behalf of patients and families may help procure access to these programs. **Pretreatment evaluation** of the patient is essential in choosing the appropriate treatment setting.  [I] |
| **Adopt** | In determining a patient’s initial level of care or whether a change to a different level of care is appropriate, it is important to consider the **patient’s overall physical condition, psychology, behaviors, and social circumstances** rather than simply rely on one or more physical parameters, such as weight.  [I]. |
| **Adopt** | **Weight in relation** to estimated individually healthy weight, the rate of weight loss, cardiac function, and metabolic status are the most important physical parameters to be considered when choosing a treatment setting; other **psychosocial parameters** are also important  [I]. |
| **Adopt** | **Healthy weight estimates** for a given individual must be determined by that person’s physicians  [I]. |
| **Adopt** | Such estimates may be based on **historical considerations** (often including that person’s growth charts) and, for women, the weight at which healthy menstruation and ovulation resume, which may be higher than the weight at which menstruation and ovulation become impaired. Admission to or continuation of an intensive level of care (e.g., hospitalization) may be necessary when access to a less intensive level of care (e.g., partial hospitalization) is absent because of geography or a lack of resources  [I]. |
| **Adopt** | Generally, adult patients who **weigh less than approximately 85%** of their individually estimated healthy weights have considerable difficulty gaining weight outside of a highly structured program  [II]. |
| **Refer** | Such programs, including **inpatient care**, may be medically and psychiatrically necessary even for some patients who are above 85% of their individually estimated healthy weight.  [I] |
| **Refer** | **Factors suggesting that hospitalization may be appropriate** include rapid or persistent decline in oral intake, a decline in weight despite maximally intensive outpatient or partial hospitalization interventions, the presence of additional stressors that may interfere with the patient’s ability to eat, knowledge of the weight at which instability previously occurred in the patient, co-occurring psychiatric problems that merit hospitalization, and the degree of the patient’s denial and resistance to participate in his or her own care in less intensively supervised settings.  [I] |
| **Refer** | Hospitalization should occur **before the onset of medical instability** as manifested by abnormalities in vital signs (e.g., marked orthostatic hypotension with an increase in pulse of 20 bpm or a drop in standing blood pressure of 20 mmHg, bradycardia <40 bpm, tachycardia >110 bpm, or an inability to sustain core body temperature), physical findings, or laboratory tests.  [I] |
| **Refer** | Patients who are **physiologically stabilized** on acute medical units will still require specific inpatient treatment for eating disorders if they do not meet biopsychosocial criteria for less in- tensive levels of care and/or if no suitable less intensive levels of care are accessible because of geographic or other reasons.  [I] |
| **Adopt** | **Weight level** per se should **never be used as the sole criterion** for discharge from inpatient care  [I] |
| **Adopt** | Assisting patients in determining and practicing **appropriate intake** at a healthy body weight is likely to decrease the chances of their relapsing after discharge.  [I] |
| **Adopt** | In shifting between levels of care, it is important to establish **continuity of care.**  [II] |
| **Adopt** | If the patient is going from one treatment setting or locale to another, **transition planning** requires that the care team in the new setting or locale be identified and that specific patient appointments be made.  [I] |
| **Adopt** | It is preferable that a **specific clinician** on the team be designated as the **primary coordinator** of care to ensure continuity and attention to important aspects of treatment.  [II] |
| **Adopt** | Most patients with **uncomplicated bulimia nervosa** do not require hospitalization; indications for the hospitalization of such patients include severe disabling symptoms that have not responded to adequate trials of outpatient treatment, serious concurrent general medical problems (e.g., metabolic abnormalities, hematemesis, vital sign changes, uncontrolled vomiting), suicidality, psychiatric disturbances that would warrant the patient’s hospitalization independent of the eating disorder diagnosis, or severe concurrent alcohol or drug dependence or abuse.  [I] |
| **Refer** | **Legal interventions**, including involuntary hospitalization and legal guardianship, may be necessary to address the safety of treatment-reluctant patients whose general medical conditions are life-threatening. [I] |
| **Refer** | There is evidence to suggest that patients with eating disorders have **better outcomes** when treated in **inpatient units** specializing in the treatment of these disorders than when treated in general inpatient settings where staff lack expertise and experience in treating eating disorders.  [II] |
| **Refer** | Outcomes from **partial hospitalization programs** that specialize in eating disorders are highly correlated with treatment intensity. The more successful programs involve patients in treatment at least **5 days/week for 8 hours/day**; thus, it is recommended that partial hospitalization programs be structured to provide at least this level of care.  [I] |
| **Adopt** | Patients who are considerably below their healthy body weight and are highly motivated to adhere to treatment, have cooperative families, and have a brief symptom duration may benefit from treatment in **outpatient settings**, but only if they are carefully monitored and if they and their families understand that a more restrictive setting may be necessary if persistent progress is not evident in a few weeks.  [II] |
| **Adopt** | **Careful monitoring** includes at least weekly (and often two to three times a week) weight determinations done directly after the patient voids and while the patient is wearing the same class of garment (e.g., hospital gown, standard exercise clothing).  [I] |
| **Adopt** | In patients who purge, it is important to routinely **monitor serum electrolytes**.  [I] |
| **Adopt** | **Urine-specific gravity, orthostatic vital signs, and oral temperatures** may need to be measured on a regular basis.  [II] |
| **Adopt** | In an outpatient setting, patients can remain with their families and continue to attend school or work. Inpatient care may interfere with family, school, and work obligations; however, it is important to give priority to the safe and adequate treatment of a rapidly progressing or otherwise unresponsive disorder for which hospital care might be necessary.  [I] |
| **Choice of specific treatments for anorexia nervosa**  **a) Nutritional rehabilitation** | |
| **Adopt** | The **goals of nutritional rehabilitation** for seriously underweight patients are to restore weight, normalize eating patterns, achieve normal perceptions of hunger and satiety, and correct biological and psychological sequelae of malnutrition.  [I] |
| **Adopt** | For **patients age 20 years and younger**, an individually appropriate range for expected weight and goals for weight and height may be determined by considering measurements and clinical factors, including current weight, bone age estimated from wrist X-rays and nomograms, menstrual history, mid-parental heights, assessments of skeletal frame, and benchmarks from Centers for Disease Control and Prevention (CDC) growth charts.  [I] |
| **Refer** | For individuals who are **markedly underweight** and whose weight has deviated below their growth curves, **hospital-based programs** for nutritional rehabilitation should be considered.  [I] |
| **Adopt** | For patients in inpatient or residential settings, the discrepancy between healthy target weight and weight at discharge may vary depending on patients’ ability to feed themselves, their motivation and ability to participate in aftercare programs, and the adequacy of aftercare, including partial hospitalization.  [I] |
| **Adopt** | It is important to implement re-feeding programs in **nurturing emotional contexts**.  [I] |
| **Adopt** | For example, it is useful for staff to convey to patients their **intention to take care** of them and not let them die even when the illness prevents the patients from taking care of themselves.  [II] |
| **Adopt** | It is also useful for staff to communicate clearly that they are not seeking to engage in **control battles** and have **no punitive intentions** when using interventions that the patient may experience as aversive.  [I] |
| **Adopt** | In working to achieve target weights, the treatment plan should also establish expected rates of controlled weight gain. Clinical consensus suggests that realistic targets are **2–3 lb/week for hospitalized patients** and **0.5–1 lb/week for individuals in outpatient programs**.  [II] |
| **Adopt** | Registered dietitians can help patients choose their own meals and can provide a **structured meal plan** that ensures nutritional adequacy and that none of the major food groups are avoided.  [I] |
| **Adopt** | **Formula feeding** may have to be added to the patient’s diet to achieve large caloric intake.  [II] |
| **Adopt** | It is important to encourage patients with anorexia nervosa to **expand their food choices** to minimize the severely restricted range of foods initially acceptable to them.  [II] |
| **Adopt** | Caloric intake levels should usually start at **30–40 kcal/kg per day** (approximately 1,000–1,600 kcal/day). During the weight gain phase, intake may have to be advanced progressively to as high as **70–100 kcal/kg per day** for some patients; many male patients require a very large number of calories to gain weight.  [II] |
| **Adopt** | Patients who require much lower caloric intakes or are suspected of artificially increasing their weight by fluid loading should be **weighed in the morning** after they have voided and are wearing only a gown; their fluid intake should also be carefully monitored.  [I] |
| **Adopt** | Urine specimens obtained at the time of a patient’s weigh-in may need to be assessed for specific gravity to help ascertain the extent to which the measured weight reflects excessive water intake.  [I] |
| **Adopt** | Regular monitoring of **serum potassium levels** is recommended in patients who are persistent vomiters.  [I] |
| **Adopt** | Hypokalemia should be treated with **oral or intravenous potassium** supplementation and rehydration.  [I] |
| **Adopt** | **Physical activity** should be adapted to the food intake and energy expenditure of the patient, taking into account the patient’s bone mineral density and cardiac function.  [I] |
| **Adopt** | Once a safe weight is achieved, the focus of an **exercise program** should be on the patient’s gaining physical fitness as opposed to expending calories.  [I] |
| **Adopt** | Weight gain results in **improvements in most of the physiological and psychological complications** of semistarvation.  [I] |
| **Adopt** | It is important to warn patients about the following aspects of early recovery.  [I] |
| **Adopt** | As they start to recover and feel their bodies getting larger, especially as they approach frightening, magical numbers on the scale that represent phobic weights, they may experience a **resurgence of anxious and depressive symptoms, irritability, and sometimes suicidal thoughts**. These mood symptoms, non-food-related obsessional thoughts, and compulsive behaviors, although often not eradicated, usually decrease with sustained weight gain and weight maintenance. Initial refeeding may be associated with mild transient fluid retention, but patients who abruptly stop taking laxatives or diuretics may experience marked rebound fluid retention for several weeks. As weight gain progresses, many patients also develop acne and breast tenderness and become unhappy and demoralized about the resulting changes in body shape. Patients may experience abdominal pain and bloating with meals from the delayed gastric emptying that accompanies malnutrition. These symptoms may respond to **promotility agents**.  [III] |
| **Adopt** | **Constipation** may be ameliorated with stool softeners; if unaddressed, it can progress to obstipation and, rarely, to acute bowel obstruction. When life-preserving nutrition must be provided to a patient who refuses to eat, nasogastric feeding is preferable to intravenous feeding.  [I] |
| **Adopt** | When nasogastric feeding is necessary, **continuous feeding** (i.e., over 24 hours) may be better tolerated by patients and less likely to result in metabolic abnormalities than three to four bolus feedings a day.  [II] |
| **Refer** | In very difficult situations, where patients physically resist and constantly remove their nasogastric tubes, feeding through **surgically placed gastrostomy or jejunostomy tubes** may be an alternative to nasogastric feeding.  [II] |
| **Adopt** | In determining **whether to begin involuntary forced feeding**, the clinician should carefully think through the clinical circumstances, family opinion, and relevant legal and ethical dimensions of the patient’s treatment.  [I] |
| **Adopt** | The **general principles** to be followed in making the decision are those directing good, humane care; respecting the wishes of competent patients; and intervening respectfully with patients whose judgment is severely impaired by their psychiatric disorders when such interventions are likely to have beneficial results.  [I] |
| **Adopt** | For cooperative patients, **supplemental overnight pediatric nasogastric tube feeding** has been used in some programs to facilitate weight gain.  [III] |
| **Adopt** | With **severely malnourished patients** (particularly those whose weight is <70% of their healthy body weight) who undergo aggressive oral, nasogastric, or parenteral refeeding, a serious refeeding syndrome can occur. Initial assessments should include vital signs and food and fluid intake and output, if indicated, as well as monitoring for edema, rapid weight gain (associated primarily with fluid overload), congestive heart failure, and gastrointestinal symptoms.  [I] |
| **Adopt** | Patients’ serum levels of **phosphorus, magnesium, potassium, and calcium** should be determined daily for the first 5 days of refeeding and every other day for several weeks thereafter, and **electrocardiograms** should be performed as indicated.  [II] |
| **Adopt** | Phosphorus, magnesium, and/or potassium supplementation should be given when indicated.  [I] |
| **Psychosocial interventions**  **(i) Acute anorexia nervosa** | |
| **Adopt/**  **Refer** | During acute refeeding and while weight gain is occurring, it is beneficial to provide anorexia nervosa patients with **individual psychotherapeutic management** that is psychodynamically informed and provides empathic understanding, explanations, praise for positive efforts, coaching, support, encouragement, and other positive behavioral reinforcement.  [I] |
| **Adopt** | Attempts to conduct formal **psychotherapy with starving patients** who are often negativistic, obsessional, or mildly cognitively impaired may be **ineffective**.  [II] |
| **Adopt** | In methods modeled after the **Maudsley approach**, families become actively involved, in a blame-free atmosphere, in helping patients eat more and resist compulsive exercising and purging. For some outpatients, a short-term course of family therapy using these methods may be as effective as a long-term course; however, a shorter course of therapy may not be adequate for patients with severe obsessive-compulsive features or nonintact families.  [II] |
| **Refer** | Most **inpatient-based nutritional rehabilitation programs** create a milieu that incorporates emotional nurturance and a combination of reinforcers that link exercise, bed rest, and privileges to target weights, desired behaviors, feedback concerning changes in weight, and other observable parameters.  [II] |
| **(ii) Anorexia nervosa after weight restoration** | |
| **Adopt/**  **Refer** | Once malnutrition has been corrected and weight gain has begun, **psychotherapy** can help patients with anorexia nervosa understand  1) their experience of their illness;  2) cognitive distortions and how these have led to their symptomatic behavior;  3) developmental, familial, and cultural antecedents of their illness;  4) how their illness may have been a maladaptive attempt to regulate their emotions and cope;  5) how to avoid or minimize the risk of relapse; and  6) how to better cope with salient developmental and other important life issues in the future. Clinical experience shows that patients may often display improved mood, enhanced cognitive functioning, and clearer thought processes after there is significant improvement in nutritional in- take, even before there is substantial weight gain.  [II] |
| **Adopt/**  **Refer** | To help prevent patients from relapsing, emerging data support the use of **cognitive-behavioral psychotherapy** for adults.  [II] |
| **Adopt/**  **Refer** | Many clinicians also use **interpersonal and/or psychodynamically oriented** individual or group psychotherapy for adults after their weight has been restored.  [II] |
| **Adopt/**  **Refer** | Although studies of different psychotherapies focus on these interventions as distinctly separate treatments, in practice there is a **frequent overlap of interventions**.  [II] |
| **Adopt/**  **Refer** | It is important for clinicians to pay attention to **cultural attitudes**, patient issues involving the **gender of the therapist**, and specific concerns about **possible abuse, neglect, or other developmental traumas**  [II] |
| **Adopt/**  **Refer** | Clinicians need to attend to their **countertransference reactions** to patients with a chronic eating disorder, which often include beleaguerment, demoralization, and excessive need to change the patient.  [I] |
| **Adopt/**  **Refer** | At the same time, when treating patients with chronic illnesses, clinicians need to understand the **longitudinal course of the disorder** and that patients can recover even after many years of illness.  [I] |
| **Adopt/**  **Refer** | Because of anorexia nervosa’s enduring nature, **psychotherapeutic treatment** is frequently required for **at least 1 year** and may take many years.  [I] |
| **Adopt** | Anorexics and Bulimics Anonymous and Overeaters Anonymous are **not** substitutes for professional treatment  [I] |
| **Adopt** | Programs that focus exclusively on abstaining from binge eating, purging, restrictive eating, or excessive exercising (e.g., 12-step programs) without attending to nutritional considerations or cognitive and behavioral deficits have not been studied and therefore cannot be recommended as the sole treatment for anorexia nervosa.  [I] |
| **Adopt/**  **Refer** | It is important for programs using 12-step models to be equipped to care for patients with the substantial **psychiatric and general medical problems** often associated with eating disorders.  [I] |
| **Adopt** | Although families and patients are increasingly accessing worthwhile, helpful information through online websites, newsgroups, and chat rooms, the lack of professional supervision within these resources may sometimes lead to users’ receiving misinformation or create unhealthy dynamics among users. It is recommended that **clinicians inquire about a patient’s or family’s use of Internet-based support** and other alternative and complementary approaches and be prepared to openly and sympathetically discuss the information and ideas gathered from these sources.  [I] |
| **(iii) Chronic anorexia nervosa** | |
| **Adopt/**  **Refer** | Patients with chronic anorexia nervosa generally show a **lack of substantial clinical response** to formal **psychotherapy**. Nevertheless, many clinicians report seeing patients with chronic anorexia nervosa who, after many years of struggling with their disorder, experience substantial remission, so clinicians are justified in maintaining and extending some degree of **hope** to patients and families.  [II] |
| **Adopt/**  **Refer** | More **extensive psychotherapeutic measures** may be undertaken to engage and help motivate patients whose illness is resistant to treatment.  [II] |
| **Adopt/**  **Refer** | or, failing that, as **compassionate care**.  [I] |
| **Adopt/**  **Refer** | For patients who have difficulty talking about their problems, clinicians have reported that a variety of **nonverbal therapeutic methods**, such as the creative arts, movement therapy programs, and occupational therapy, can be useful.  [III] |
| **Adopt/**  **Refer** | **Psychosocial programs** designed for patients with chronic eating disorders are being implemented at several treatment sites and may prove useful.  [II] |
| **Medications and other somatic treatments**  **(i) Weight restoration** | |
| **Adopt** | The decision about whether to use psychotropic medications and, if so, which medications to choose will be based on the patient’s **clinical presentation**.  [I] |
| **Adopt** | The limited empirical data on malnourished patients indicate that **selective serotonin reuptake inhibitors** (SSRIs) **do not appear to confer an advantage** regarding weight gain in patients who are concurrently receiving inpatient treatment in an organized eating disorder program.  [I] |
| **Adopt/**  **Refer** | However, **SSRIs in combination with psychotherapy** are widely used in treating patients with anorexia nervosa. For example, these medications may be considered for those with persistent depressive, anxiety, or obsessive-compulsive symptoms and for bulimic symptoms in weight-restored patients.  [II] |
| **Adopt** | A U.S. Food and Drug Administration (FDA) black box warning concerning the use of bupropion in patients with eating disorders has been issued because of the **increased seizure risk** in these patients. Adverse reactions to **tricyclic antidepressants and monoamine oxidase inhibitors** (MAOIs) are more pronounced in malnourished individuals, and these medications should generally be **avoided** in this patient population.  [I] |
| **Adopt** | **Second-generation antipsychotics**, particularly olanzapine, risperidone, and quetiapine, have been used in small series and individual cases for patients, but controlled studies of these medications are lacking. Clinical impressions suggest that they may be useful in patients with severe, unremitting resistance to gaining weight; severe obsessional thinking; and denial that assumes delusional proportions.  [III] |
| **Adopt** | Small doses of **older antipsychotics** such as chlorpromazine may be helpful prior to meals in very disturbed patients.  [III] |
| **Adopt** | Although the risks of **extrapyramidal side effects** are less with second-generation antipsychotics than with first-generation antipsychotics, debilitated anorexia nervosa patients may be at a higher risk for these than expected. Therefore, if these medications are used, it is recommended that patients be carefully monitored for extrapyramidal symptoms and **akathisia**.  [I] |
| **Adopt** | It is also important to **routinely monitor patients** for potential side effects of these medications, which can result in insulin resistance, abnormal lipid metabolism, and prolongation of the QTc interval.  [I] |
| **Adopt** | Because **ziprasidone** has not been studied in individuals with anorexia nervosa and can prolong QTc intervals, careful monitoring of **serial electrocardiograms and serum potassium** measurements is needed if anorexic patients are treated with ziprasidone.  [I] |
| **Adopt** | **Antianxiety agents** used selectively before meals may be useful to reduce patients’ anticipatory anxiety before eating.  [III] |
| **Adopt** | but because eating disorder patients may have a **high propensity to become dependent** on **benzodiazepines**, these medications should be used routinely only with considerable caution.  [I] |
| **Adopt** | **Pro-motility agents** such as **metoclopramide** may be useful for bloating and abdominal pains that occur during refeeding in some patients.  [II] |
| **Refer** | **Electroconvulsive therapy (ECT)** has generally **not been useful** except in treating severe co-occurring disorders for which ECT is otherwise indicated.  [I] |
| **Adopt** | Although no specific hormone treatments or vitamin supplements have been shown to be helpful.  [I] |
| **Adopt** | supplemental **calcium and vitamin D** are often recommended.  [III] |
| **Adopt** | **Zinc supplements** have been reported to foster weight gain in some patients, and patients may benefit from daily zinc-containing multivitamin tablets.  [II] |
| **(ii) Relapse prevention** | |
| **Adopt** | Some data suggest that **fluoxetine** in doses of up to **60 mg/day** may help prevent relapse.  [II] |
| **Adopt**/  **Refer** | For patients receiving cognitive-behavioral therapy (CBT) after weight restoration, adding fluoxetine does not appear to confer additional benefits with respect to preventing relapse.  [II] |
| **Adopt** | **Antidepressants and other psychiatric medications** may be used to treat specific, ongoing psychiatric symptoms of depressive, anxiety, obsessive-compulsive, and other comorbid disorders  [I] |
| **Adopt** | Clinicians should attend to the **black box warnings** in the package inserts relating to anti-depressants and discuss the potential benefits and risks of antidepressant treatment with patients and families if such medications are to be prescribed.  [I] |
| **(iii) Chronic anorexia nervosa** | |
| **Adopt** | Although **hormone replacement therapy (HRT)** is frequently prescribed to improve bone mineral density in female patients, **no good supporting evidence** exists either in adults to demonstrate its efficacy.  [II] |
| **Adopt** | Hormone therapy usually induces **monthly menstrual bleeding**, which may contribute to the patient’s denial of the need to gain further weight.  [II] |
| **Adopt** | Before estrogen is offered, it is recommended that efforts be made to increase weight and achieve resumption of **normal menses**.  [I] |
| **Adopt** | There is **no indication for the use of bisphosphonates** such as alendronate in patients with anorexia nervosa  [II] |
| **Adopt** | Although there is no evidence that calcium or vitamin D supplementation reverses decreased bone mineral density, when calcium dietary intake is inadequate for growth and maintenance, **calcium supplementation** should be considered.  [I] |
| **Adopt** | and when the individual is not exposed to daily sunlight, **vitamin D supplementation** may be used.  [I] |
| **Adopt** | However, large supplemental doses of vitamin D may be **hazardous**.  [I] |
| **Choice of specific treatments for bulimia nervosa**  **a) Nutritional rehabilitation counseling** | |
| **Adopt** | A primary focus for nutritional rehabilitation is to help the patient develop a **structured meal plan** as a means of reducing the episodes of dietary restriction and the urges to binge and purge.  [I] |
| **Adopt** | Adequate nutritional intake can **prevent craving and promote satiety**.  [I] |
| **Adopt** | It is important to **assess nutritional intake** for all patients, even those with a normal body weight (or normal BMI), as normal weight does not ensure appropriate nutritional intake or normal body composition.  [I] |
| **Adopt** | Among patients of normal weight, **nutritional counseling** is a useful part of treatment and helps reduce food restriction, increase the variety of foods eaten, and promote healthy but not compulsive exercise patterns  [I] |
| **b) Psychosocial interventions** | |
| **Adopt** | It is recommended that psychosocial interventions be chosen on the basis of a **comprehensive evaluation** of the individual patient that takes into consideration the patient’s cognitive and psychological development, psychodynamic issues, cognitive style, comorbid psychopathology, and preferences as well as patient age and family situation.  [I] |
| **Adopt**/  **Refer** | For treating **acute episodes of bulimia nervosa** in adults, the evidence strongly supports the value of **CBT** as the most effective single intervention.  [I] |
| **Adopt**/  **Refer** | Some patients who do not respond initially to CBT may respond when switched to either **interpersonal therapy (IPT) or fluoxetine**.  [II] |
| **Adopt**/  **Refer** | or other modes of treatment such as **family and group psychotherapies**  [III] |
| **Adopt**/  **Refer** | Controlled trials have also shown the utility of IPT in some cases.  [II] |
| **Adopt**/  **Refer** | In clinical practice, many practitioners combine elements of CBT, IPT, and other psychotherapeutic techniques. Compared with psychodynamic or interpersonal therapy, CBT is associated with more rapid remission of eating symptoms.  [I] |
| **Adopt**/  **Refer** | but using psychodynamic interventions in conjunction with CBT and other psychotherapies may yield better global outcomes.  [II] |
| **Adopt**/  **Refer** | Some patients, particularly those with **concurrent personality pathology** or other co-occurring disorders, require lengthy treatment.  [II] |
| **Adopt**/  **Refer** | Clinical reports suggest that psychodynamic and psychoanalytic approaches in individual or group format are useful once bingeing and purging improve.  [III] |
| **Adopt**/  **Refer** | older patients with ongoing conflicted interactions with **parents** [III]. |
| **Adopt**/  **Refer** | Patients with marital discord may benefit from **couples therapy** [II]. |
| **Adopt**/  **Refer** | A variety of **self-help** and **professionally guided self-help programs** have been effective for some patients with bulimia nervosa.  [I] |
| **Adopt**/  **Refer** | Several innovative **online programs** are currently under investigation and may be recommended in the absence of alternative treatments.  [III] |
| **Adopt**/  **Refer** | **Support groups and 12-step programs** such as Overeaters Anonymous may be helpful as adjuncts in the initial treatment of bulimia nervosa and for subsequent relapse prevention, but they are not recommended as the sole initial treatment approach for bulimia nervosa.  [I] |
| **Adopt**/  **Refer** | Issues of **countertransference** discussed above with respect to the treatment of patients with anorexia nervosa, also apply to the treatment of patients with bulimia nervosa.  [I] |
| **c) Medications**  **(i) Initial treatment** | |
| **Adopt** | **Antidepressants** are effective as one component of an initial treatment program for most bulimia nervosa patients.  [I] |
| **Adopt** | with **SSRI treatment** having the most evidence for efficacy and the fewest difficulties with adverse effects.  [I] |
| **Adopt** | To date, **fluoxetine** is the best studied of these and is the only FDA-approved medication for bulimia nervosa. **Sertraline** is the only other SSRI that has been shown to be effective, as demonstrated in a small, randomized controlled trial. In the absence of therapists qualified to treat bulimia nervosa with CBT, fluoxetine is recommended as an initial treatment.  [I] |
| **Adopt** | Dosages of SSRIs higher than those used for depression (e.g., fluoxetine 60 mg/day) are more effective in treating bulimic symptoms.  [I] |
| **Adopt** | Antidepressants may be helpful for patients with substantial concurrent symptoms **of depression, anxiety, obsessions, or certain impulse disorder symptoms** or for patients who have not benefited from or had only a suboptimal response to appropriate psychosocial therapy.  [I] |
| **Adopt** | **Tricyclic antidepressants and MAOIs** have been rarely used with bulimic patients and **are not recommended** as initial treatments.  [I] |
| **Adopt** | Several different antidepressants may have to be tried sequentially to **identify the specific medication with the optimum effect**.  [I] |
| **Adopt** | Clinicians should attend to the **black box warnings** relating to antidepressants and discuss the potential benefits and risks of antidepressant treatment with patients and families if such medications are to be prescribed.  [I] |
| **Adopt** | Small, controlled trials have demonstrated the efficacy of the anticonvulsant medication **topiramate**, but because adverse reactions to this medication are common, it should be used only when other medications have proven ineffective.  [III] |
| **Adopt** | Also, because patients tend to **lose weight** on topiramate, its use is problematic for normal or underweight individuals.  [III] |
| **Adopt** | Two drugs that are used for mood stabilization, **lithium and valproic acid**, are both prone to induce weight gain in patients.  [I] |
| **Adopt** | and may be less acceptable to patients who are weight pre-occupied. However, lithium is not recommended for patients with bulimia nervosa because it is ineffective.  [I] |
| **Adopt** | In patients with **co-occurring bulimia nervosa and bipolar disorder**, treatment with lithium is more likely to be associated with **toxicity**.  [I] |
| **(ii) Maintenance phase** | |
| **Adopt** | Limited evidence supports the use of **fluoxetine** for **relapse prevention**.  [II] |
| **Adopt** | but substantial rates of relapse occur even with treatment. In the absence of adequate data, most clinicians recommend continuing antidepressant therapy for a **minimum of 9 months** and probably for a year in most patients with bulimia nervosa.  [II] |
| **Adopt** | Case reports indicate that **methylphenidate** may be helpful for bulimia nervosa patients with concurrent **attention-deficit/hyperactivity disorder (ADHD).**  [III] |
| **Adopt** | but it should be used only for patients who have a very clear diagnosis of ADHD.  [I] |
| **(iii) Combining psychosocial interventions and medications** | |
| **Adopt/Refer** | In some research, the combination of **antidepressant therapy and CBT** results in the **highest remission rates**; therefore, this combination is recommended initially when qualified CBT therapists are available. [II] |
| **Adopt/Refer** | In addition, when CBT alone does not result in a substantial reduction in symptoms after 10 sessions, it is recommended that fluoxetine be added.  [II] |
| **(iv) Other treatments** | |
| **Adopt** | Bright light therapy has been shown to reduce binge frequency in several controlled trials and may be used as an adjunct when CBT and antidepressant therapy have not been effective in reducing bingeing symptoms  [III] |
| **Eating-disorder not otherwise specified** | |
| **Adopt** | Patients with **subsyndromal anorexia nervosa or bulimia nervosa** who meet most but not all of the DSM-IV-TR criteria (e.g., weight >85% of expected weight, binge and purge frequency less than twice per week) merit treatment similar to that of patients who fulfill all criteria for these diagnoses.  [II] |
| **a) Binge eating disorder**  **(i) Nutritional rehabilitation and counseling** | |
| **Adopt** | **Behavioral weight control programs** incorporating low- or very-low-calorie diets may help with weight loss and usually with the reduction of symptoms of binge eating.  [I] |
| **Adopt** | It is important to **advise patients** that weight loss is often not maintained, and that binge eating may recur when weight is gained.  [I] |
| **Adopt** | It is also important to advise them that weight gain after weight loss may be accompanied by a **return of binge eating patterns**.  [I] |
| **Adopt** | **Various combinations** of diets, behavior therapies, interpersonal therapies, psychodynamic psychotherapies, non-weight-directed psychosocial treatments, and even some “non-diet/health at every size” psychotherapy approaches may be of benefit for binge eating and weight loss or stabilization.  [III] |
| **Adopt** | Patients with a history of repeated weight loss followed by weight gain **(“yo-yo” dieting)** or patients with an **early onset of binge eating** may benefit from following programs that focus on decreasing binge eating rather than on weight loss.  [II] |
| **Adopt** | There is little empirical evidence to suggest that obese binge eaters who are primarily seeking weight loss should receive different treatment than obese individuals who do not binge eat.  [I] |
| **(ii) Other psychosocial treatments** | |
| **Adopt/**  **Refer** | Substantial evidence supports the efficacy of **individual or group CBT** for the behavioral and psychological symptoms of binge eating disorder.  [I] |
| **Adopt/**  **Refer** | **IPT and dialectical behavior therapy** have also been shown to be effective for behavioral and psychological symptoms and can be considered alternatives.  [II] |
| **Adopt/**  **Refer** | Patients may be advised that some studies suggest that most patients continue to show behavioral and psychological improvement at their 1-year follow-up.  [II] |
| **Adopt/**  **Refer** | Substantial evidence supports the efficacy of **self-help and guided self-help CBT programs** and their use as an initial step in a sequenced treatment program.  [I] |
| **Adopt/**  **Refer** | Other therapies that use a **“nondiet” approach** and focus on self-acceptance, improved body image, better nutrition and health, and increased physical movement have been tried, as have addiction-based 12-step approaches, self-help organizations, and treatment programs based on the Alcoholics Anonymous model, but no systematic outcome studies of these programs are available.  [III] |
| **(iii) Medications** | |
| **Adopt** | Substantial evidence suggests that treatment with **antidepressant medications**, particularly **SSRI antidepressants**, is associated with at least a short-term reduction in binge eating behavior but, in most cases, not with substantial weight loss.  [I] |
| **Adopt** | The **medication dosage** is typically at the **high end** of the recommended range.  [I] |
| **Adopt** | The **appetite-suppressant medication sibutramine** is effective for binge suppression, at least in the short term, and is also associated with significant weight loss.  [II] |
| **Adopt** | The **anticonvulsant medication topiramate** is effective for binge reduction and weight loss, although adverse effects may limit its clinical utility for some individuals.  [II] |
| **Adopt** | **Zonisamide** may produce similar effects regarding weight loss and can also cause side effects.  [III] |
| **(iv) Combining psychosocial and medication treatments** | |
| **Adopt** | For most eating disorder patients, adding **antidepressant medication** to their behavioral weight control and/or CBT regimen **does not have a significant effect on binge suppression** when compared with medication alone. However, medications may induce additional weight reduction and have associated psychological benefits.  [II] |
| **Adopt/**  **Refer** | Adding the weight loss medication **orlistat** **to a guided self-help CBT** program may yield additional weight reduction.  [II] |
| **Adopt** | Fluoxetine in conjunction with group behavioral treatment may not aid in binge cessation or weight loss but may reduce depressive symptoms.  [II] |
| **b) Night eating syndrome** | |
| **Adopt** | **Progressive muscle relaxation** has been shown to reduce symptoms associated with night eating syndrome.  [III] |
| **Adopt** | **Sertraline** has also been shown to reduce these symptoms.  [II] |

**ACRONYMS & ABBREVIATIONS**

| AN | Anorexia Nervosa | ECG | Electrocardiogram |
| --- | --- | --- | --- |
| ARFID-CBT | Cognitive-Behavioral Therapy for Avoidant/restrictive food intake disorder | ECT | Electroconvulsive Therapy |
| ADHD | Attention Deficient Hyperactivity disorder | FDA | Food & Drug Administration |
| BMI | Body Mass Index | HRT | Hormone Replacement Therapy |
| BN-CBT | Cognitive-behavioral treatment of Bulimia Nervosa | IDA | Iron Deficiency Anemia |
| BED-CBT | Cognitive-behavioral therapy for Binge-eating disorder | IPT | Interpersonal Therapy |
| CBC | Completed Blood Count | LFTs | Liver Function Tests |
| CDC | Centers for Disease Control and Prevention | MAOIs | Monoamine oxidase inhibitors |
| DEXA | Dual-energy X-ray Absorptiometry Scan | SSRIs | Selective Serotonin Reuptake Inhibitors |
| DSM-IV-TR | Diagnostic and Statistical Manual of Mental Disorders | TSH | Thyroid Stimulating Hormone |

**SECTION 10: SUBSTANCE USE DISORDER MANAGEMENT GUIDELINES**

**Substance Use Disorder Management**

**Source guideline:** Practice Guideline for the Treatment of Patients with Substance Use Disorders 2007.^12^

**Key to understanding the strength of recommendation.**

| Strength of Recommendation | |
| --- | --- |
| [I] | Recommended with substantial clinical confidence |
| [II] | Recommended with moderate clinical confidence |
| [III] | May be recommended on the basis of individual circumstances |

**Table of Recommendations**

| **Assessment** | |
| --- | --- |
| **Adopt** | A comprehensive **psychiatric evaluation** is essential to guide the treatment of a patient with a substance use disorder  The assessment includes  1) a detailed history of the patient’s past and present substance uses and the effects of substance use on the patient’s cognitive, psychological, behavioral, and physiological functioning;  2) a general medical and psychiatric history and examination.  3) a history of psychiatric treatments and outcomes.  4) a family and social history.  5) screening of blood, breath, or urine for substance used.  6) other laboratory tests to help confirm the presence or absence of conditions that frequently co-occur with substance use disorders; and  7) with the patient’s permission, contacting a significant other for additional information.  [I] |
| **Psychiatric Management** | |
| **Adopt** | Psychiatric management is the foundation of treatment for patients with substance use disorders.  Psychiatric management has the following **specific objectives**: motivating the patient to change, establishing and maintaining a therapeutic alliance with the patient, assessing the patient’s safety and clinical status, managing the patient’s intoxication and withdrawal states, developing and facilitating the patient’s adherence to a treatment plan, preventing the patient’s relapse, educating the patient about substance use disorders, and reducing the morbidity and sequelae of substance use disorders.  [I] |
| **Specific Treatments**  **Pharmacological Treatments** | |
| **Adopt** | **Pharmacological treatments** are beneficial for selected patients with specific substance use disorders.  The categories of pharmacological treatments are  1) medications to treat intoxication and withdrawal states,  2) medications to decrease the reinforcing effects of abused substances,  3) agonist maintenance therapies,  4) antagonist therapies,  5) abstinence-promoting and relapse prevention therapies, and  6) medications to treat comorbid psychiatric conditions.  [I] |
| **Psychosocial Treatments** | |
| **Adopt/**  **Refer** | Psychosocial treatments are essential components of a **comprehensive treatment program**. Evidence-based psychosocial treatments include cognitive-behavioral therapies (CBTs, e.g., relapse prevention, social skills training), motivational enhancement therapy (MET), behavioral therapies (e.g., community reinforcement, contingency management), 12-step facilitation (TSF), psychodynamic therapy/interpersonal therapy (IPT), self-help manuals, behavioral self-control, brief interventions, case management, and group, marital, and family therapies. There is evidence to support the efficacy of integrated treatment for patients with a co-occurring substance use and psychiatric disorder; such treatment includes blending psychosocial therapies used to treat specific substance use disorders with psychosocial treatment approaches for other psychiatric diagnoses (e.g., CBT for depression).  [I] |
| **Formulation and implementation of a treatment plan** | |
| **Adopt** | The **goals of treatment** and the specific therapies chosen to achieve these goals may vary among patients and even for the same patient at different phases of an illness.  [I] |
| **Adopt** | Because many substance use disorders are chronic, patients usually require **long-term treatment**, although the intensity and specific components of treatment may vary over time.  The **treatment plan** includes the following components:  1) psychiatric management.  2) a strategy for achieving abstinence or reducing the effects or use of substances of abuse.  3) efforts to enhance ongoing adherence with the treatment program, prevent relapse, and improve functioning; and  4) additional treatments necessary for patients with a co-occurring mental illness or general medical condition.  [I] |
| **Adopt** | The **duration of treatment** should be tailored to the individual patient’s needs and may vary from a **few months to several years**.  [I] |
| **Adopt** | It is important to **intensify the monitoring** for substance use during periods when the patient is at a **high risk** of relapsing, including during the early stages of treatment, times of transition to less intensive levels of care, and the first year after active treatment has ceased.  [I] |
| **Treatment settings** | |
| **Adopt** | Treatment settings vary with regard to the availability of specific treatment modalities, the degree of restricted access to substances that are likely to be abused, the availability of general medical and psychiatric care, and the overall milieu and treatment philosophy. Patient should be treated in the **least restrictive setting** that is likely to be safe and effective.  [I]. |
| **Adopt/**  **Refer** | Commonly available **treatment settings** include hospitals, residential treatment facilities, partial hospitalization programs, and outpatient programs. Decisions regarding the site of care should be based on the patient’s ability to cooperate with and benefit from the treatment offered, refrain from illicit use of substances, and avoid high-risk behaviors as well as the patient’s need for structure and support or particular treatments that may be available only in certain settings.  [I] |
| **Adopt/**  **Refer** | Patients move from one level of care to another based on these factors and an assessment of their ability to safely benefit from a different level of care.  [I]. |
| Refer  to specialist | **Hospitalization** is appropriate for patients who  1) have a substance overdose who cannot be safely treated in an outpatient or emergency department setting.  2) are at risk for severe or medically complicated withdrawal syndromes (e.g., history of delirium tremens, documented history of very heavy alcohol use and high tolerance).  3) have co-occurring general medical conditions that make ambulatory detoxification unsafe.  4) have a documented history of not engaging in or benefiting from treatment in a less intensive setting (e.g., residential, outpatient).  5) have a level of psychiatric comorbidity that would markedly impair their ability to participate in, adhere to, or benefit from treatment or have a co-occurring disorder that by itself would require hospital-level care (e.g., depression with suicidal thoughts, acute psychosis).  6) manifest substance uses or other behaviors that constitute an acute danger to themselves or others; or 7) have not responded to or were unable to adhere to less intensive treatment efforts and have a substance use disorder(s) that endangers others or poses an ongoing threat to their physical and mental health.  [I] |
| **Adopt** | **Residential treatment** is indicated for patients who do not meet the clinical criteria for hospitalization but whose lives and social interactions have come to focus predominantly on substance use, who lack sufficient social and vocational skills, and who lack substance-free social supports to maintain abstinence in an outpatient setting.  [II] |
| **Adopt** | **Residential treatment of ≥3 months** is associated with **better long-term outcomes** in such patients.  [II] |
| **Refer** | For patients with an **opioid use disorder**, **therapeutic communities** have been found effective.  [II] |
| **Refer** | **Partial hospitalization** should be considered for patients who require intensive care but have a reasonable probability of refraining from illicit use of substances outside a restricted setting.  [II] |
| **Refer** | **Partial hospitalization** settings are frequently used for patients leaving hospitals or residential settings who remain at **high risk for relapse**. These include patients who are thought to lack sufficient motivation to continue in treatment, have severe psychiatric comorbidity and/or a history of relapse to substance use in the immediate post hospitalization or post residential period, and are returning to a high-risk environment and have limited psychosocial supports for abstaining from substance use. Partial hospitalization programs are also indicated for patients who are doing poorly despite intensive outpatient treatment.  [II] |
| **Adopt** | **Outpatient treatment** of substance use disorders is appropriate for patients whose clinical condition or environmental circumstances do not require a more intensive level of care.  [I] |
| **Adopt** | As in other treatment settings, a **comprehensive approach** is optimal, using, where indicated, a variety of psychotherapeutic and pharmacological interventions along with behavioral monitoring.  [I] |
| **Adopt/**  **Refer** | Most treatment for patients with **alcohol dependence or abuse** can be successfully conducted **outside the hospital** (e.g., in outpatient or partial hospitalization settings).  [II] |
| **Adopt** | Although patients with alcohol withdrawal must be detoxified in a setting that provides frequent clinical assessment and any necessary treatments.  [I] |
| **Adopt** | For many patients with a **cocaine use disorder**, clinical and research experience suggests the effectiveness of **intensive outpatient treatment** in which a variety of treatment modalities are simultaneously used and in which the focus is the maintenance of abstinence.  [II] |
| **Adopt** | The treatment of patients with **nicotine dependence or a marijuana use disorder** occurs on an **outpatient** basis unless patients are hospitalized for other reasons.  [I] |
| **Clinical features influencing treatment** | |
| **Adopt** | In **planning and implementing treatment**, a clinician should consider several variables with regard to patients: comorbid psychiatric and general medical conditions, gender-related factors, age, social milieu and living environment, cultural factors, gay/lesbian/bisexual/transgender issues, and family characteristics.  [I] |
| **Adopt** | Given the high prevalence of **comorbidity** of substance use disorders and other psychiatric disorders, the diagnostic distinction between substance uses symptoms and those of other disorders should receive particular attention, and specific treatment of comorbid disorders should be provided.  [I] |
| **Adopt/**  **Refer** | In addition to pharmacotherapies specific to a patient’s substance use disorder, various **psychotherapies** may also be indicated when a patient has a co-occurring psychiatric disorder, psychosocial stressors, or other life circumstances that exacerbate the substance use disorder or interfere with treatment.  [I] |
| **Adopt** | A patient’s cessation of substance use may also be associated with changes in his or her **psychiatric symptoms or the metabolism of medications** (e.g., altered antipsychotic metabolism via cytochrome P450 1A2 with smoking cessation) that will necessitate adjustment of psychotropic medication doses.  [I] |
| **Adopt** | In women of **childbearing age**, the possibility of pregnancy needs to be considered.  [I] |
| **Adopt** | Each of the substances discussed in this practice guideline has the potential to **affect the fetus**, and psychosocial treatment to encourage substance abstinence during pregnancy is recommended.  [I] |
| **Adopt** | With some substances, **concomitant agonist treatment** may be preferable to continued substance use. In pregnant smokers, treatment with nicotine replacement therapy (NRT) may be helpful.  [II] |
| **Adopt** | For pregnant women with an **opioid use disorder**, treatment with **methadone**  [I] or **buprenorphine** [II] can be a useful adjunct to psychosocial treatment. |
| **NICOTINE USE DISORDERS: TREATMENT PRINCIPLES AND ALTERNATIVES**  **Pharmacological treatments** | |
| **Adopt** | Pharmacological treatment is **recommended** for individuals who wish to stop smoking and have not achieved cessation without pharmacological agents or who prefer to use such agents.  [I] |
| **Adopt** | There are **six medications** approved by the U.S. Food and Drug Administration (FDA) for nicotine dependence, including **five NRTs** (patch, gum, spray, lozenge, and inhaler) and **bupropion**. These are all first-line agents that are equally effective in alleviating withdrawal symptoms and reducing smoking. Any of these could be used based on patient preference, the route of administration, and the side-effect profile.  [I] |
| **Adopt** | **Significant adverse events** to NRTs, including dependence, are rare. Although combined psychosocial and medication treatment produces the best outcomes in treating nicotine use disorders, these medications are effective even when no psychosocial treatment is provided.  [I] |
| **Adopt** | Using a **combination** of these first-line treatments may also improve outcome.  [II] |
| **Adopt** | **Nortriptyline and clonidine** have utility as **second-line agents** but appear to have more side effects. Other medications and acupuncture have not been proven to be effective.  [II] |
| **Psychosocial treatments** | |
| **Adopt/**  **Refer** | Psychosocial treatments are also effective for the treatment of **nicotine dependence** and include CBTs  [I] |
| **Adopt/**  **Refer** | behavioral therapies [I] |
| **Adopt/**  **Refer** | brief interventions [II] |
| **Adopt/**  **Refer** | and MET [II] |
| **Adopt/**  **Refer** | provided in individual [I] |
| **Adopt/**  **Refer** | group [I] |
| **Adopt/**  **Refer** | or telephone [I] |
| **Adopt** | formats or via self-help materials [III] |
| **Adopt** | and Internet-based formats [III]. The efficacy of treatment is related to the amount of psychosocial treatment received. The 12-step programs, hypnosis, and inpatient therapy have not been proven effective. |
| **ALCOHOL USE DISORDERS: TREATMENT PRINCIPLES AND ALTERNATIVES**  **Management of intoxication and withdrawal** | |
| **Adopt** | The acutely intoxicated patient should be **monitored and maintained** in a safe environment.  [II] |
| **Adopt** | Symptoms of **alcohol withdrawal** typically begin within **4–12 hours after cessation** or reduction of alcohol use, **peak** in intensity during the **second day** of abstinence, and generally **resolve within 4–5 days**. Serious complications include seizures, hallucinations, and delirium. The treatment of patients in moderate to severe withdrawal includes efforts to reduce central nervous system (CNS) irritability and restore physiological homeostasis.  [I] |
| **Adopt** | and generally, requires the use of thiamine and fluids [I], |
| **Adopt** | benzodiazepines [I], |
| **Adopt** | and, in some patients, other medications such as anticonvulsants, clonidine, or antipsychotic agents.  [II]. |
| **Adopt** | Once clinical stability is achieved, the **tapering of benzodiazepines and other medications** should be carried out as necessary, and the patient should be observed for the reemergence of withdrawal symptoms and the emergence of signs and symptoms suggestive of co-occurring psychiatric disorders [I] |
| **Pharmacological treatments** | |
| **Adopt** | Specific pharmacotherapies for alcohol-dependent patients have well-established efficacy and moderate effectiveness. **Naltrexone** may attenuate some of the reinforcing effects of alcohol.  [I] |
| **Adopt** | although data on its long-term efficacy are limited. The use of long-acting, injectable naltrexone may promote adherence, but published research is limited, and FDA approval is pending. **Acamprosate**, a γ-aminobutyric acid (GABA) analog that may decrease alcohol craving in abstinent individuals, may also be an effective adjunctive medication in motivated patients who are concomitantly receiving psychosocial treatment.  [I] |
| **Adopt** | **Disulfiram** is an effective adjunct to a comprehensive treatment program for reliable, motivated patients whose drinking may be triggered by events that suddenly increase alcohol craving.  [II] |
| **Psychosocial treatments** | |
| **Adopt**/  **Refer** | Psychosocial treatments found effective for some patients with an alcohol use disorder include MET.  [I], |
| **Adopt**/  **Refer** | CBT [I], |
| **Adopt**/  **Refer** | behavioral therapies [I], |
| **Adopt**/  **Refer** | TSF [I], |
| **Adopt**/  **Refer** | marital and family therapies [I], |
| **Adopt**/  **Refer** | group therapies [II], |
| **Adopt**/  **Refer** | and psychodynamic therapy/IPT [III]. |
| **Adopt**/  **Refer** | Recommending that patients participate in self-help groups, such as Alcoholics Anonymous (AA), is often helpful.  [I] |
| **MARIJUANA USE DISORDERS: TREATMENT PRINCIPLES AND ALTERNATIVES** | |
| **Adopt** | Studies of treatment for marijuana use disorders are limited. **No specific pharmacotherapies** for marijuana withdrawal or dependence can be recommended.  [I] |
| **Adopt**/  **Refer** | In terms of **psychosocial therapies**, an intensive relapse prevention approach that combines motivational interventions with the development of coping skills may be effective for the treatment of marijuana dependence, but further study of these approaches is necessary.  [III] |
| **COCAINE USE DISORDERS: TREATMENT PRINCIPLES AND ALTERNATIVES**  **Management of intoxication and withdrawal** | |
| **Adopt** | Cocaine intoxication is usually self-limited and typically requires only **supportive care**.  [II] |
| **Adopt** | However, **hypertension, tachycardia, seizures, and persecutory delusions** can occur with cocaine intoxication and may require specific treatment.  [II] |
| **Adopt** | Acutely agitated patients may benefit from **sedation with benzodiazepines**.  [III] |
| **Pharmacological treatments** | |
| **Adopt** | Pharmacological treatment is not ordinarily indicated as an initial treatment for patients with cocaine dependence. In addition, **no pharmacotherapies have FDA indications** for the treatment of cocaine dependence. However, for individuals who fail to respond to psychosocial treatment alone, some medications (topiramate, disulfiram, or modafinil) may be promising when integrated into psychosocial treatments. |
| **Psychosocial treatments** | |
| **Adopt/**  **Refer** | For many patients with a cocaine use disorder, **psychosocial treatments** focusing on abstinence are effective.  [I] |
| **Adopt/**  **Refer** | In particular, CBTs [I], |
| **Adopt/**  **Refer** | behavioral therapies [I], |
| **Adopt/**  **Refer** | and 12-step-oriented individual drug counseling can be useful, although efficacy of these therapies varies across subgroups of patients.  [I] |
| **Adopt/**  **Refer** | Recommending regular participation in a **self-help group** may improve the outcome for selected patients with a cocaine use disorder.  [III] |
| **OPIOID USE DISORDERS: TREATMENT PRINCIPLES AND ALTERNATIVES**  **Management of intoxication and withdrawal** | |
| **Adopt** | Acute opioid intoxication of a **mild to moderate** degree usually **does not require specific treatment**.  [II] |
| **Refer** | However, **severe opioid overdose**, marked by **respiratory depression**, may be fatal and requires treatment in an **emergency department or inpatient setting**.  [I] |
| **Adopt** | **Naloxone** will **reverse respiratory depression** and other manifestations of opioid overdose.  [I] |
| **Adopt** | The treatment of opioid withdrawal is directed at safely **ameliorating acute symptoms** and facilitating the patient’s entry into a long-term treatment program for opioid use disorders.  [I] |
| **Adopt** | Strategies found to be effective include **substitution of methadone or buprenorphine** for the opioid followed by gradual tapering.  [I] |
| **Adopt** | abrupt discontinuation of opioids, with the use of clonidine to suppress withdrawal symptoms.  [II] |
| **Adopt** | and clonidine-naltrexone detoxification.  [II] |
| **Adopt** | It is essential that the treating physician assess the patient for the **presence of other substances**, particularly alcohol, benzodiazepines, or other anxiolytic or sedative agents, because the concurrent use of or withdrawal from other substances can complicate the treatment of opioid withdrawal.  [I] |
| **Pharmacological treatments** | |
| **Adopt** | **Maintenance treatment** with **methadone or buprenorphine** is appropriate for patients with a prolonged history (>1 year) of opioid dependence.  [I] |
| **Adopt** | The **goals of treatment** are to achieve a stable maintenance dose of opioid agonist and facilitate engagement in a comprehensive program of rehabilitation.  [I] |
| **Adopt** | **Maintenance treatment** with **naltrexone** is an alternative strategy, although the utility of this strategy is often limited by lack of patient adherence and low treatment retention.  [I] |
| **Psychosocial treatments** | |
| **Adopt/**  **Refer** | Psychosocial treatments are effective components of a comprehensive treatment plan for patients with an opioid use disorder.  [II] |
| **Adopt/**  **Refer** | Behavioral therapies (e.g., contingency management) [II] |
| **Adopt/**  **Refer** | CBTs [II] |
| **Adopt/**  **Refer** | psychodynamic psychotherapy [III] |
| **Adopt/**  **Refer** | and group and family therapies [III] |
| **Adopt/**  **Refer** | have been found to be effective for some patients with an opioid use disorder. Recommending regular participation in self-help groups may also be useful.  [III] |

**ACRONYMS & ABBREVIATIONS**

| CNS | Central Nervous System | IPT | Interpersonal Therapy |
| --- | --- | --- | --- |
| CBT | Cognitive Behavioral Therapy | MET | Motivational Enhancement Therapy |
| FDA | Food & Drug Administration | NRT | Nicotine Replacement Therapy |
| GABA | γ-Aminobutyric acid | TSF | Twelve-Step Facilitation |

**References:**

1. Griffiths P. Evidence informing practice: introducing the mini-review. Br J Community Nurs. 2002;**7**(1):38-9.

2. Practice guideline for the treatment of patients with bipolar disorder (revision). Am J Psychiatry. 2002;**159**(4 Suppl):1-50.

3. Koran LM, Hanna GL, Hollander E, Nestadt G, Simpson HB. Practice guideline for the treatment of patients with obsessive-compulsive disorder. Am J Psychiatry. 2007;**164**(7 Suppl):5-53.

4. Keepers GA, Fochtmann LJ, Anzia JM, Benjamin S, Lyness JM, Mojtabai R, et al. The American Psychiatric Association Practice Guideline for the Treatment of Patients With Schizophrenia. Am J Psychiatry. 2020;**177**(9):868-72.

5. Ursano RJ, Bell C, Eth S, Friedman M, Norwood A, Pfefferbaum B, et al. Practice guideline for the treatment of patients with acute stress disorder and posttraumatic stress disorder. Am J Psychiatry. 2004;**161**(11 Suppl):3-31.

6. Gelenberg AJ. The American Psychiatric Association Treatment Guideline for Major Depressive Disorder: process and content: Ann Gen Psychiatry. 2010 Apr 22;**9**(Suppl 1):S46. doi: 10.1186/1744-859X-9-S1-S46. eCollection 2010.

7. Stein MB, McIntyre JS. Treatment of Patients With Panic Disorder. Retrieved; 2010.

8. Trzepacz P, Breitbart W, Franklin J, Levenson J, Martini DR, Wang P. Treatment of patients with delirium. Practice Guideline for the treatment of patients with delirium Trzepacz PT, cheir American Psychiatric Association, APA Press. 2010.

9. Rabins PV, Blacker D, Rovner BW, Rummans T, Schneider LS, Tariot PN, et al. American Psychiatric Association practice guideline for the treatment of patients with Alzheimer's disease and other dementias. The American journal of psychiatry. 2007;**164**(12 Suppl):5-56.

10. Hort J, O'Brien JT, Gainotti G, Pirttila T, Popescu BO, Rektorova I, et al. EFNS guidelines for the diagnosis and management of Alzheimer's disease. Eur J Neurol. 2010;**17**(10):1236-48.

11. Treatment of patients with eating disorders,third edition. American Psychiatric Association. Am J Psychiatry. 2006;**163**(7 Suppl):4-54.

12. Kleber HD, Weiss RD, Anton RF, Jr., George TP, Greenfield SF, Kosten TR, et al. Treatment of patients with substance use disorders, second edition. American Psychiatric Association. Am J Psychiatry. 2007;**164**(4 Suppl):5-123.
